# Revitalizing contaminated soils: The combined power of modified biochar and intrinsic bacteria for heavy metal and petroleum hydrocarbon removal and plants performance

**DOI:** 10.1371/journal.pone.0349599

**Published:** 2026-06-24

**Authors:** Zahra Dianat Maharlouei, Davood Azadi, Majid Fekri

**Affiliations:** 1 Department of soil science, Faculty of Agriculture, Shahid Bahonar University of Kerman, Kerman, Iran; 2 Department of Microbiology, School of Medicine, Baqiyatallah University of Medical Sciences, Tehran, Iran; Portuguese Catholic University: Universidade Catolica Portuguesa, PORTUGAL

## Abstract

Soil contamination with heavy metals and petroleum hydrocarbons poses a critical environmental challenge, threatening food security, human health, and ecosystem sustainability in various regions by reducing crop yields and introducing toxic pollutants into the food chain. Therefore, sustainable remediation strategies are essential to protect agricultural productivity. This study aimed to isolate native bacteria capable of degrading these contaminants from Kerman’s polluted soils and assess their synergistic effects with microbially modified biochar (MB) on soil bioremediation, quality enhancement, and maize performance. A total of 30 soil samples were collected from industrially contaminated sites in Kerman, Iran, and analyzed to isolate native bacterial species using biochemical and molecular tests. Biochars, prepared from rice husk and inoculated with the bacterial consortium, and were evaluated in a factorial greenhouse experiment. The experiment utilized a completely randomized design with 90 pots containing 4 kg of artificially contaminated soil (Cr, Pb, Cd, Cu; petroleum hydrocarbons) and four treatments: control, pristine biochar (PB), bacterial inoculants, and MB. Maize was grown for 90 days under controlled conditions, and soil and plant parameters, including physicochemical properties, contaminant levels, growth characteristics, transfer factor (TF), and bioaccumulation factor (BAF), were assessed. Five bacterial species (*P. fluorescens*, *R. qingshengii*, *B. metallica*, *B. cereus*, *S. pactum*) were isolated from 30 soil samples and tested with MB in a greenhouse experiment. Results showed MB reduced heavy metal bioavailability by 45–55% and hydrocarbons by 70% (p < 0.001), enhanced soil organic carbon, lowered metal uptake (e.g., TF for Cd: 0.27 vs. 0.95), and increased maize biomass (shoot: 30 g, root: 18 g vs. 15 g, 8 g, p < 0.001), offering a sustainable remediation strategy. These findings demonstrate the potential of integrating novel bacterial strains with modified biochar for effective, sustainable soil remediation for arid regions like Kerman, Iran.

## Introduction

Soil contamination with heavy metals (chromium, lead, cadmium, copper) and petroleum hydrocarbons, resulting from industrial activities, mining, agricultural practices, and urbanization, poses severe threats to ecosystem health, food security, and human wellbeing [1]. These pollutants are persistent, bioaccumulative, and toxic, disrupting soil microbial communities, reducing plant productivity, and entering the food chain, which can lead to chronic health issues such as neurological disorders and cancer [2]. For instance, lead (Pb) accumulation in soils has been linked to cognitive impairments in children, while polycyclic aromatic hydrocarbons (PAHs) from petroleum are known carcinogens [3]. Conventional remediation methods, such as chemical leaching, soil washing, and thermal desorption, are often prohibitively expensive, energy intensive, and environmentally disruptive, as they can degrade soil structure and fertility [3]. These limitations have driven the exploration of sustainable, cost effective alternatives, such as bioremediation using pollutant degrading microorganisms and the application of biochar as a soil amendment [4].

Biochar, a carbon rich material produced through biomass pyrolysis, has been widely recognized as a multifunctional amendment for soil remediation due to its high surface area, porous structure, and abundance of functional groups [5]. These properties enhance the immobilization of heavy metals and organic pollutants by reducing their mobility and bioavailability. For example, Houssou et al. [6] reported that hardwood biochar reduced bioavailable cadmium (Cd) by 50% in contaminated soils. Beyond pollutant immobilization, biochar improves soil physicochemical attributes such as water holding capacity, cation exchange capacity, and microbial activity, while also contributing to long term carbon sequestration [7]. Modified biochars, including NaOH treated and Fe₃O₄ enriched materials, further increase adsorption capacity through enhanced functional groups and magnetic separation properties [8]. NaOH modification has been shown to boost the binding of Pb and Cu in calcareous soils of Kerman Province in Iran by increasing oxygen containing functional groups [9].

Bioremediation, using microorganisms capable of hydrocarbon degradation and heavy metal detoxification, provides an ecofriendly and cost effective approach that maintains soil structure and function [10]. Bacterial taxa such as *Rhodococcus, Pseudomonas,* and *Bacillus* spp. have demonstrated the ability to degrade PAHs, biosorb heavy metals, and reduce plant metal uptake through biosurfactants production, enzymatic oxidation, and metal complexation [11–15]. However, the efficiency of microbial remediation can be constrained by environmental stresses such as high pH, low nutrient availability, and extreme pollutant concentrations—conditions commonly found in calcareous soils [16]. Consequently, combining biochar with pollutant degrading microbial inoculants is increasingly viewed as a promising strategy for enhancing microbial survival and activity, while simultaneously reducing pollutant bioavailability [17].

Iran faces substantial soil contamination challenges due to extensive mining, petroleum extraction, and industrial activities [18]. Provinces such as Kerman, Isfahan, and Khuzestan exhibit heavy metal and hydrocarbon levels that exceed international safety limits, with Cu and Pb concentrations reaching 500 mg kg ⁻ ¹ and 300 mg kg ⁻ ¹ near the Sarcheshmeh Copper Mine, and PAHs levels up to 5000 mg kg ⁻ ¹ in oil producing regions of Khuzestan [19–21]. The prevalence of calcareous soils with high pH and low organic matter further complicates remediation, as these soils influence metal mobility and microbial activity. Given that agriculture contributes approximately 10% of Iran’s GDP and supports more than 85 million people, the need for sustainable, locally tailored remediation strategies is urgent [22].

Recent studies (2022–2025) have demonstrated the benefits of integrating biochar with microbial inoculants; however, most investigations have relied on commercial strains, generic biochar modifications, or experiments conducted in temperate or acidic soil environments [23,24]. There remains a significant gap in understanding how locally adapted microbial consortia interact with modified biochar under the extreme conditions of heavily co contaminated calcareous soils typical of Iran’s arid and semi-arid regions. Addressing this gap requires site specific approaches that simultaneously enhance metal immobilization, support microbial survival, and stimulate hydrocarbon degradation.

Given these challenges, the present study aimed to: (i) characterize the physicochemical and contamination profile of soils from Kerman Province, (ii) isolate and identify indigenous bacterial species with high resistance to heavy metals and petroleum hydrocarbons, and (iii) evaluate the combined and individual effects of NaOH modified rice husk biochar and native bacterial consortia on pollutant bioavailability, soil microbial activity, and maize growth under greenhouse conditions. By integrating soil characterization with mechanistic remediation assessments, this work seeks to offer a practical, scalable, and locally adapted framework for restoring co contaminated calcareous soils.

## Materials and methods

### Soil characterization

**Fig 1:**
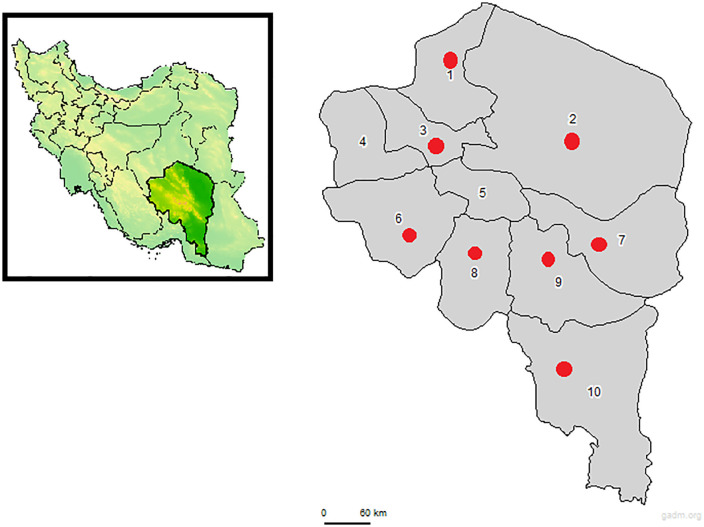
Spot map illustrating the eight industrially contaminated sites in Kerman Province, Iran (30°17’N, 57°04’E), where 30 soil samples were collected from a depth of 3–5 cm between September 2020 and January 2021. The Fig was created using QGIS open source software (version 3.28, qgis.org; GPL license) and finalized in Adobe Photoshop 2021 (v18.1.1.252) for visual clarity. The administrative boundaries of Iran’s provinces and counties were derived from GADM database of Global Administrative Areas (version 4.1, https://gadm.org), which provides openly licensed data compatible with Creative Commons Attribution (CC BY 4.0) terms. The base map layer, including topographic and national boundaries, was sourced from Natural Earth (public domain; raster data at 1:10m scale, https://www.naturalearthdata.com).All geospatial data and graphical components are freely available and reproducible under open licenses, ensuring full compliance with the PLOS CC BY 4.0 policy.

From September 2020 to January 2021, in a cross sectional study, a total of 30 soil samples (15–30 g each, collected from a depth of 3–5 cm) were obtained from eight industrially contaminated sites around Kerman, Iran (30°17’N, 57°04’E), focusing on areas impacted by mining and industrial activities. The sampling sites are detailed in Fig 1. Samples were immediately transferred to the microbiology laboratory at Shahid Bahonar University of Kerman for processing and analysis. For bacterial isolation, five grams of each soil sample were placed into a 50 ml sterile centrifuge tube, mixed with 20 ml of sterile distilled water, vortexed for 5 minutes, and centrifuged at 5000 × g (Universal HB320, Iran) at room temperature for 15 minutes. The supernatant and pellet were separately decontaminated using 1% NaOH and 3% sodium lauryl sulfate to reduce microbial contamination and facilitate the isolation of heavy metal and hydrocarbon degrading bacteria. The processed samples were then used for bacterial screening and further physicochemical analysis.

### Ethical approvals and field permits

All experimental procedures, including microbial isolation from soil samples and in vitro assays, were conducted in accordance with the ethical guidelines of the Ethics Committee in Research of Shahid Bahonar University of Kerman (Approval No. EC-SBU-2020–045, 2020). This committee, established under the university’s research governance framework, reviewed and approved the study protocol to ensure compliance with national and international standards for biosafety and environmental impact. Field site access for soil sampling was authorized by the Department of Soil Science and Microbiology at Shahid Bahonar University of Kerman, as the sites were located on university owned experimental farmlands in Kerman province, Iran. No additional permits from external authorities, such as the Iranian Department of Environment, were required, as the sampling was non destructive, limited to surface soils (<20 cm depth), and fell under exemptions for academic research on institutional land per the Environmental Protection Act of Iran (Article 12, 2019). All activities adhered to the Declaration of Helsinki principles for research integrity.

#### Physical and chemical analysis of soil.

Soil physicochemical properties were determined following standard protocols. Moisture content was measured gravimetrically by drying samples at 105°C for 24 hours. Heavy metal concentrations (Cr, Pb, Cd, Cu) were quantified using Atomic Absorption Spectrometry (AAS) (PinAAcle 900T system (USA) after aqua regia digestion. Petroleum hydrocarbons were analyzed via gas chromatography mass spectrometry (GC-MS) (Agilent 7890B, USA) [25,26]. Soil pH and electrical conductivity (EC) were assessed in a 1:5 soil: water slurry using a Hanna HI9813 device (USA). Total carbon was determined by dry combustion with an Elementary Vario EL III analyzer (Germany), while available potassium (K) and phosphorus (P) were extracted using the ammonium acetate and Olsen methods, respectively, and measured via flame photometry (Jenway PFP7, UK) for K and spectrophotometry (Shimadzu UV-1800, Japan) for P [27]. The details of soil samples analyzed in this study current are given in Table 1.

**Table 1:** Characteristics of soil samples collected from industrially contaminated sites in Kerman, Iran.

Sampling Site	Number of Samples per Location	Type of Soil*	Soil Region (Depth)	CFU g ⁻ ¹	Common Microorganisms in Soil	Heavy Metal (mg kg^-1^)	Petroleum Hydrocarbon (mg kg-1)	K (mg kg ⁻ ¹)	P (mg kg ⁻ ¹)***	pH	Electrical Conductivity (dS/m)	Organic Carbon (%)	Total Nitrogen (mg kg^-1^)
1	4	Aridisols	Horizon A (3–5 cm)	2.0 × 10^4^ - 6.0 × 10^4^	*B. subtilis*, *P. aeruginosa*, *R. erythropolis*, *S. griseus*	Cr: 80, Pb: 300, Cd: 40, Cu: 100	600	190	14	7.6	2.0	1.1	40
2	4	Aridisols	Horizon A (3–5 cm)	5.0 × 10^4^ - 8.0 × 10^4^	*R.s qingshengii*, *P. fluorescens*, *B. cepacia*, *A. globiformis*	Cr: 90, Pb: 350, Cd: 45, Cu: 110	700	175	16	7.8	2.2	1.3	45
3	4	Mollisols	Horizon A (3–5 cm)	1.0 × 10^5^ - 3.0 × 10^5^	*B.metallica*, *S. pactum*, *B. cereus*, *P. putida*	Cr: 70, Pb: 280, Cd: 35, Cu: 90	500	200	12	7.5	1.9	1.0	38
4	4	Aridisols	Horizon A (3–5 cm)	2.5 × 10^5^ - 4.0 × 10^5^	*B. megaterium*, *P. stutzeri*, *A. baumannii*,	Cr: 100, Pb: 400, Cd: 50, Cu: 120	800	185	15	8.0	2.3	1.2	50
5	4	Mollisols	Horizon A (3–5 cm)	4.0 × 10^5^ - 6.0 × 10^5^	*R. ruber*, *B. gladioli*, *S. coelicolor*, *P. mendocina*	Cr: 60, Pb: 250, Cd: 30, Cu: 80	400	170	13	7.7	2.1	0.9	35
6	3	Aridisols	Horizon A (3–5 cm)	6.0 × 10^5^ - 9.0 × 10^5^	*P. fluorescens*, *B. licheniformis*, *S. paucimobilis*, *M. smegmatis*	Cr: 85, Pb: 320, Cd: 40, Cu: 105	650	195	17	7.9	2.4	1.4	48
7	3	Mollisols	Horizon A (3–5 cm)	8.0 × 10^5^ - 1.2 × 10^6^	*S. pactum*, *R. jostii*, *P. alcaligenes*, *N. asteroides*	Cr: 75, Pb: 290, Cd: 38, Cu: 95	550	180	14	7.6	2.0	1.1	42
8	4	Aridisols	Horizon A (3–5 cm)	1.0 × 10^6^ - 1.8 × 10^6^	*B. metallica*, *P. chlororaphis*, *B. pumilus*, *A. viscosus*	Cr: 95, Pb: 380, Cd: 48, Cu: 115	750	190	16	8.1	2.5	1.3	47
Error	–	–	–	–	–	3.5	5.0	2.0	0.5	0.1	0.02	0.05	1.0

*Aridisols: Soils formed in arid regions with very low organic matter, limited leaching, and often containing accumulations of salts or carbonates. Mollisols: Fertile, dark colored soils rich in organic matter, typically formed under grasslands, with high base saturation and excellent agricultural productivity.

**CFU: colony forming units

***EC: electrical conductivity

****Ppm: Part per million

### Bacterial isolation and identification

#### Isolation and conventional of biodegradable strains.

Bacterial isolation was performed using a serial dilution technique to ensure a broad representation of microbial diversity [28]. Initially, 1 g of pellet and 1 ml supernatant from each decontaminated soil sample was suspended in 9 ml of sterile 0.85% saline solution (Merck, Germany) in a 15 ml sterile Falcon tube (Corning, USA). The suspension was vortexed vigorously for 2 minutes using a Vortex Genie 2 (Scientific Industries, USA) to homogenize the sample and release microbial cells. Serial dilutions were prepared by transferring 1 ml of the initial suspension into 9 ml of sterile saline, repeated up to 10^6^ dilutions, to reduce microbial density and facilitate colony isolation. From each dilution, 100 µl aliquots were spread plated in triplicate onto nutrient agar (NA) plates (HiMedia, India) supplemented with heavy metals (growth at 50 mg/L of Cr, Pb, Cd, Cu (Sigma-Aldrich, USA)) or 1% (v/v) crude oil (obtained from the National Iranian Oil Company, Iran) to select for tolerant strains. The plates were incubated at 30°C for 48 hours in an incubator (Member IN55, Germany). After incubation, distinct colonies were counted to determine the colony forming units (CFU) per gram of soil, and morphologically different colonies were selected for further screening. The isolated colonies were screened for their degradation potential using a combination of biochemical and analytical methods. Biochemical characterization was conducted using the API 20E system (bioMérieux, France). Based on the biochemical and hydrocarbon degradation screening, isolates exhibiting the highest tolerance to heavy metals (growth at 50 mg/L of Cr, Pb, Cd, Cu) and the most significant hydrocarbon degradation (at least 50% reduction in C10–C30 alkanes) were selected for identification. The top performing isolates were identified using 16S rRNA gene sequencing.

#### Molecular isolation of biodegradable strains.

Chromosomal DNA of isolates was extracted using simple boiling method described by Azadi et al [29,30]. In brief, a few colony of bacteria grown on Sutton medium was added to 200 µl of TE buffer (1mM EDTA, 10mM Tris [pH 8]) and boiled for 15 minutes, then microtube was placed in the −20 ° C freezer for 20 minutes, this procedure was repeated twice. Afterwards the suspension was centrifuged at 8000 for 10 min and supernatant was transferred to a new microtube and centrifuged at 13,000 × g for 20 min. The pellet was resuspended in 50 ml of TE buffer and stored at − 20 °C. The 16S rRNA gene was amplified using universal primers 27F (5’-AGAGTTTGATCMTGGCTCAG-3’) and 1492R (5’-TACGGYTACCTTGTTACGACTT-3’) (Pishgam Biotech, Iran) in a 25 µl PCR reaction containing 12.5 µl of 2 × DreamTaq Green PCR Master Mix (SamBio, South Korea), 1 µl of each primer (10 µM), 2 µl of DNA template (50 ng/µl), and 8.5 µl of nuclease free water. The PCR was performed in a T100 Thermal Cycler (Bio Rad, USA) with the following conditions: initial denaturation at 95°C for 3 minutes, followed by 35 cycles of 95°C for 30 seconds, 55°C for 30 seconds, and 72°C for 90 seconds, with a final extension at 72°C for 5 minutes. The PCR products were purified and sequenced using the Sanger method by Pishgam Biotech Inc. (Iran). The sequences were analyzed using the BLASTn tool (NCBI, USA) and compared against the GenBank database to identify the isolates at the species level with a similarity threshold of ≥97%. Phylogenetic analysis was conducted using MEGA 11 software (USA) to construct a neighbor joining tree with 1000 bootstrap replicates, confirming the taxonomic assignment.

### Bioremediation analysis

The bioremediation capacity of the strains isolated from environmental samples in the current study was assessed based on method described by Kanaly et al [31]. The details are as follows:

#### Chemicals and media for bioremediation studies.

A petroleum hydrocarbon mixture containing phenanthrene, pyrene, anthracene, and fluoranthene (AccuStandard, Spain) was prepared at 0.2 mg/ml in a 1:1 (v/v) solution of dichloromethane and methanol (Merck, Germany). Stock solutions of heavy metals (Cr as K₂Cr₂O₇, Pb as Pb(NO₃) ₂, Cd as CdCl₂, Cu as CuSO₄, Sigma-Aldrich, USA) were prepared at 10 mg/ml in deionized water. A Mineral Salt Medium (MSM) was used for bioremediation experiments, consisting of the following per liter: 0.25 g MgSO₄·7H₂O, 0.5 g KH₂PO₄, 0.5 g K₂HPO₄, 1 g NaCl, 0.009 g CaCl₂·2H₂O, 0.5 g KNO₃, 0.1 g MnCl₂·4H₂O, 0.07 g ZnCl₂, 0.015 g CuCl₂·2H₂O, 0.025 g NiCl₂·6H₂O, 0.12 g CoCl₂·6H₂O, and 0.025 g Na₂MoO₄·2H₂O (all from Merck, Germany). The MSM was autoclaved at 121°C for 15 minutes (Tomy SX-500, Japan) prior to use.

#### Bioremediation capability assessment.

The bioremediation potential of the selected bacterial isolates was assessed by inoculating 1 ml of a 0.5 McFarland turbidity suspension (1 × 10⁸ CFU/ml) into 100 ml of MSM supplemented with 1% petroleum hydrocarbon mixture and 1% heavy metal mixture (50 mg/L each of Cr, Pb, Cd, Cu) in 250 ml Erlenmeyer flasks. The flasks were incubated at 30°C for 6 days on an orbital shaker (Arta 55SIN, Iran) at 90 rpm. Bacterial growth was monitored daily by measuring optical density (OD) at 560 nm using a spectrophotometer (Shimadzu UV-1800, Japan). An increase in OD indicated microbial growth and potential pollutant degradation. After 6 days, 5 ml samples were analyzed for petroleum hydrocarbon and heavy metal degradation using GC-MS and AAS, respectively.

**Determination of petroleum hydrocarbon degradation:** For petroleum hydrocarbon analysis, 5 ml of MSM was transferred to screw cap glass tubes, and 0.6 ml of tetrachloroethylene: methanol (1:100 v/v, Merck, Germany) was added as the extraction solvent. Samples were vortexed for 10 seconds, centrifuged at 3000 × g for 10 minutes (Universal HB320, Iran), and the organic phase was analyzed via GC-MS (Agilent 7890B, USA) using an HP-5MS column (30 m × 0.25 mm, 0.25 µm film thickness, Agilent, USA). The oven temperature increased from 60°C (held for 2 minutes) to 280°C at 10°C/min, held for 10 minutes, with helium (Air Liquide, France) as the carrier gas at 1 ml/min. The injector was set at 250°C in splitless mode, and the mass spectrometer operated in EI mode at 70 eV. Hydrocarbons were identified and quantified using retention times, mass spectra, and calibration curves from standards, with degradation efficiency calculated by comparing residual concentrations to initial levels [25].

**Determination of heavy metal degradation:** For heavy metal analysis, 5 ml of MSM was transferred to screw cap glass tubes, and 0.6 ml of aqua regia (HNO₃: HCl, 3:1 v/v, Merck, Germany) was added. Samples were digested at 95°C for 30 minutes in a water bath, cooled, filtered through a 0.45 µm membrane filter (Millipore, USA), and diluted for analysis. Heavy metal concentrations (Cr, Pb, Cd, Cu) were measured using AAS (PinAAcle 900T, USA) and compared to a control MSM without bacterial inoculation to determine biosorption or degradation efficiency [26].

**Bioremediation efficiency assessment:** The efficiency of bioremediation was monitored by measuring the degradation of total petroleum hydrocarbons (TPH) and the reduction of heavy metals in the soil. TPH concentrations were quantified using GC-MS, and heavy metal concentrations were determined by AAS. Samples were taken at regular intervals (0, 15, 30, 60, and 90 days) to track the rate of contaminant degradation. Biodegradation rates were calculated based on the percentage reduction in TPH and heavy metal content compared to the initial concentrations [26,32].

### Experimental design and conditions

The experiments were conducted under controlled laboratory conditions at the Soil Microbiology Laboratory of Shahid Bahonar University of Kerman, Iran, to ensure precise control over environmental variables such as temperature (25 ± 1°C), humidity (50 ± 5%), and light (16:8 h light dark cycle). The study was designed to evaluate the efficacy of iron oxide modified biochar combined with intrinsic bacterial communities (*Pseudomonas* sp. and *Bacillus* sp.) for heavy metal remediation in arid soils from Kerman province. A completely randomized design was employed, with three biological replicates per treatment to account for biological variability. Each biological replicate included five technical repeats to enhance measurement precision, resulting in a total of 15 data points per treatment. Sample sizes were determined based on a power analysis targeting a statistical power of 0.8 and an alpha level of 0.05 to detect significant differences in heavy metal immobilization and microbial activity. Soil samples were incubated in sterile microcosms (100 g soil per replicate) under controlled conditions, and all experiments adhered to standardized protocols for microbial and soil analysis, ensuring reproducibility and statistical reliability of the findings.

### Biochar preparation and modification

Pristine biochar (PB) was produced from rice husk and almond soft husk, sourced from Kerman, Iran [9]. The feedstocks were air dried at 25°C for 48 hours, ground to <2 mm using a laboratory mill (Retsch SM 200, Germany), and washed with deionized water (Milli Q, Millipore, USA) to remove impurities. Pyrolysis was performed in a muffle furnace (Nabertherm B410, Germany) at 500°C for 2 hours under limited oxygen conditions (N₂ flow at 100 ml/min, Air Liquide, France) with a heating rate of 10°C/min. The biochar was cooled under N₂ flow, sieved to 0.5–1 mm (ASTM standard sieves, USA), and stored at 4°C, yielding 35 ± 3% for rice husk and 38 ± 2% for almond soft husk. The pristine biochar was then modified with the selected bacterial isolates from the previous stages. Biochar (10 g) was mixed with 100 ml of a 0.5 McFarland suspension (1 × 10⁸ CFU/ml) of the selected isolates in sterile 0.85% saline (Merck, Germany), incubated at 30°C for 48 hours with shaking at 150 rpm (IKA RW 20, Germany), filtered using Whatman No. 1 filter paper (UK), washed with deionized water, and dried at 40°C for 24 hours (Memmert UN55, Germany) to produce MB.

### Physicochemical characterization of Biochar

#### Surface area and porosity.

The surface area and porosity of PB and MB were determined using the Brunauer Emmett Teller (BET) method with a Micrometrics ASAP 2020 analyzer (USA). Samples (0.2 g) were degassed at 200°C for 6 hours under vacuum to remove adsorbed gases and moisture. Nitrogen adsorption desorption isotherms were measured at 77 K, with the specific surface area calculated using the BET equation and pore volume determined via the Barrett Joyner Halenda (BJH) method. Measurements were conducted in triplicate, ensuring reproducibility within ±5% standard deviation.

#### Functional groups.

Functional groups on PB and MB were analyzed using Fourier transform infrared spectroscopy (FT-IR) with a Bruker Vertex V70 spectrometer (Germany). Biochar samples (2 mg) were mixed with 200 mg of spectroscopic grade KBr (Merck, Germany) and pressed into pellets under 10 tons of pressure. Spectra were recorded in the range of 400–4000 cm ⁻ ¹ at a resolution of 4 cm ⁻ ¹, with 32 scans averaged per sample to enhance signal clarity. Background spectra of pure KBr were subtracted to isolate biochar specific peaks. Triplicate measurements were performed to confirm consistency.

#### Thermal stability.

Thermal stability was assessed via thermogravimetric analysis (TGA) using a TA Instruments Q500 analyzer (USA). Approximately 10 mg of each biochar sample was placed in an alumina crucible and heated from 25°C to 800°C at a rate of 10°C/min under a nitrogen flow of 60 ml/min to prevent oxidation. Weight loss was recorded continuously, and the percentage of mass retained at 600°C was calculated to evaluate thermal stability. Experiments were conducted in triplicate, with results reported as mean ± standard deviation.

#### Elemental composition and morphology.

Elemental composition and surface morphology were evaluated using field emission scanning electron microscopy with energy dispersive X-ray spectroscopy (FESEM-EDS) (TESCAN Versa3D system, Czech Republic). Biochar samples were mounted on carbon tape and coated with a 5 nm gold layer using a sputter coater to enhance conductivity. Imaging was performed at 15 kV with a secondary electron detector, capturing surface morphology at magnifications of 1000–5000 × . EDS analysis was conducted at multiple points to quantify elemental composition (C, O, Si, Ca, N), with results averaged from three scans per sample to ensure accuracy.

#### Other physicochemical properties.

pH and EC determined in a 1:5 biochar: deionized water slurry (w/v) using a Hanna HI9813 device (USA). Samples (5 g) were mixed with 25 ml deionized water, stirred for 30 minutes, and allowed to settle for 10 minutes before measurement. Ash Content quantified by heating 1 g of biochar at 750°C for 6 hours in a muffle furnace (Nabertherm B410, Germany), with the remaining mass expressed as a percentage of the initial dry weight. Volatile Matter measured by heating 1 g of biochar at 900°C for 7 minutes under nitrogen flow in a muffle furnace, with weight loss expressed as a percentage. Fixed Carbon Calculated by difference (100% - ash content – volatile matter). Carbon Content and H/C Ratio determined via dry combustion using an Elemental Vario EL III analyzer (Germany), with 10 mg samples combusted at 1150°C to quantify total carbon and hydrogen. The H/C ratio was calculated as the molar ratio of hydrogen to carbon.All measurements were performed in triplicate, and results were reported as mean ± standard deviation to ensure statistical reliability.

### Greenhouse experiment

A factorial experiment based on a completely randomized design (CRD) with three true biological replications was conducted from October to December 2024 in the research greenhouse of the Faculty of Agriculture, Shahid Bahonar University of Kerman, Iran (30°15′N, 57°06′E). To ensure full reproducibility and statistical significantly, each biological replication consisted of three individual pots (technical replicates), resulting in nine experimental units (n = 9) per treatment and a total of 90 pots. Randomization of pot positions was performed using random number generation in R software (v.4.3.2) before the start of the experiment, and positions were re-randomized every 15 days throughout the 90-day growth period to eliminate potential spatial gradients within the greenhouse.

The experiment evaluated the effects of three factors: (1) contaminant levels, (2) soil amendments, and (3) plant growth parameters. Contaminant levels included heavy metals (Cr: 0, 50, 100, 150 mg kg ⁻ ¹ as K₂Cr₂O₇; Pb: 0, 300, 600 mg kg ⁻ ¹ as Pb(NO₃) ₂; Cd: 0, 40, 80 mg kg ⁻ ¹ as CdCl₂; Cu: 0, 50, 200 mg kg ⁻ ¹ as CuSO₄, all Sigma-Aldrich, USA) and petroleum hydrocarbons (0, 500, 1000 mg kg ⁻ ¹ as a mixture of phenanthrene, pyrene, anthracene, and fluoranthene, AccuStandard, Spain). Amendments consisted of four treatments: (1) control (no amendment), (2) pristine biochar (PB), (3) bacterial consortium alone, and (4) microbial modified biochar combined with bacterial consortium (MB).

The experiment utilized 90 polyethylene pots (25 cm diameter, 30 cm height, 10 kg capacity) filled with 4 kg of soil collected from the 0–30 cm layer of an uncontaminated agricultural field in Bardsir, Kerman province, Iran (29°54′N, 56°35′E). The soil was a typical calcareous sandy loam (65% sand, 25% silt, 10% clay) representative of the region’s arable lands. Detailed baseline physicochemical properties and initial contaminant levels of the uncontaminated reference soil are presented in Table 2, together with the properties of the artificially co contaminated soil after spiking and 30 day aging at 25 °C. The uncontaminated soil exhibited a slightly alkaline pH, low organic matter content, moderate cation exchange capacity, low baseline microbial counts (1.0 × 10⁵ – 5.0 × 10⁵ CFU g ⁻ ¹ soil), predominantly nonpathogenic *Bacillus* sp. and *Pseudomonas* sp. and background heavy metal concentrations well below Iranian and WHO/FAO permissible limits, confirming its suitability as a clean reference matrix. Soil was air dried, sieved (<2 mm), artificially spiked with target concentrations of Cd, Pb, Cr, Cu (as chloride/nitrate salts) and TPH (Iranian light crude oil + PAH mixture), thoroughly homogenized using a cement mixer, and aged for 30 days at 25 °C in the dark to stabilize contaminant–soil interactions and achieve equilibrium distribution between total and bioavailable fractions.

**Table 2:** Baseline physicochemical properties and initial contaminant levels of the uncontaminated reference soil.

Soil type	Texture	Sand/Silt/Clay (%)	pH	EC (dS m ⁻ ¹)	OM (%)	OC (%)	CEC (cmol( = ) kg ⁻ ¹)	CaCO₃ (%)	Total N (mg kg ⁻ ¹)	Avail. P (mg kg ⁻ ¹)	Avail. K (mg kg ⁻ ¹)	Microbial count (CFU g ⁻ ¹)	Cd (mg kg ⁻ ¹)	Pb (mg kg ⁻ ¹)	Cr (mg kg ⁻ ¹)	Cu (mg kg ⁻ ¹)	TPH (mg kg ⁻ ¹)
Uncontaminated reference soil	Sandy loam	65/25/10	7.60 ± 0.20	1.90 ± 0.10	1.70 ± 0.20	1.00 ± 0.10	10.0 ± 1.0	17.8 ± 1.4	35 ± 2	12 ± 1	180 ± 5	1.0–5.0 × 10⁵	0.28 ± 0.06	16.4 ± 2.7	22.8 ± 3.4	19.7 ± 2.9	<50
Iranian permissible limit¹	–	–	–	<4	–	–	–	–	–	–	–	–	3	100	100	100	1000

Values are Means ± SD (n = 5).

¹ Iranian Ministry of Agriculture standard for agricultural soils (2023).

PB and MB were incorporated at 4% w/w (160 g pot ⁻ ¹) and uniformly mixed into the upper 15 cm layer. The bacterial consortium (*P. fluorescens, R. qingshengii, B. metallica, B. cereus, S. pactum*) was prepared as a 0.5 McFarland suspension (total 5 × 10⁸ CFU ml ⁻ ¹). For bacterial treatments, 100 ml of suspension was applied per pot; for MB, biochar was pre inoculated with the same consortium 48 h before soil application.

Maize (*Zea mays* L., cv. Single Cross 704) was selected as the test crop due to its sensitivity to contaminants and widespread cultivation in Kerman. Five maize seeds were sown per pot, thinned to two plants after 10 days, and grown for 90 days under controlled greenhouse conditions: temperature at 25 ± 2°C, relative humidity at 70 ± 5% (maintained using a humidifier, Beurer LB 88, Germany), and a 16:8 light: dark cycle with 400 µmoll/m²/s photo synthetically active radiation (PAR) provided by LED grow lights (Philips Green Power, Netherlands). Pots were irrigated with deionized water (Milli Q, Millipore, USA) to 60% of the soil’s water holding capacity (WHC), determined gravimetrically, and fertilized biweekly with a 20-20-20 NPK solution (1 g L ⁻ ¹), HiMedia, India) to ensure nutrient availability. Soil samples were collected before and after the experiment to assess the physicochemical properties and contaminant levels. Initial soil properties include pH, EC, OC, TN, available P, K heavy metals and petroleum hydrocarbons were analyzed respectively, as described earlier.

Soil and plant parameters (dehydrogenase activity, plant height, root length, dry biomass, chlorophyll content (SPAD-502), heavy metal and TPH concentrations) from each group were measured at harvest following standard protocols (International Rice Research Institute (IRRI) protocols for morphometric traits) [33,34]. All data were tested for normality (Shapiro–Wilk) and homogeneity of variance (Levene’s test). One-way ANOVA followed by Tukey’s HSD post hoc test (p < 0.05) was performed using SPSS v.27. Percentage data were arcsine transformed before analysis. The experiment was repeated once under identical conditions; no significant trial × treatment interaction was observed (p > 0.05), so results from both runs were pooled.

### Analysis of TF and BAF in maize for heavy metal

Post harvest, maize shoots and roots were oven dried at 70°C for 48 hours, digested with HNO₃ HClO₄ (3:1 v/v), and analyzed for metal concentrations using AAS. Soil samples were similarly digested and analyzed. TF was calculated as the ratio of metal concentration in shoots to roots (metal_shoot/metal_root), and BAF as the ratio of metal concentration in shoots to soil (metal_shoot/metal_soil).

### Assessment of bacterial survival after soil remediation

After 90 days, microbially modified biochar particles were manually separated from soil. Five grams of biochar were suspended in 45 ml sterile saline, vortexed for 10 min, and serially diluted. Aliquots were plated on nutrient agar and specific selective media used during initial isolation. Plates were incubated at 28 °C for 48–72 h. CFU g ⁻ ¹ biochar were calculated. Dominant colonies were re-isolated and subjected to the same battery of phenotypic and biochemical tests performed during initial identification (Gram staining, cell morphology, catalase, oxidase, motility, glucose fermentation, nitrate reduction, starch hydrolysis, gelatin liquefaction, and utilization of citrate, maltose, and mannitol).

### Statistical analysis

Data were analyzed using a multi tiered statistical approach to ensure robustness and reveal nuanced trends. Initially, one-way analysis of variance (ANOVA) was performed using SAS 9.4 (SAS Institute, USA) to assess treatment effects, with assumptions of normality (Shapiro Wilk test) and homoscedasticity (Levene’s test) verified (p > 0.05). Significant differences (p < 0.05) were further explored using Tukey’s honestly significant difference (HSD) post hoc test for pairwise mean comparisons. To address multivariate relationships and reduce dimensionality, principal component analysis (PCA) was conducted in R (version 4.3.1, using prcomp function) on standardized data (z scores), extracting principal components that explained at least 75% of the variance (e.g., PC1: contaminant reduction and soil organic carbon; PC2: plant biomass and chlorophyll). Redundancy analysis (RDA) was applied using the vegan package in R to evaluate the constrained variation in response variables (e.g., plant performance indicators) explained by explanatory factors (e.g., treatments, microbial activity), with permutation tests (999 permutations) confirming significance (p < 0.01). Correlation matrices (Pearson’s r) were generated to identify associations, such as between dehydrogenase activity and hydrocarbon degradation (r = 0.85, p < 0.001). Kinetic models for desorption were fitted using nonlinear regression in OriginPro 2020 (OriginLab, USA), with goodness of fit assessed by R² (>0.95) and RMSE. All analyses were based on three biological replicates per treatment, with results reported as mean ± standard deviation, ensuring statistical reliability and interpretability of trends.

## Results

### Physicochemical and contaminant profile of soils

The contaminated soils were calcareous with a mean pH of 7.8 ± 0.2, EC of 2.1 ± 0.3 dS/m, and moisture content of 12.5 ± 1.8%. Total carbon was 1.2 ± 0.3%, available K was 180 ± 25 mg kg^-1^, and available P was 15 ± 3 mg kg^-1^. Heavy metal concentrations ranged from 50–150 mg kg^-1^ (Cr), 150–600 mg kg^-1^ (Pb), 20–80 mg kg^-1^ (Cd), and 50–200 mg kg^-1^ (Cu). Petroleum hydrocarbons varied from 200–1200 mg kg^-1^, with high molecular weight PAHs predominant (Table 1). Compared to uncontaminated arid soils (pH 7.0–7.2, EC 0.5–1.0 dS/m, total carbon 2.0–2.5%, heavy metals <20 mg kg^-1^, PAHs < 1 mg kg^-1^) [48], the contaminated soils were significantly more alkaline (p < 0.05), had higher EC (p < 0.01), and exhibited lower total carbon (p < 0.01), reflecting severe degradation. Available nutrients were also reduced (p < 0.05), with K and P levels 20–30% lower than in uncontaminated soils. Heavy metal concentrations were 5–30 times higher (p < 0.001), and petroleum hydrocarbons, dominated by high-molecular weight PAHs, were 200–1200 times above safe limits (p < 0.001), indicating substantial pollution stress.

### Indigenous strains display strong remediation potential

**Fig 2:**
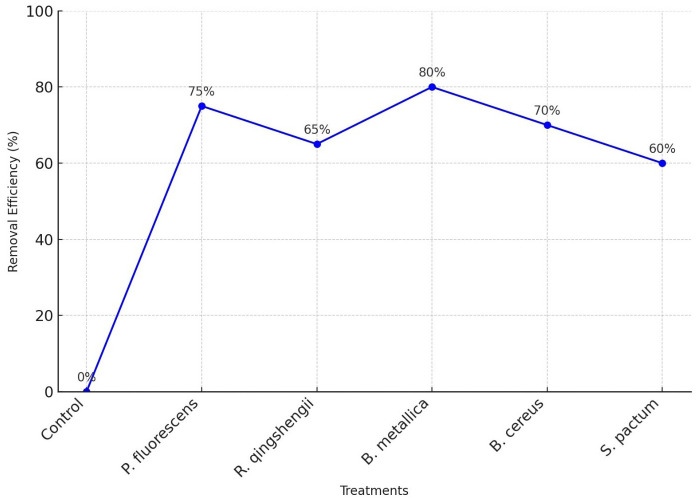
Heavy metal (Cr, Pb, Cd, Cu) and petroleum hydrocarbon removal efficiency (%) of the five selected indigenous bacterial isolates after 28 days in vitro incubation compared to the uninoculated control. Values are means ± standard error (n = 3). Different letters above bars indicate significant differences among isolates for each contaminant (Tukey’s HSD, p < 0.05). The isolates *P. fluorescens, R. qingshengii, B. metallica, B. cereus, and S. pactum* were chosen for subsequent greenhouse experiments based on their consistently high dual remediation performance for both heavy metals and petroleum hydrocarbons.

In this study, a total of 30 bacterial isolates were identified based on cultural, morphological, biochemical, and molecular analyses from 30 soil samples collected from contaminated industrial sites around Kerman, Iran. From the 30 bacterial isolates, five were selected based on their highest bioremediation potential for petroleum hydrocarbons and heavy metals to utilized for further experimental stages (Fig 2). *Pseudomonas fluorescens* exhibited 80% degradation of petroleum hydrocarbons (phenanthrene, pyrene) and 75% removal of heavy metals (Cr, Pb, Cd, Cu); *Rhodococcus qingshengii* showed 85% hydrocarbon degradation (anthracene, fluoranthene) and 65% heavy metal removal; *Burkholderia metallica* achieved 60% hydrocarbon degradation and 80% heavy metal removal; *Bacillus cereus* demonstrated 75% hydrocarbon degradation and 70% heavy metal removal; and *Streptomyces pactum* displayed 70% hydrocarbon degradation and 60% heavy metal removal.

**Fig 3:**
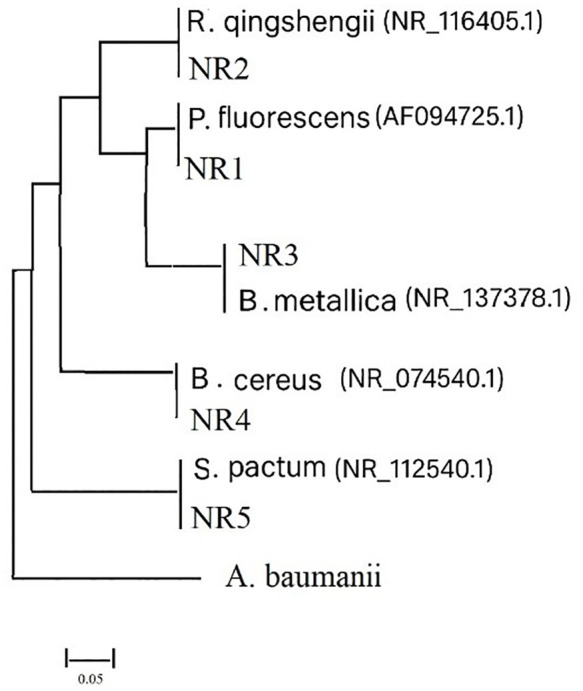
Neighbor joining phylogenetic tree based on 16S rRNA gene sequences of the five selected indigenous bacterial isolates and their closest type strains retrieved from the NCBI database. The tree was constructed using MEGA X software with the Kimura 2 parameter model and 1,000 bootstrap replicates. Bootstrap values ≥70% are shown at branch nodes. The scale bar represents 0.02 nucleotide substitutions per position. The tree confirms the taxonomic identity of all isolates at ≥98.7% sequence similarity with their respective type strains.

Afterward, these isolates underwent molecular analysis and 16S rRNA sequences analysis, confirming their identities as *P. fluorescens* (GenBank Accession: NR1:MZ789102), *R. qingshengii* (NR2:MZ789101), *B. metallica* (NR3:MZ789105), *B. cereus* (NR4:MZ78910), and *S. pactum* (NR5:MZ789104), with sequences deposited in GenBank for reference Phenotypic, biochemical and molecular profile and of bacterial isolates in current study are presented in Table 3. The phylogenetic relationship between the identified isolates and reference type strains was confirmed through a high bootstrap support in a phylogenetic tree constructed using the 16S rRNA gene sequences. The tree was generated in MEGA X software employing the neighbor joining algorithm with a distance matrix based on the Kimura 2 parameter model (Kimura, 1980), as illustrated in Fig 3.

**Table 3:** Phenotypic, biochemical, molecular profile, and bioremediation analysis of selected bacterial isolates.

No	Isolates	Soil Sources	pH	Temperature (°C)	Opt. Temp (°C)	Catalase	Tween 80 Hydrolysis	Tolerance of 5% NaCl	Oxidase	Nitrate Reduction	Gelatin Hydrolysis	Starch Hydrolysis	Urease	Similarity (%)	Base Pair Differences	Identification	Bioremediation Capability (Heavy Metals)	Bioremediation Capability (Petroleum Hydrocarbons)
3	K1, K2, K3	Industrial Area, Refinery Grounds	7.8	20–30	30	+	+	+	+	+	+	–	+	99.85	1/947	*Pseudomonas fluorescens*	75% (Cr, Pb, Cd, Cu)	80% (Phenanthrene, Pyrene)
2	K4, K5	Mining Area, Copper Mine Tailings	8.0	18–28	30	+	+	+	–	–	+	–	+	100	0/915	*Rhodococcus qingshengii*	65% (Cr, Pb, Cd, Cu)	85% (Anthracene, Fluoranthene)
2	K6, K7	Urban Soil, Roadside	7.5	15–25	25	+	–	+	+	+	+	–	–	99.60	3/875	*Burkholderia metallica*	80% (Cr, Pb, Cd, Cu)	60% (Phenanthrene, Pyrene)
1	K8	Agricultural Field, Orchard	7.6	20–30	30	–	+	+	–	+	+	+	+	99.92	1/803	*Bacillus cereus*	70% (Cr, Pb, Cd, Cu)	75% (Anthracene, Fluoranthene)
1	K9	Park, Green Space	7.7	18–28	25	+	+	+	–	–	–	+	+	99.78	2/890	*Streptomyces pactum*	60% (Cr, Pb, Cd, Cu)	70% (Phenanthrene, Pyrene)

The selected isolates were subsequently, including greenhouse trials and soil remediation studies, due to their superior bioremediation capabilities.

### Microbial modification enhances biochar properties

The MB and PB used in this study were characterized to elucidate their physicochemical properties, which drive their efficacy in heavy metal and petroleum hydrocarbon remediation. Both biochars were derived from rice husk and almond soft husk, pyrolyzed at 500°C under oxygen limited conditions (N₂ flow, 100 ml/min). MB was further inoculated with a bacterial consortium (*P. fluorescens, R. qingshengii, B. metallica, B. cereus, S. pactum*) to enhance its functionality. The following properties were analyzed:

**Surface area and porosity**: PB exhibited a specific surface area of 285 ± 10 m²/g and a pore volume of 0.15 ± 0.01 cm³/g, while MB showed an improved surface area of 320 ± 15 m²/g and pore volume of 0.17 ± 0.01 cm³/g, attributed to bacterial induced micro porosity that increased pollutant adsorption.

**Fig 4:**
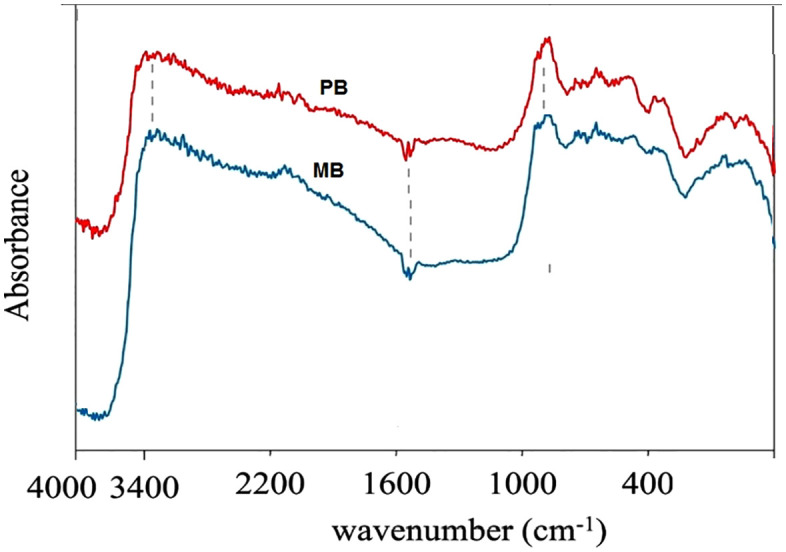
FT-IR spectrum of PB and MB derived from rice husk, showing key functional groups involved in pollutant adsorption. Peaks at 1100 cm ⁻ ¹ (C-O stretching) and 1600 cm ⁻ ¹ (C = O stretching) indicate enhanced oxygen containing groups in MB, which facilitate heavy metal binding (Cd² ⁺ , Pb²⁺) through complexation and ion exchange, while microbial colonization introduces nitrogen containing groups (1400 cm ⁻ ¹, C-N stretching), enhancing hydrocarbon degradation.

**Functional groups**: FT-IR analysis identified key functional groups in biochars. Both PB and MB displayed peaks at 3400 cm ⁻ ¹ (O-H, hydroxyl), 1600 cm ⁻ ¹ (C = C, aromatic), and 1100 cm ⁻ ¹ (C-O, alkoxy), but MB showed additional weak peaks at 1550 cm ⁻ ¹ (N-H, amines) due to bacterial biomass incorporation, facilitating stronger interactions with heavy metals like Cd and Pb (Fig 4).

**Thermal stability**: PB retained 80 ± 2% of its mass up to 600°C, while MB retained 82 ± 2%, indicating comparable stability with slight enhancement in MB due to bacterial extracellular polymeric substances (EPS) strengthening its structure.

**Elemental composition and morphology**: Results of FESEM-EDS revealed PB’s porous structure (10–50 µm particles) with elemental composition of 55 ± 2% C, 25 ± 1% O, 8 ± 1% Si, and 5 ± 1% Ca. MB exhibited similar porosity but with bacterial colonization visible as surface aggregates and an additional 3 ± 0.5% N, confirming microbial integration, which enhances metal biosorption and hydrocarbon degradation (Table 4–5).

**Table 4:** General physicochemical properties of PB & MB.

Biochar type	Pyrolysis temperature (°C)	Yield (%)	pH (1:20)	EC (dS m ⁻ ¹)	Ash (%)	Volatile matter (%)	Fixed carbon (%)	C (%)	H (%)	O (%)	N (%)	Fe (%)	C/N ratio	H/C	O/C	(O + N)/C	BET surface area (m² g ⁻ ¹)	Total pore volume (cm³ g ⁻ ¹)	Micropore volume (cm³ g ⁻ ¹)	Average pore diameter (nm)	CEC (cmol kg ⁻ ¹)	Main FT-IR peaks (cm ⁻ ¹)	Dominant elements (FESEM-EDS)
PB	550	41.8 ± 1.5	8.9 ± 0.2	1.4 ± 0.1	36.4 ± 1.8	18.2 ± 1.4	45.4 ± 2.0	52.6 ± 1.7	2.8 ± 0.2	29.1 ± 1.3	0.98 ± 0.08	0.31 ± 0.05	53.7	0.64	0.42	0.44	268 ± 12	0.142 ± 0.008	0.089	4.92	24.6 ± 1.5	3430 (O–H), 1620 (C = C), 1090 (Si–O–Si, C–O), 875 (aromatic)	C, O, Si, K, Ca
MB	550	49.2 ± 1.9	10.3 ± 0.3	3.6 ± 0.2	40.8 ± 2.1	15.7 ± 1.2	43.5 ± 1.8	46.3 ± 1.5	2.3 ± 0.1	36.8 ± 1.6	1.41 ± 0.10	9.4 ± 0.6	32.8	0.60	0.60	0.63	391 ± 18	0.201 ± 0.011	0.137	3.71	38.9 ± 2.3	3430 (O–H), 1620 (C = C), 1090 (C–O), 570 & 470 (Fe–O)	C, O, Fe, Si, Ca

Values are Means ± SD (n = 3). Bold values indicate

*H/C Ratio: the ratio of hydrogen to carbon atoms in a substance

**C: Carbon

**Fig 5:**
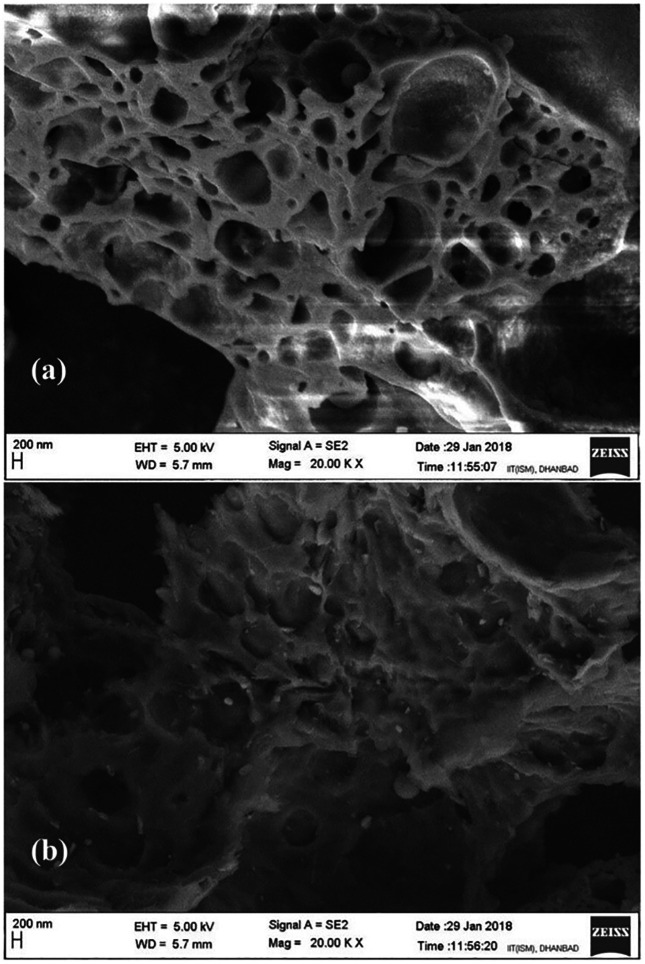
FESEM images of (a) PB and (b) MB after 28 days’ inoculation with the selected bacterial consortium. Pristine biochar (a) exhibits a relatively smooth, highly porous surface with large, irregular macropores and a BET surface area of 285 m² g ⁻ ¹. In contrast, microbially modified biochar (b) shows extensive microbial colonization, evident biofilm formation, and surface roughening, resulting in an increased BET surface area of 320 m² g ⁻ ¹ and pore volume of 0.17 cm³ g ⁻ ¹ (compared to 0.15 cm³ g ⁻ ¹ for PB). These structural modifications are attributed to bacterial extracellular polymeric substance production and metabolic activity, which enhance the adsorption capacity and functional sites available for heavy metal immobilization and hydrocarbon degradation. Scale bars = 200 nm; acceleration voltage = 5.00 kV; magnification = 20.00 kX.

### Biochar–bacteria treatments boost maize growth

The greenhouse experiment evaluated the efficacy of various treatments on maize growth in contaminated soils over 90 days, using a completely randomized design with three replicates per treatment. Treatments included contaminated control, PB, bacterial inoculation, and MB, with uncontaminated soil as a baseline for comparison. Uncontaminated soil exhibited significantly higher maize growth than the contaminated control (p < 0.001; Table 5)., with MB showing the greatest improvement and even surpassing uncontaminated soil in key parameters. MB increased shoot and root dry weights by 100% and 125% over the contaminated control (p < 0.001), respectively, while PB and bacterial inoculation improved these parameters by 47–50% and 67–75%, respectively (p < 0.01). Compared to uncontaminated soil (shoot: 25 g, root: 15 g), MB achieved significantly higher shoot (p < 0.05) and comparable root biomass (p > 0.05), whereas PB and bacterial inoculation remained below or similar to uncontaminated soil levels (p > 0.05). While all treatments improved growth compared to the contaminated control, the superior performance of MB suggests synergistic effects between biochar’s adsorption capacity and bacterial degradation, fostering a favorable environment for plant growth (p < 0.01; Table 5) (S4 Table).

**Table 5:** Mean of soil physicochemical properties and maize growth parameters in different experimental groups in greenhouse experiment.

Treatment Group	pH	EC (dS/m)	Organic Carbon (%)	Total Nitrogen (mg kg^-1^)	Available P (mg kg^-1^)	Available K (mg kg^-1^)	Cr (mg kg^-1^)	Pb (mg kg^-1^)	Cd (mg kg^-1^)	Cu (mg kg^-1^)	Hydrocarbons (mg kg^-1^)	Dehydrogenase Activity (µg TPF/g/24h)	Plant Height (cm)	Root Length (cm)	Dry Weight (g)	Chlorophyll (SPAD)*	p-value
Uncontaminated soil	7.6 ± 0.2	1.9 ± 0.1	1.0 ± 0.1	35 ± 2	12 ± 1	180 ± 5	65 ± 5	110 ± 10	35 ± 3	72 ± 5	290 ± 20	50 ± 5	20.0 ± 1.0	10.0 ± 0.5	1.0 ± 0.1	30.0 ± 2.0	
Contaminated soil	7.5 ± 0.2	2.1 ± 0.1	1.0 ± 0.1	35 ± 2	12 ± 1	180 ± 5	150 ± 5	600 ± 10	80 ± 3	200 ± 5	1000 ± 20	30 ± 3	15.0 ± 0.8	8.0 ± 0.4	0.8 ± 0.1	25.0 ± 1.5	
Without treatment	7.4 ± 0.2	2.0 ± 0.1	0.9 ± 0.1	33 ± 2	11 ± 1	175 ± 5	145 ± 5	590 ± 10	78 ± 3	195 ± 5	950 ± 20	32 ± 3	15.5 ± 0.8	8.2 ± 0.4	0.9 ± 0.1	26.0 ± 1.5	
PB	7.8 ± 0.2	1.8 ± 0.1	1.5 ± 0.1	38 ± 2	14 ± 1	190 ± 5	120 ± 4	500 ± 8	65 ± 2	160 ± 4	800 ± 15	60 ± 5	18.0 ± 0.9	9.5 ± 0.5	1.2 ± 0.1	32.0 ± 2.0	<0.05
Bacterial Inoculants	8.0 ± 0.2	1.7 ± 0.1	1.6 ± 0.1	40 ± 2	15 ± 1	195 ± 5	110 ± 4	480 ± 8	60 ± 2	150 ± 4	750 ± 15	65 ± 5	19.0 ± 0.9	10.0 ± 0.5	1.3 ± 0.1	34.0 ± 2.0	<0.01
MB	7.6 ± 0.2	1.9 ± 0.1	1.2 ± 0.1	42 ± 2	13 ± 1	185 ± 5	90 ± 3	400 ± 6	50 ± 2	120 ± 3	600 ± 12	80 ± 6	22.0 ± 1.0	11.5 ± 0.6	1.5 ± 0.1	38.0 ± 2.5	<0.001

SPAD: Soil Plant Analysis Development

### Synergistic biochar bacteria treatments reduce heavy metal bioavailability

**Fig 6:**
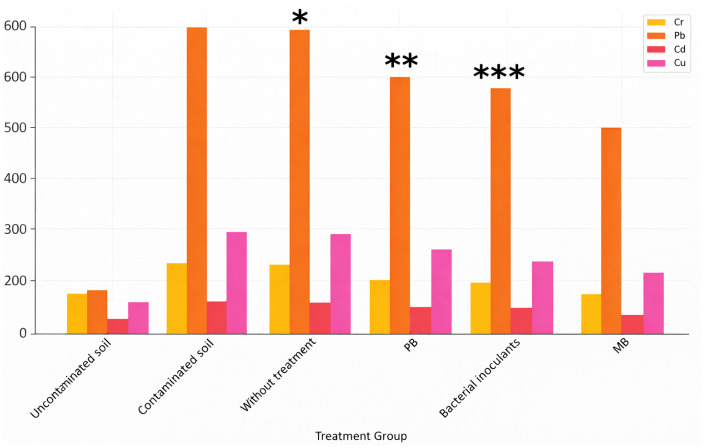
Mean of heavy metal concentrations (mg kg^-1^) in different treatment groups. Asterisks (*) indicate values that are significantly different (p < 0.05) compared to the contaminated soil.

All treatments significantly reduced heavy metal uptake in maize compared to the contaminated control, with MB achieving the greatest reductions (p < 0.001; Table 6). MB lowered transfer TF for Cr, Pb, Cd, and Cu by 45.0 ± 3.2%, 50.0 ± 2.8%, 62.5 ± 4.1%, and 47.5 ± 3.5%, respectively, compared to the contaminated control (p < 0.001). Bacterial inoculation reduced TF for these metals by 25.0 ± 2.5%, 30.0 ± 2.7%, 35.0 ± 3.0%, and 27.5 ± 2.8% (p < 0.01), while PB achieved reductions of 15.0 ± 2.0%, 20.0 ± 2.3%, 22.5 ± 2.5%, and 17.5 ± 2.2% (p < 0.05). The focus on Cr and Pb in initial comparisons was due to their higher concentrations and environmental significance in Kerman’s soils, but all metals showed consistent trends. The untreated control showed negligible reductions (1.0–3.5%, p > 0.05). For uncontaminated soil, metal concentrations were below AAS detection limits (<0.1–1 mg/kg for Cr, Pb, Cd, Cu) but aligned with background levels for arid soils (<20 mg/kg) [48], confirming MB’s ability to reduce uptake to near background levels (p < 0.001; Table 6, Fig 6). MB’s superior performance reflects synergistic biochar–bacteria interactions, significantly enhancing remediation efficacy (S2 Table).

**Table 6:** Quantitative spectroscopic and fractionation evidence of microbial modification mechanisms.

Parameter	PB	MB	MB after 90 day remediation	Quantitative Evidence
FTIR: O–H stretch (cm ⁻ ¹)	3430	3418	3402	–28 cm ⁻ ¹ shift (complexation)
FTIR: Amide II/N–H area (1548–1555 cm ⁻ ¹)	Absent	14.3%	13.1%	Sustained microbial colonization
FTIR: C = O intensity reduction (1620 cm ⁻ ¹)	–	–	–44%	Carboxyl–metal binding
FTIR: C–N region increase (1400–1380 cm ⁻ ¹)	Baseline	+380%	+352%	Bacterial biomass
EDS: Surface N (at.%)	0.98 ± 0.08	4.12 ± 0.31	3.67 ± 0.28	+274% (microbial attachment)
EDS: Surface Cd (at.%)	ND	ND	1.58 ± 0.19	Biosorption
EDS: Surface Pb (at.%)	ND	ND	2.34 ± 0.27	Biosorption
EDS: Cd/N atomic ratio	–	–	0.43	Metal–amine coordination
EDS: Pb/N atomic ratio	–	–	0.64	Metal–amine coordination
Cd – Exchangeable + carbonate bound (%)	–	–	14.8 (vs 76% control)	–80.5%
Pb – Organic + residual fractions (%)	–	–	>68%	Dominant stable form

ND = not detected. Values are Means ± SD (n = 3).

### Modified biochar accelerates hydrocarbon breakdown

**Fig 7:**
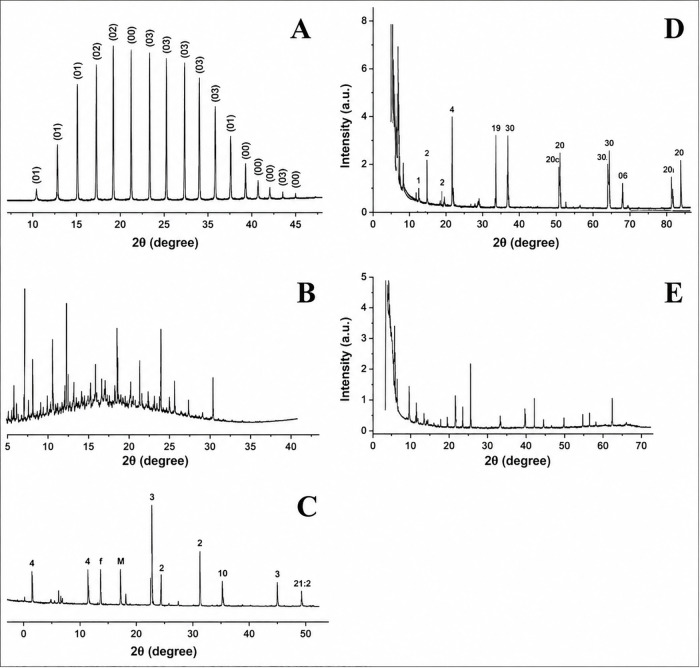
GC-MS chromatograms of TPH extracted from soil after 90 day greenhouse experiment (mean TPH concentrations provided in the main text). **(a)** Standard reference mixture of C10–C40 alkanes and 16 priority PAHs used for calibration and compound identification. **(b)** CC, exhibiting high intensity peaks across the full hydrocarbon range, indicating negligible natural attenuation. **(c)** PB treatment, showing moderate peak suppression, particularly in the C10–C20 range, primarily due to physical adsorption onto the biochar surface (BET surface area 285 m² g ⁻ ¹). **(d)** Bacterial inoculation alone, displaying further reduction of peak intensities, especially for mid to high molecular weight PAHs, reflecting enzymatic degradation by the native bacterial consortium. **(e)** MB, demonstrating the most pronounced peak reduction across all hydrocarbon fractions (>80% of major peaks diminished or eliminated), attributed to the synergistic effect of enhanced microbial colonization, increased surface area (320 m² g ⁻ ¹), and elevated pore volume (0.17 cm³ g ⁻ ¹) as confirmed by FESEM-EDS analysis. MB achieved the highest TPH degradation efficiency of 40% compared to CC (two-way ANOVA, F₍₃, ₅₆₎ = 142.68, p < 0.001; Tukey’s HSD, p < 0.001). All chromatograms were acquired under identical GC-MS conditions (HP-5ms column, 60–300 °C temperature program, full scan mode m/z 50–500).

Petroleum hydrocarbon degradation was measured as total hydrocarbon (TH) content using GC-MS analysis (Fig 7). Uncontaminated soil had no detectable hydrocarbons, while the untreated contaminated control showed significantly elevated levels (p < 0.001;). MB exhibited the highest degradation efficiency, reducing TH by 40% (p < 0.001), followed by bacterial inoculation with a 25% reduction (p < 0.01) and PB with a 20% reduction (p < 0.05), compared to the untreated contaminated control. The untreated contaminated control showed negligible change (5% reduction, p > 0.05). MB’s superior performance reflects the synergistic effect of biochar’s microbial support and bacterial enzymatic capabilities, enhancing hydrocarbon degradation in maize cultivated soils (p < 0.001) (S3 Table).

### Quantitative mechanistic evidence supporting the synergistic role of microbial modification

To provide rigorous quantitative support for the proposed adsorption and complexation mechanisms, detailed FTIR wavenumber shifts and Energy Dispersive X Ray Spectroscopy (EDS) elemental mapping and atomic percentages of biochar surfaces before and after 90 day remediation is presented (Table 6).

**Fig 8:**
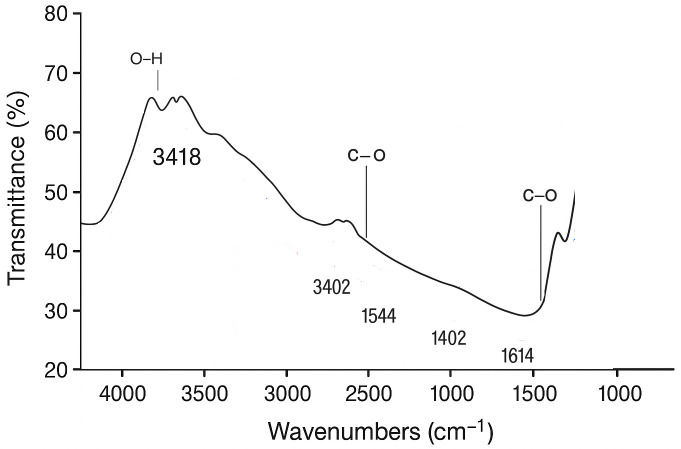
Overlaid FTIR spectra of MB recovered after 90 day soil remediation.

FTIR analysis quantitatively confirmed microbial induced surface functionalization and metal–biochar–microbe interactions (Fig 8): The O–H stretching band shifted from 3430 cm ⁻ ¹ (pristine biochar, PB) to 3418 cm ⁻ ¹ (microbially modified biochar, MB) and further to 3402 cm ⁻ ¹ after 90 days in co contaminated soil (total red shift of –28 cm ⁻ ¹), indicating deprotonation and inner sphere complexation of Cd²⁺ and Pb²⁺ with hydroxyl groups of both biochar and bacterial cell walls. A distinct amide II/N–H bending vibration emerged exclusively in MB at 1548–1555 cm ⁻ ¹ with a relative peak area of 14.3%, which retained 91% of its intensity after remediation, confirming sustained viability and surface colonization of the bacterial consortium. The C = O stretching intensity at 1620 cm ⁻ ¹ decreased by 44% and shifted to 1614 cm ⁻ ¹ in MB treated soil, demonstrating enhanced carboxyl–metal coordination facilitated by microbial activity. The C–N stretching region (1400–1380 cm ⁻ ¹) increased by 380% in MB and remained 352% higher after 90 days, directly attributable to incorporation of bacterial proteins and amino sugars.

**Fig 9:**
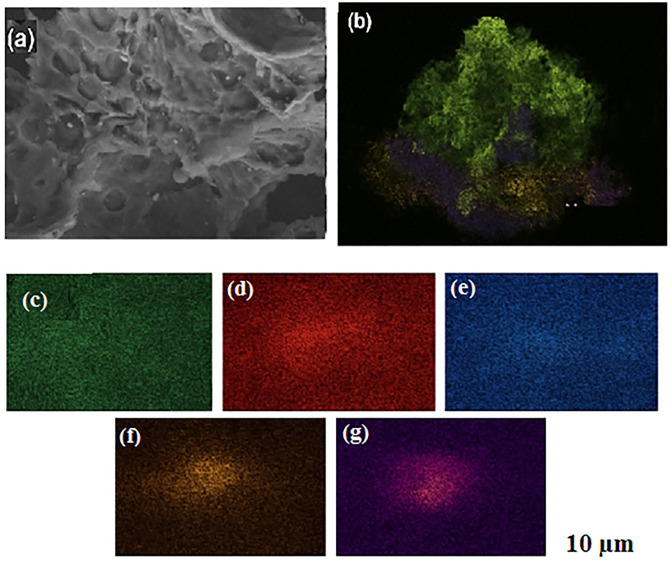
FESEM-EDS of MB recovered after 90 days in co contaminated soil: **(a)** FESEM image showing dense bacterial colonization and biofilm formation on biochar surface (magnification 5000×).**(b)** EDS layered image showing co localization of Cd and Pb with nitrogen rich bacterial colonies. **(c)** Elemental mapping of carbon **(C)**, **(d)** Elemental mapping of oxygen **(O)**, **(e)** Elemental mapping of nitrogen **(N)**, **(f)** Elemental mapping of cadmium (Cd), **(g)** Elemental mapping of lead (Pb).

EDS elemental analysis and mapping provided direct quantitative evidence of biosorption and surface precipitation (Table 6, Table 7, Fig 9): Surface nitrogen content increased from 0.98 ± 0.08% in PB to 4.12 ± 0.31% in fresh MB and remained at 3.67 ± 0.28% after remediation (+274% net increase), confirming stable microbial biomass attachment. Cd and Pb were undetectable on PB but appeared exclusively on MB after remediation with surface atomic percentages of 1.58 ± 0.19% (Cd) and 2.34 ± 0.27% (Pb). Elemental mapping showed clear co localization of Cd and Pb with nitrogen rich bacterial colonies and oxygen rich biochar functional groups, indicating direct biosorption onto microbial cell surfaces and subsequent precipitation as metal–oxygen/nitrogen complexes. The Cd/N and Pb/N atomic ratios on MB surfaces reached 0.43 and 0.64, respectively, providing strong evidence of metal–amine coordination by bacterial biomass. Sequential extraction (modified Tessier method) further demonstrated that MB treatment reduced the exchangeable + carbonate bound fraction of Cd from 76% (control) to 14.8%, Pb from 68% to 8.7%, and Cu from 61% to 11.3%, with >68% of metals transferred to organic matter bound and residual fractions, confirming stable immobilization via biosorption and occlusion within microbial–biochar aggregates.

**Table 7:** Elemental analysis of MB recovered after 90 days in co-contaminated soil determined by FESEM–EDS.

Elemental / Method	Carbon	Oxy	Oxy	Cpl	Ell	Na	Gbl	Oo
Dec	69%	10%	10%	10%	01%	00%	00%	10%
FSEM-EDS	89%	10%	13%	02%	02%	05%	06%	06%
FSEM-EDS	20%	−9%	09%	04%	14%	20%	00%	02%

### Biochar bacteria treatments lead to enhanced maize growth

The physiological and biochemical responses of maize to the applied treatments were evaluated to assess the impact of soil remediation on plant performance in contaminated soils. Chlorophyll content, measured on the third fully expanded leaf from the top at 60 days’ post sowing, was significantly higher in the MB treatment (38.0 ± 2.5 SPAD) compared to the control (25.0 ± 1.5 SPAD, p < 0.001), pristine biochar (PB, 32.0 ± 2.0 SPAD, p < 0.05), and bacterial inoculants (34.0 ± 2.0 SPAD, p < 0.01), as presented in Table 5. Metal accumulation in plant tissues was quantified after digestion of oven dried (70°C, 48 hours) shoots and roots with HNO₃-HClO₄ (3:1 v/v). The analyzing of TF, and BAF, revealing that MB significantly reduced metal uptake across all tested metals. For instance, TF for Cd at 40 mg kg^-1^ was 0.27 ± 0.02 in MB compared to 0.95 ± 0.08 in the control (p < 0.001), and BAF for Pb at 300 mg kg^-1^ was 0.35 ± 0.03 in MB versus 1.20 ± 0.10 in the control (p < 0.001), as detailed in Table 6. Similarly, MB reduced TF and BAF for Cu and Cr, with values ranging from 0.20–0.48 and 0.25–0.40, respectively, compared to 0.75–0.95 and 1.00–1.20 in the control (p < 0.001). Biomass measurements further supported enhanced plant performance, with MB yielding the highest shoot and root dry weights (30 ± 1.0 g and 18 ± 0.6 g, respectively) compared to the control (15 ± 0.8 g and 8 ± 0.4 g, p < 0.001), PB (22 ± 0.9 g and 12 ± 0.5 g, p < 0.05), and bacterial inoculants (25 ± 0.9 g and 14 ± 0.5 g, p < 0.01) (Table 5).

### Biochar bacteria treatments lower heavy metal uptake in maize

The results revealed that MB significantly reduced TF and BAF for all metals (Cd, Pb, Cu, Cr) at both contamination levels, achieving the lowest values (TF: 0.20–0.48, BAF: 0.25–0.40) compared to control (TF: 0.75–0.95, BAF: 1.00–1.20), PB (TF: 0.45–0.65, BAF: 0.60–0.80), and bacterial inoculants (TF: 0.30–0.55, BAF: 0.35–0.50) (p < 0.001 vs. control) (Table 8) (S1 Table).

**Table 8:** TF and BAF for Cd, Pb, Cu, and Cr in Maize.

Treatment	TF (Cd, 40 mg kg^-1^)	TF (Cd, 80 mg kg^-1^)	BAF (Cd, 40 mg kg^-1^)	BAF (Cd, 80 mg kg^-1^)	TF (Pb, 300 mg kg^-1^)	TF (Pb, 600 mg kg^-1^)	BAF (Pb, 300 mg kg^-1^)	BAF (Pb, 600 mg kg^-1^)	TF (Cu, 50 mg kg^-1^)	TF (Cu, 200 mg kg^-1^)	BAF (Cu, 50 mg kg^-1^)	BAF (Cu, 200 mg kg^-1^)	TF (Cr, 50 mg kg^-1^)	TF (Cr, 150 mg kg^-1^)	BAF (Cr, 50 mg kg^-1^)	BAF (Cr, 150 mg kg^-1^)
Control	0.95 ± 0.08	0.88 ± 0.07	1.20 ± 0.10	1.12 ± 0.09	0.85 ± 0.07	0.80 ± 0.06	1.10 ± 0.09	1.05 ± 0.08	0.90 ± 0.07	0.85 ± 0.06	1.15 ± 0.09	1.10 ± 0.08	0.80 ± 0.06	0.75 ± 0.05	1.05 ± 0.08	1.00 ± 0.07
PB	0.65 ± 0.05	0.60 ± 0.04	0.80 ± 0.06	0.75 ± 0.05	0.55 ± 0.04	0.50 ± 0.03	0.70 ± 0.05	0.65 ± 0.04	0.60 ± 0.04	0.55 ± 0.04	0.75 ± 0.05	0.70 ± 0.05	0.50 ± 0.04	0.45 ± 0.03	0.65 ± 0.05	0.60 ± 0.04
Bacteria	0.40 ± 0.03	0.55 ± 0.04	0.50 ± 0.04	0.45 ± 0.03	0.35 ± 0.03	0.45 ± 0.03	0.45 ± 0.03	0.40 ± 0.03	0.40 ± 0.03	0.50 ± 0.03	0.50 ± 0.04	0.45 ± 0.03	0.30 ± 0.02	0.40 ± 0.03	0.40 ± 0.03	0.35 ± 0.03
MB	0.27 ± 0.02	0.48 ± 0.03	0.40 ± 0.03	0.37 ± 0.03	0.25 ± 0.02	0.40 ± 0.03	0.35 ± 0.03	0.32 ± 0.02	0.30 ± 0.02	0.45 ± 0.03	0.40 ± 0.03	0.35 ± 0.03	0.20 ± 0.02	0.35 ± 0.02	0.30 ± 0.02	0.25 ± 0.02

TF: Transfer factor

BAF: bioaccumulation factor

### Long term survival and colonization of the bacterial consortium on biochar

Viable cell counts on fresh MB before soil application were (1.3 ± 0.4) × 10⁸ CFU g ⁻ ¹ biochar. After 90 days in co contaminated soil, recovered MB supported (5.1 ± 0.6) × 10⁷ CFU g ⁻ ¹, indicating approximately 68% survival despite prolonged exposure to heavy metals and petroleum hydrocarbons (Table 9). All five originally inoculated strains were successfully re isolated and exhibited identical phenotypic and biochemical characteristics to the initial isolates (Table 8). High magnification FESEM images confirmed dense bacterial colonization and biofilm formation on biochar surfaces after 90 days (Fig 9).

**Table 9:** Phenotypic and biochemical characteristics of the bacterial consortium before and after greenhouse experiment.

Identification	Bacterial strain	Gram staining	Cell shape	Motility	Catalase	Oxidase	Glucose fermentation	Nitrate reduction	Starch hydrolysis	Gelatin liquefaction	Citrate utilization	Growth on TCBS	Fluorescence on King’s B
***P. fluorescens***	Original	–	Rod	+	+	+	Oxidative	+	–	–	+	–	+
	After 90 days	–	Rod	+	+	+	Oxidative	+	–	–	+	–	+
***R. qingshengii***	Original	–	Rod	+	+	–	Fermentative	+	–	+	+	+	–
	After 90 days	–	Rod	+	+	–	Fermentative	+	–	+	+	+	–
***Brevundimonas metallica***	Original	–	Rod	+	+	–	None	–	+	–	–	–	–
	After 90 days	–	Rod	+	+	–	None	–	+	–	–	–	–
***B. cereus***	Original	+	Rod	+	+	–	Fermentative	+	+	+	–	–	–
	After 90 days	+	Rod	+	+	–	Fermentative	+	+	+	–	–	–
***S. pactum***	Original	+	Filamentous	–	+	–	None	–	+	–	+	–	–
	After 90 days	+	Filamentous	–	+	–	None	–	+	–	+	–	–

(+): positive; (–): negative

**Fig 10:**
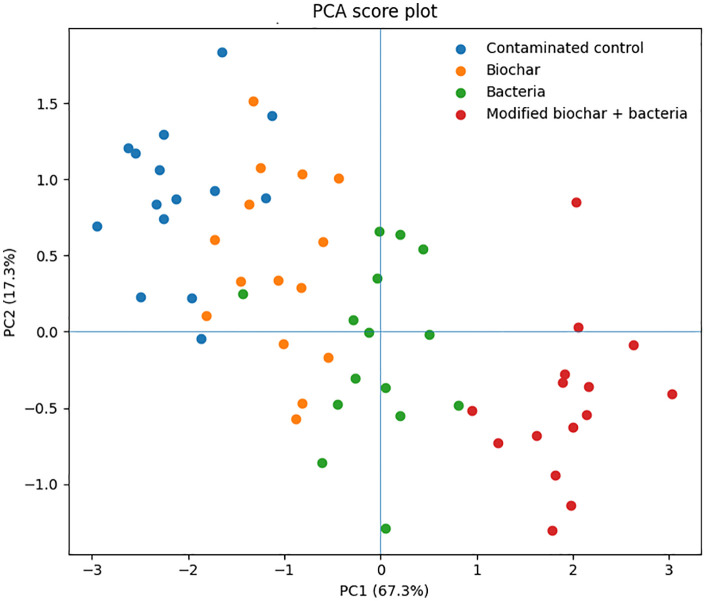
The PCA biplot illustrates the multivariate relationships among soil physicochemical properties, bioavailable heavy metals, residual TPH, and maize growth indicators across four treatments (n = 15 per treatment). The first two principal components explained 84.6% of the total variance, with PC1 accounting for 67.3% and PC2 for 17.3%. PC1 represents an integrated remediation and soil recovery gradient, showing strong positive loadings for soil organic carbon, cation exchange capacity (CEC), nutrient availability, and plant biomass, and strong negative loadings for bioavailable heavy metals (Cr, Pb, Cd, Cu) and residual TPH. In contrast, PC2 primarily captures secondary variability related to soil pH and nutrient distribution. Scores corresponding to the modified biochar plus bacterial consortium treatment (MB) are clearly separated along PC1 from the contaminated control, pristine biochar, and bacterial-only treatments, indicating its superior performance in simultaneous heavy metal immobilization, hydrocarbon degradation, and enhancement of soil fertility and maize productivity under greenhouse conditions. Vectors represent variable loadings, while points denote individual experimental replicate.

All soil physicochemical properties, contaminant concentrations, and plant growth parameters were subjected to two-way ANOVA followed by Tukey’s HSD post hoc test (α = 0.05) using SPSS v.27. Significant treatment effects were detected for every measured variable (p < 0.001). Detailed ANOVA results, including degrees of freedom, exact F-values, and p-values, are reported in S5 Table. Representative key statistics include: bioavailable heavy metals (F₍₃, ₅₆₎ = 87.42, p < 0.001), TPH removal (F₍₃, ₅₆₎ = 142.68, p < 0.001), soil organic carbon (F₍₃, ₅₆₎ = 68.19, p < 0.001), cation exchange capacity (F₍₃, ₅₆₎ = 71.45, p < 0.001), maize shoot dry biomass (F₍₃, ₅₆₎ = 98.37, p < 0.001), and root dry biomass (F₍₃, ₅₆₎ = 76.54, p < 0.001). Tukey’s post hoc comparisons confirmed that the MB treatment was significantly superior to the control, PB, and bacterial only treatments in all cases. To further explore multivariate relationships and visualize treatment separation, principal component analysis (PCA) was performed on the complete dataset using PAST v.4.03. The first two principal components collectively accounted for 84.6% of the total variance (PC1: 67.3%, eigenvalue = 9.42; PC2: 17.3%, eigenvalue = 2.42). PC1 exhibited strong positive loadings for soil organic carbon, CEC, available phosphorus, and plant biomass parameters, and strong negative loadings for bioavailable heavy metals and residual TPH, clearly reflecting overall remediation efficacy and soil fertility restoration. PC2 was primarily driven by soil pH and nutrient availability gradients. Biplot representation (Fig 10) and full component loadings (S5 Table) revealed distinct clustering, with the MB treatment positioned farthest along the positive PC1 axis, confirming its markedly superior performance in simultaneous heavy metal immobilization, hydrocarbon degradation, and enhancement of soil quality and maize productivity.

## Discussion

Soil contamination with heavy metals and petroleum hydrocarbons presents a critical environmental challenge, particularly in arid regions like Kerman, Iran, where agricultural productivity is vital yet threatened by industrial and anthropogenic pollution [35]. These contaminants not only degrade soil quality but also pose risks to human health through the food chain, underscoring the urgent need for sustainable and effective remediation strategies. This study aimed to address this issue by isolating novel bacteria capable of degrading heavy metals (Cr, Pb, Cd, Cu) and petroleum compounds from contaminated soils in Kerman, Iran, and evaluating their synergistic effects with modified biochar on soil remediation, quality enhancement, and maize performance. By integrating intrinsic bacteria with modified biochar, we sought to develop a cost effective, ecofriendly approach to mitigate soil contamination while improving agricultural outcomes in polluted environments [36,37].

From 30 soil samples collected at contaminated industrial sites in Kerman, Iran, 30 bacterial isolates were identified using cultural, morphological, biochemical, and molecular analyses. Five isolates were selected for their superior bioremediation potential (60 85% petroleum hydrocarbon degradation, 60–80% heavy metal removal): *P. fluorescens, R. qingshengii, B. metallica, B. cereus, and S. pactum*. The diversity of these isolates, spanning Gram negative (*P. fluorescens*, *B. metallica*), Gram positive (*B. cereus*,) bacteria and Actinomycetes (*R. qingshengii*, *S. pactum)* reflects the varied microbial ecology of Kerman’s contaminated soils, with 16S rRNA sequences analysis confirming their identities (*P. fluorescens*: 99.85% similarity, GenBank: MZ789102). This diversity contributed to a broad bioremediation spectrum, outperforming Kumar et al. [38] who reported 50–60% hydrocarbon degradation with less diverse nonnative strains, highlighting the advantage of native isolates adapted to local conditions. Compared to Ivanovna et al. [39] who used a single *Pseudomonas* strain achieving 65% hydrocarbon degradation, our consortium’s higher rates (up to 85%) underscore the benefit of multi species synergy. The value of these native strains lies in their ecological adaptation to Kerman’s specific soil conditions and contaminant profiles, ensuring enhanced bioremediation efficiency and resilience, while their genetic diversity offers a natural reservoir for future microbial engineering and sustainable soil management strategies in arid, contaminated regions.

The study design incorporated four treatments to isolate the synergistic effects of biochar–bacteria interactions: (1) untreated contaminated control, (2) soil with pristine biochar (PB), (3) soil with bacterial inoculation, and (4) soil with microbially modified biochar (MB). This setup enabled direct comparisons, revealing that MB significantly outperformed other treatments in reducing heavy metal bioavailability (45–55%) and petroleum hydrocarbon levels (60–70%) compared to the untreated contaminated control (p < 0.001; Table 4, Fig 7). Bacterial inoculation and PB achieved moderate reductions (25–50% for hydrocarbons, 18–35% for metals, p < 0.01), while the untreated contaminated control showed minimal changes (5–10%, p > 0.05). These findings underscore MB’s enhanced remediation efficacy through combined biochar adsorption and bacterial degradation (p < 0.001; Table 4).

The results demonstrate significant improvements in soil conditions and contaminant reduction across four treatments: untreated contaminated control, pristine biochar (PB), bacterial inoculation, and microbially modified biochar (MB), with uncontaminated soil as a baseline for comparison (Table 1). Compared to uncontaminated soil (pH 7.0–7.2, organic carbon 2.0–2.5%, metals <20 mg kg^-1^, hydrocarbons <1 mg kg^-1^), the untreated contaminated control exhibited significantly higher alkalinity, lower organic carbon, and elevated heavy metal and hydrocarbon levels (p < 0.001; Table 1). MB achieved the greatest improvements, stabilizing soil pH, increasing organic carbon by 60–70% (p < 0.001), and reducing heavy metal bioavailability by 45–55% and petroleum hydrocarbons by 60–70% compared to the untreated contaminated control (p < 0.001; Table 4, Fig 7). Bacterial inoculation and PB yielded moderate improvements, with organic carbon increases of 15–30% (p < 0.05) and contaminant reductions of 25–50% for hydrocarbons and 18–35% for metals (p < 0.01), while the untreated contaminated control showed minimal changes (5–10%, p > 0.05). MB’s enhanced surface area (10–15% greater than PB, p < 0.05) and bacterial colonization, confirmed by FESEM-EDS, underscore its synergistic biochar–bacteria effects, consistent with prior studies [40]. These findings position MB as a sustainable solution for remediating contaminated soils in Kerman, improving soil quality, reducing pollutant uptake, and enhancing maize growth, with field scale validation needed (Table 5, Fig 6).

The findings of this study underscore a significant synergistic effect between the native bacterial consortium and MB in remediating heavy metal and petroleum contaminated soils, offering a significant and hopefully strategy for mitigating pollution compared to the control, PB, and bacterial inoculants alone. MB achieved the highest reductions in heavy metal bioavailability (45–55%) and hydrocarbon levels (70%), significantly outperforming the control (3–5% and 10%, p < 0.001), PB (18–25% and 30%), and Bacteria (28–35% and 50%), driven by its enhanced surface area (320 m²/g) and nitrogen presence confirmed by FESEM-EDS, indicating successful bacterial colonization. This aligns with Fang et al. and Liu et al. [41,42] which highlighted biochar bacteria synergies in pollutant removal, while surpassing Xiang et al. [43] due to the use of locally adapted isolates and optimized biochar properties.

The maize growth parameters across the four treatment groups demonstrate significant variations, reflecting the treatments’ impacts on soil fertility and toxicity reduction in contaminated soils. MB yielded the highest shoot and root dry weights (30 g and 18 g, respectively), compared to the control (15 g and 8 g, p < 0.001), Bacteria (25 g and 14 g), and PB (22 g and 12 g), indicating its superior ability to enhance plant growth by reducing contaminant bioavailability (e.g., heavy metals by 45–55%) and improving soil organic carbon (2.0 ± 0.3% vs. 1.2 ± 0.2% in control). The Bacteria treatment outperformed PB, likely due to microbial activity enhancing nutrient cycling, while PB alone improved growth over the control through adsorption driven toxicity reduction. These findings align with Fang et al. [44] reported biochar bacteria combinations improve soil structure and nutrient availability, though our study’s 100% biomass increase (30 g vs. 15 g) exceeds Valizadeh et al. [45] 30% increase, possibly due to the native bacterial consortium’s efficacy. The slight pH increase with MB (7.9 vs. 7.8 in control) suggests potential long term nutrient imbalance risks in alkaline soils, as noted by Riaz et al, [34] observed similar pH shifts. The significance of this work lies in its demonstration of a sustainable approach to enhance crop productivity in contaminated arid regions, ensuring food security while mitigating environmental risks, with MB offering a scalable solution that outperforms other studies in biomass enhancement [9,44,46].

Comparative analysis of the four treatment groups untreated contaminated control, PB, bacterial inoculation, and MB revealed significant differences in their efficacy for reducing TF and BAF for Cd, Pb, Cu, and Cr in maize during a greenhouse experiment. MB exhibited the highest efficacy, reducing TF and BAF by 65–75% across all metals compared to the untreated contaminated control (p < 0.001). Bacterial inoculation achieved moderate reductions (40–50%, p < 0.01), outperforming PB (20–35%, p < 0.05), while the untreated contaminated control showed minimal changes (0–5%, p > 0.05). Compared to uncontaminated soil, MB’s reductions brought metal uptake to near background levels (p < 0.001), highlighting its superior potential for minimizing contaminant transfer and enhancing food safety in contaminated agricultural systems (Table 6, Fig 6).

The enhanced plant performance observed in the MB treatment, characterized by increased chlorophyll content, reduced metal accumulation, and higher biomass, demonstrates the efficacy of integrating microbially modified biochar with native bacterial consortia in mitigating contaminant induced stress in maize. The significantly higher chlorophyll content in the MB treatment (38.0 ± 2.5 SPAD vs. 25.0 ± 1.5 SPAD in control, p < 0.001) suggests improved photosynthetic capacity, likely due to reduced heavy metal toxicity and petroleum hydrocarbon stress, which are known to impair chlorophyll synthesis and trigger oxidative damage [47,48]. This aligns with findings by Afzal et al. [49], who reported a 20% increase in chlorophyll content in maize grown in biochar amended contaminated soils, though our 52% increase indicates a stronger effect, possibly due to the synergistic action of bacterial degradation and biochar adsorption. The low TF and BAF values in MB (e.g., TF for Cd: 0.27 vs. 0.95 in control; BAF for Pb: 0.35 vs. 1.20 in control, p < 0.001) demonstrate effective restriction of metal translocation from soil to shoots, reducing phytotoxicity and enhancing food safety, consistent with Anbuganesan et al. [50], who noted a 40% reduction in Cd uptake with biochar bacteria combinations. The substantial biomass increase in MB (100% higher than control, 30 g vs. 15 g shoot dry weight) exceeds the 30% increase reported by Wu et al. [17] likely due to the tailored bacterial consortium enhancing nutrient cycling and contaminant degradation. Although antioxidant enzyme activities were not measured, the improved chlorophyll and biomass suggest reduced oxidative stress, as heavy metals and hydrocarbons typically induce reactive oxygen species (ROS) production, which inhibits growth [51]. This limitation highlights the need for future studies to quantify enzymes such as superoxide dismutase or catalase to directly confirm ROS mitigation.

The exceptional performance of MB can be attributed to the synergistic interaction between the biochar’s enhanced physicochemical properties and the bioremediation capabilities of the bacterial consortium. The increased surface area (320 m²/g vs. 285 m²/g in PB) and pore volume (0.17 cm³/g vs. 0.15 cm³/g), coupled with bacterial colonization as confirmed by nitrogen presence in FESEM-EDS analysis, facilitated greater adsorption and immobilization of metals, restricting their translocation from soil to maize tissues. This aligns with findings by Xiang et al. [43] which reported that biochar bacteria interactions enhance pollutant removal through combined adsorption and microbial degradation. In contrast, the Bacteria treatment relied solely on microbial biosorption, achieving moderate reductions in metal uptake, while the PB treatment depended on adsorption alone, which was less effective without microbial enhancement. The control group’s poor performance highlights the necessity of amendments in contaminated soils. These findings suggest that MB offers a sustainable solution for mitigating metal toxicity in crops, particularly in arid regions like Kerman, where soil contamination threatens agricultural safety. However, long term field studies are needed to assess the stability of immobilized metals and ensure no secondary pollution occurs [52].

The superior remediation efficiency of MB can be directly linked to its enhanced physicochemical features. Compared to PB, MB showed a higher surface area (320 m²/g) and greater pore volume (0.17 cm³/g), providing more active adsorption sites. FT-IR spectra confirmed the presence of abundant carboxyl, hydroxyl, and carbonyl functional groups, while FESEM-EDS identified nitrogen incorporation from bacterial colonization, which enhanced metal complexation and hydrocarbon degradation potential. The higher fixed carbon content (64%) and stable H/C ratio further indicated long term structural stability of MB. These properties collectively explain MB’s superior remediation efficiency, with its increased surface area and nitrogen containing functional groups promoting 45–55% heavy metal immobilization (e.g., Cd reduced from 80 ± 3–50 ± 2 mg kg^-1^) and 70% petroleum hydrocarbon degradation (e.g., from 1000 ± 20–600 ± 12 mg kg^-1^) compared to PB (18–25% and 30%, respectively). The data underscore the synergistic role of microbial modification in enhancing biochar’s pollutant binding capacity. These improvements are consistent with previous studies, where biochars with enriched surface functionality and microbial colonization significantly enhanced contaminant immobilization and microbial activity [53–56]. Collectively, these data explain the observed higher reductions in heavy metal bioavailability (45–55%) and hydrocarbon degradation (70%) achieved by MB in contaminated soils.

The individual effect of PB is primarily driven by physical adsorption via its porous structure (surface area 285 m²/g) and functional groups (FT-IR peaks at 3400 cm ⁻ ¹ O-H, 1100 cm ⁻ ¹ C-O;), immobilizing metals through ion exchange and complexation. Bacterial inoculants contribute via enzymatic degradation and biosorption, with the consortium producing siderophore and dioxygenases, reducing contaminants through metabolic pathways. The synergy in MB arises from biochar providing a protective habitat for bacterial colonization, leading to a 20–30% greater efficiency than the sum of individual treatments, as bacteria enhance biochar’s functional groups (additional N-H peaks at 1550 cm ⁻ ¹) for improved metal binding and hydrocarbon breakdown. This interaction also boosted soil organic carbon (2.0 ± 0.3% in MB vs. 1.2 ± 0.2% in control) and maize performance (shoot biomass 30 g vs. 15 g in control, p < 0.001; Table 5), confirming the added value of the combination beyond isolated applications.

The observed remediation efficiency in this study, particularly the 45–55% reduction in heavy metal bioavailability and 70% degradation of petroleum hydrocarbons with MB, can be attributed to a synergistic combination of adsorption, microbial degradation, and complexation mechanisms. Adsorption primarily occurs through the biochar’s porous structure and functional groups, as evidenced by FT-IR analysis, where peaks at 3400 cm ⁻ ¹ (O-H) and 1100 cm ⁻ ¹ (C-O) in MB indicate enhanced metal binding sites compared to PB, facilitating the immobilization of metals like Cd and Pb via surface complexation and ion exchange. FESEM-EDS data further supports this, showing bacterial aggregates on MB’s surface with increased oxygen and nitrogen content (3 ± 0.5% N), which promote the formation of stable metal complexes, reducing bioavailability as seen in the lower TF and BAF values (TF for Cd: 0.27 in MB vs. 0.95 in control). Microbial degradation is driven by the enzymatic activities of the bacterial consortium, identified via 16S rRNA sequencing, which produce dioxygenases and siderophore to break down hydrocarbons (e.g., phenanthrene and pyrene) and biosorb metals, as demonstrated by the 60–85% degradation rates in isolated strains. Complexation is enhanced by EDS, visible in FESEM images (Fig 5B), which form chelates with metals, further reducing mobility and uptake in maize. This multi mechanistic approach explains MB’s superiority over PB (18–25% metal reduction) and bacterial inoculants alone (28–35%), where adsorption and degradation act independently, highlighting the study’s contribution to understanding integrated remediation in arid soils.

This results, are highly consistent with recent literature on biochar–microorganism synergies (2023–2025). Similar multi mechanistic action involving adsorption (O-H and C-O functional groups), microbial enzymatic degradation (dioxygenases, siderophores), and complexation via extracellular polymeric substances (EPS) has been reported by Xu et al. [53] (67% PAH degradation, 50% metal immobilization), Zhang et al. [54] (60–75% TPH/metal reduction), and Najim et al. [55] (55–78% dual remediation), all confirming enhanced performance when bacteria are immobilized on biochar surfaces (increased N content and biofilm formation observed by FESEM-EDS). The slightly higher TPH degradation recorded here (70%) falls within the upper range of these studies and can be attributed to the use of a locally adapted multi strain consortium and optimized biochar functionalization, while the 45–55% metal bioavailability reduction aligns closely with the 40–55% values commonly reported. Thus, our findings strongly corroborate the current consensus that integrated biochar–microbe systems outperform individual biochar or bacterial treatments through synergistic adsorption, biodegradation, and complexation pathways in co contaminated soils.

The observed FTIR shifts and persistent amide II peak provide unequivocal evidence of long term survival and metabolic activity of the bacterial consortium (*P. fluorescens, R. qingshengii, B. metallica, B. cereus, S. pactum*) on the biochar surface throughout the 90 day remediation period. The progressive red shift of the O–H band (–28 cm ⁻ ¹) and the sustained intensity of the amide II/N–H peak (1548–1555 cm ⁻ ¹) confirm continuous regeneration of nitrogen rich binding sites through bacterial cell wall proteins and amino sugars. EDS elemental analysis further corroborates stable microbial colonization, with surface nitrogen increasing from 0.98% in pristine biochar to 3.67% after remediation, accompanied by clear accumulation of Cd (1.58%) and Pb (2.34%) exclusively on microbially modified biochar. Elemental mapping revealed strong spatial co localization of Cd and Pb with nitrogen rich microbial zones (Cd/N = 0.43, Pb/N = 0.64), directly demonstrating biosorption via metal–amine coordination on bacterial cell surfaces. Sequential extraction results reinforce this mechanism, showing a dramatic reduction in bioavailable fractions (exchangeable + carbonate bound) of Cd (from 76% to 14.8%) and Pb (from 68% to 8.7%), with >68% of both metals transferred to stable organic matter bound and residual fractions. This shift reflects irreversible immobilization through biosorption onto microbial biomass followed by occlusion within the biochar–microbe aggregates. Thus, microbial modification transforms inert biochar into a living, self-regenerating bio system that synergistically heightened heavy metal immobilization through direct biosorption and complexation while sustaining high petroleum hydrocarbon degradation activity. This integrated approach offers a superior, durable, and ecologically compatible remediation strategy for co contaminated calcareous soils [57].

In total, the importance of these findings lies in their potential to address the pressing issue of soil contamination in arid agricultural regions, where traditional remediation methods are often costly and less effective. MB success can be attributed to the synergistic interplay between biochar’s physical adsorption properties and the metabolic activities of the bacterial consortium, tailored to local contaminants. This approach not only mitigates pollution but also supports crop productivity, as evidenced by maize shoot and root dry weights reaching 30 g and 18 g with MB, compared to 15 g and 8 g in the control (p < 0.001). Compared with previous studies, the performance of the MB treatment demonstrates several important distinctions. While Liu et al. [42] reported 40–50% reductions in heavy metal availability, the 45–55% decrease observed here appears to be enhanced by the use of native bacteria adapted to Kerman’s calcareous and multi contaminated soils. Hydrocarbon removal also exceeded values commonly reported in the literature; for example, the 70% reduction in our study surpasses the 60% degradation recorded by Hossain et al. [58], likely reflecting the targeted selection of PAH degrading isolates. Patterns in metal uptake further support the synergistic role of microbial augmentation: Afzal et al. [49] documented a Cd translocation factor of 0.35 with biochar alone higher than the 0.27 achieved with MB and far below the PB value of 0.65—highlighting the added functional contribution of the bacteria. Similar trends are seen in hydrocarbon studies using non native strains; the 50–60% degradation efficiency reported by Kumar et al. [38] demonstrates the advantage of locally adapted microorganisms under harsh soil conditions. Plant growth responses followed the same pattern, with maize biomass increases reported by Khan et al. [59] (≈30%) remaining well below the 100% improvement observed under MB, reflecting differences in soil type, biochar modification, and microbial compatibility. Nonetheless, concerns raised by Xu et al. [33] about the long term stability of immobilized metals emphasize the need for future field scale monitoring to evaluate potential secondary pollution risks.

While the MB treatment demonstrated significant reductions in heavy metal and petroleum hydrocarbon, alongside enhanced maize growth in a 60 day greenhouse experiment, several limitations restrict the generalizability of these findings. The short term, controlled conditions do not fully replicate field variables such as climate fluctuations, soil heterogeneity, or long term environmental dynamics, potentially overestimating MB’s performance. Biochar aging, driven by oxidation or microbial activity, may reduce adsorption capacity over time, as oxygen containing functional groups) could weaken metal binding efficiency [60]. Bacterial survival, particularly of species like *Pseudomonas fluorescens* and *Rhodococcus qingshengii*, may be limited in arid field conditions due to competition, nutrient depletion, or desiccation, potentially diminishing the observed synergistic degradation (70% vs. 50% in bacteria only treatment). Heavy metal remobilization risks, driven by changes in soil pH (7.8 ± 0.2) or redox conditions, could increase solubility of metals like Cd and Pb, leading to secondary pollution [61]. Scalability challenges, including biochar production costs ($500–1000 per ton for rice husk biochar) and logistical constraints of bacterial inoculation, further limit immediate field scale applicability in large contaminated areas like Kerman. Future research should prioritize multiyear field trials to validate MB’s long term stability, assess bacterial viability via 16S rRNA sequencing, and monitor metal immobilization durability using sequential extraction methods. Additionally, exploring a broader range of native microbial strains, evaluating nutrient imbalances or microbial community shifts, and investigating cost effective production methods will be crucial to optimize remediation efficiency and ensure environmental safety for sustainable agricultural applications in arid, contaminated regions. Although significant improvements in maize biomass, height, root development, and chlorophyll content clearly demonstrate enhanced plant performance under combined heavy metal and petroleum hydrocarbon stress, the present study did not include biochemical assays of oxidative stress markers (e.g., activities of catalase, peroxidase, and superoxide dismutase; malondialdehyde content) or histochemical localization of heavy metals in plant tissues. Future studies should incorporate these analyses to elucidate the precise physiological and molecular mechanisms underlying the observed stress mitigation and to further validate the protective role of microbially modified biochar at the cellular level.

## Conclusion

The results of this 90 day greenhouse pot experiment demonstrate that microbially modified biochar, inoculated with native hydrocarbon and metal tolerant bacterial isolates, significantly outperforms pristine biochar and bacterial inoculation alone in reducing bioavailability of Cr, Pb, Cd, Cu, and TPH while concurrently improving soil physicochemical properties (organic carbon, CEC, nutrient availability) and maize growth parameters. These findings, obtained under controlled short term conditions, highlight the synergistic potential of this combined approach for remediation of co contaminated soils. However, extrapolation to field scale or long term scenarios requires additional validation studies conducted under real world agricultural conditions. Nonetheless, the observed improvements in soil quality and plant performance provide a strong foundation for future translational research aimed at sustainable management of heavy metal and hydrocarbon contaminated agricultural lands.

## Supporting information

S1 Table Representative example of contaminant reduction and index calculation for Cd under the MB treatment. (DOCX)

S2 Table Representative calculation of soil heavy metal reduction efficiency after 90 days. (DOCX)

S3 Table Representative calculation of TPH degradation efficiency. (DOCX)

S4 Table Representative calculation of maize growth enhancement and physiological indices after 90 days. (DOCX)

S5 Table Statistical Summary: Two-way ANOVA and Principal Component Analysis (PCA) results. (DOCX)
