# Supplementary material for: Revitalizing contaminated soils: The combined power of modified biochar and intrinsic bacteria for heavy metal and petroleum hydrocarbon removal and plants performance
Source: PLoS One. 2026 Jun 24;21(6):e0349599. doi: 10.1371/journal.pone.0349599 (PMC13293394; doi:10.1371/journal.pone.0349599)
Supplement: S1 Table — (DOCX) [file pone.0349599.s001.docx]

**Table S1. Representative example of contaminant reduction and index calculation for Cd under the MB treatment**

| **Parameter** | **Value** | **Unit** | **Calculation** |
| --- | --- | --- | --- |
| Initial soil Cd concentration | 80 | mg kg⁻¹ | Measured before treatment |
| Final soil Cd concentration (MB) | 50 | mg kg⁻¹ | Measured after 90 days |
| Cd concentration in roots | 18 | mg kg⁻¹ | AAS analysis |
| Cd concentration in shoots | 5 | mg kg⁻¹ | AAS analysis |
| Contaminant reduction (%) | 37.5 | % | [(80 − 50) / 80] × 100 |
| Transfer Factor (TF) | 0.28 | – | Cd_shoot / Cd_root |
| Bioaccumulation Factor (BAF) | 0.06 | – | Cd_shoot / Cd_soil |
