# Supplementary material for: Revitalizing contaminated soils: The combined power of modified biochar and intrinsic bacteria for heavy metal and petroleum hydrocarbon removal and plants performance
Source: PLoS One. 2026 Jun 24;21(6):e0349599. doi: 10.1371/journal.pone.0349599 (PMC13293394; doi:10.1371/journal.pone.0349599)
Supplement: S2 Table — (DOCX) [file pone.0349599.s002.docx]

**Table S2. Representative calculation of soil heavy metal reduction efficiency after 90 days**

| Treatment | Initial Pb concentration | Final Pb concentration | Reduction (%) | Calculation |
| --- | --- | --- | --- | --- |
| Contaminated control | 600 | 570 | 5.0 | [(600 − 570) / 600] × 100 |
| Pristine biochar (PB) | 600 | 500 | 16.7 | [(600 − 500) / 600] × 100 |
| Bacterial inoculation | 600 | 480 | 20.0 | [(600 − 480) / 600] × 100 |
| Modified biochar (MB) | 600 | 400 | 33.3 | [(600 − 400) / 600] × 100 |
