# Supplementary material for: Revitalizing contaminated soils: The combined power of modified biochar and intrinsic bacteria for heavy metal and petroleum hydrocarbon removal and plants performance
Source: PLoS One. 2026 Jun 24;21(6):e0349599. doi: 10.1371/journal.pone.0349599 (PMC13293394; doi:10.1371/journal.pone.0349599)
Supplement: S3 Table — (DOCX) [file pone.0349599.s003.docx]

**Table S3. Representative calculation of TPH degradation efficiency**

| **Treatment** | **Initial TPH concentration** | **Final TPH concentration** | **Degradation (%)** | **Calculation** |
| --- | --- | --- | --- | --- |
| Contaminated control | 1000 | 950 | 5.0 | [(1000 − 950) / 1000] × 100 |
| Pristine biochar (PB) | 1000 | 800 | 20.0 | [(1000 − 800) / 1000] × 100 |
| Bacterial inoculation | 1000 | 750 | 25.0 | [(1000 − 750) / 1000] × 100 |
| Modified biochar (MB) | 1000 | 600 | 40.0 | [(1000 − 600) / 1000] × 100 |
