# Supplementary material for: Revitalizing contaminated soils: The combined power of modified biochar and intrinsic bacteria for heavy metal and petroleum hydrocarbon removal and plants performance
Source: PLoS One. 2026 Jun 24;21(6):e0349599. doi: 10.1371/journal.pone.0349599 (PMC13293394; doi:10.1371/journal.pone.0349599)
Supplement: S4 Table — (DOCX) [file pone.0349599.s004.docx]

**Table S4. Representative calculation of maize growth enhancement and physiological indices after 90 days**

| **Parameter** | **Contaminated control** | **PB** | **Bacterial inoculation** | **MB** | **Improvement over control (%)** | **Calculation (MB)** |
| --- | --- | --- | --- | --- | --- | --- |
| Shoot dry weight (g plant⁻¹) | 15.0 | 22.0 | 25.0 | 30.0 | 100.0 | [(30 − 15) / 15] × 100 |
| Root dry weight (g plant⁻¹) | 8.0 | 12.0 | 14.0 | 18.0 | 125.0 | [(18 − 8) / 8] × 100 |
| Plant height (cm) | 15.0 | 18.0 | 19.0 | 22.0 | 46.7 | [(22 − 15) / 15] × 100 |
| Root length (cm) | 8.0 | 9.5 | 10.0 | 11.5 | 43.8 | [(11.5 − 8) / 8] × 100 |
| Chlorophyll content (SPAD) | 25.0 | 32.0 | 34.0 | 38.0 | 52.0 | [(38 − 25) / 25] × 100 |
