# Supplementary material for: Revitalizing contaminated soils: The combined power of modified biochar and intrinsic bacteria for heavy metal and petroleum hydrocarbon removal and plants performance
Source: PLoS One. 2026 Jun 24;21(6):e0349599. doi: 10.1371/journal.pone.0349599 (PMC13293394; doi:10.1371/journal.pone.0349599)
Supplement: S5 Table — (DOCX) [file pone.0349599.s005.docx]

**Table S5 Statistical Summary: Two-way ANOVA and Principal Component Analysis (PCA) results**

| **Parameter / Variable** | **ANOVA Source** | **df** | **F-value** | **p-value** | **PCA – PC1 Loading** | **PCA – PC2 Loading** |
| --- | --- | --- | --- | --- | --- | --- |
| Bioavailable Cr | Treatment | 3 | 89.14 | <0.001 | –0.94 | 0.12 |
| Bioavailable Pb | Treatment | 3 | 92.37 | <0.001 | –0.96 | 0.09 |
| Bioavailable Cd | Treatment | 3 | 84.61 | <0.001 | –0.93 | 0.15 |
| Bioavailable Cu | Treatment | 3 | 78.29 | <0.001 | –0.91 | 0.11 |
| Total Petroleum Hydrocarbons (TPH) | Treatment | 3 | 142.68 | <0.001 | –0.97 | –0.08 |
| Soil organic carbon (SOC) | Treatment | 3 | 68.19 | <0.001 | 0.95 | 0.20 |
| Cation exchange capacity (CEC) | Treatment | 3 | 71.45 | <0.001 | 0.92 | 0.25 |
| Soil pH | Treatment | 3 | 12.88 | <0.001 | 0.18 | 0.88 |
| Available phosphorus | Treatment | 3 | 59.33 | <0.001 | 0.89 | 0.31 |
| Maize shoot dry biomass | Treatment | 3 | 98.37 | <0.001 | 0.96 | 0.16 |
| Maize root dry biomass | Treatment | 3 | 76.54 | <0.001 | 0.94 | 0.19 |
| Plant height | Treatment | 3 | 82.19 | <0.001 | 0.93 | 0.22 |
| Chlorophyll content (SPAD) | Treatment | 3 | 65.72 | <0.001 | 0.90 | 0.28 |
| **Residual (Error)** | – | 56 | – | – | – | – |
| **Total** | – | 59 | – | – | – | – |

**PCA Summary**

- Eigenvalue PC1: 9.42
- Eigenvalue PC2: 2.42
- Variance explained by PC1: 67.3%
- Variance explained by PC2: 17.3%
- Cumulative variance (PC1 + PC2): 84.6%

**Post-hoc test**: Tukey’s HSD (α = 0.05) – all treatment means significantly different from control for parameters with p < 0.001.
