# Supplementary material for: Revitalizing contaminated soils: The combined power of modified biochar and intrinsic bacteria for heavy metal and petroleum hydrocarbon removal and plants performance
Source: PLoS One. 2026 Jun 24;21(6):e0349599. doi: 10.1371/journal.pone.0349599 (PMC13293394; doi:10.1371/journal.pone.0349599)
Supplement: Attachment — Submitted filename: PLoS_Biochar_contaminant_rev.pdf [file pone.0349599.s006.pdf]

Overall
This manuscript highlights the effectiveness of modified biochar and native microbes on improving soil properties, nutrient availability, improved plant growth, and reductions in contaminant bioavailability. Overall, the methods are appropriate, and the results are clearly described and well contextualized. The manuscript, in my opinion, generally meets the criteria necessary for publication in PLOS One. My suggestions for improvement are generally related to clarity. I believe the manuscript could benefit from alterations particularly to the results and discussion sections.

General
As is, the data availability statement is insufficient as it is difficult to find the data with just the information given.

Because copy-editing is not provided by PLOS One, I have noted small mistakes and inconsistencies where I found them.

- Define acronyms/abbreviations upon first use:
    - Line 72: GC-MS, Line 83: AAS, Line 110: FTIR, Line 111: FESEM-EDS,
- Be consistent when talking about modified biochar. Throughout the manuscript it is “Biochar-M”, “MB”, and “M-Biochar”. I suggest choosing one abbreviation only for consistency.
- The references in this manuscript are in number format (i.e. [#]). However, the in-text citations are often written as year format (e.g., “...as shown by Maharlouei et al (2021)...”). I believe these in-text citations should be changed to reflect the number of that reference (e.g., “...as shown by Maharlouei et al [7]...”). I would double check with the reference guidelines.
    - Lines: 46, 193, 303, 305, 328, 339, 340, 350, 352, 355, 377, 394, 395, 397, 399, 400, and 402
- Unit consistency: I would suggest altering unit formatting from “mg/kg” to “mg kg^{-1}" especially for units such as in Line 138
- Figure references in text: Throughout the manuscript figures are often references like “Figure4” instead of “Figure 4” please fix throughout.
- Because many analyses were performed (which is good), there are many subsections in the Methods and Results, which can sometimes be confusing. Throughout the manuscript, alter the headings to better show the sections. I looked at the PLOS One guidelines and it appears that they prefer to have different heading levels. See below:

Materials and Methods (Level 1 – bold, 18pt font)

Soil Characterization (Level 2 – bold, 16pt font)

Physical and Chemical Analysis of Soil (Level 3 – bold, 14pt font)

Bacterial Isolation and Identification (Level 2 – bold, 16pt font)

Isolation and conventional of biodegradable strains (Level 3 – bold, 14pt font)

Molecular isolation of biodegradable strains (Level 3 – bold, 14pt font)

Bioremediation analysis... (Level 2 – bold, 16pt font)

Section-specific suggestions

Line numbering started at the Methods, so I tried my best to describe where I had suggestions in the Abstract and Introduction.

Abstract

General:

- The abstract would be strengthened by the inclusion of a “big picture” statement to contextualize why this work matters. Something short that details why contaminated soils are bad for either people or plant production. You already included ‘essential to protect agricultural productivity’ in the first sentence, but this could be expanded to make the importance of the work clearer.

Line-by-line:

- Sentence 2: add space between biochar and (MB)
- Sentence 3: try to avoid starting a sentence with a number – personally I would just start off as “A total of 30 samples...” to avoid this
- Sentence 6: “isolated and from” - I think deleting the “and” would make sense here
- Sentence 6: Is “Biochar-M” the same as MB? If so, I would choose one abbreviation and be consistent throughout the manuscript. If not, I suggest clearly defining the two abbreviations.

Introduction

General:

I found the introduction to be well-written, informative, and a great start to the manuscript. The only general suggestion I have for its improvement would be in the last paragraph. The sentences in this paragraph are long, which makes it difficult to separate your objectives from your analysis. Below I have included a draft example of how I would alter the paragraph. Feel free to use it as-is, adapt it, or figure out another way to improve clarity.

- “Given these challenges, and the limited success of standalone biochar or bacterial remediation, there is a need for integrated and sustainable strategies tailored to Iran’s calcareous soils. Therefore, this study pursued three objectives: (**i**) characterize contaminated soils in Kerman Province, (**ii**) isolate indigenous bacterial species with high heavy metal and petroleum hydrocarbon degradation potential, and (**iii**) evaluate the synergistic effects of NaOH-modified rice husk biochar and these bacteria on pollutant bioavailability, soil microbial activity, and maize growth under greenhouse conditions. By linking soil characterization with remediation trials, this work aims to provide a framework for developing effective, locally adapted approaches to soil remediation.”

Line-by-line:

- Paragraph 5 Sentence 3: I would like to see a citation or example of safe limits of these concentrations.

Materials and Methods

Line-by-line:

- Figure 1 caption: Change to "...The figure was designed using Adobe..."
- Line 47: should be "follows"
- Line 50: Is the 1:1 solution by volume (i.e. v/v) or by weight (w/w)?
    - Also Line 69, 80

Results

General:

- In my opinion, the headings of the results sections could be altered to summarize the findings of that section. Here are my suggested changes. The first bullet point is a more "finding-focused" whereas the second bullet point is more "method-focused". I personally prefer results section headers that are finding-focused:
    - Soil characterization
        - Method-focused: Physicochemical and contaminant profile of soils
    - Bacterial identification
        - Finding focused: Indigenous strains display strong remediation potential
        - Method-focused: Selection of high-potential bioremediating strains
    - Physiochemical analysis of biochar
        - Microbial modification enhances biochar properties
        - Characterization of pristine and microbially modified biochar
    - Greenhouse experiment
        - Biochar–bacteria treatments boost maize growth
        - Greenhouse evaluation of maize growth under different treatments
    - Heavy metal immobilization
        - Synergistic biochar-bacteria treatments reduce heavy metal bioavailability
        - Assessment of treatment effects on heavy metal immobilization
    - Petroleum hydrocarbon degradation
        - Modified biochar accelerates hydrocarbon breakdown
        - Evaluation of petroleum hydrocarbon degradation by treatments
    - TF and BAF in Maize for heavy metal
        - Biochar-bacteria treatments lower heavy metal uptake in maize
        - Translocation and bioaccumulation of heavy metals in maize

- In the Results section, many numerical values are already presented in tables and figures. I suggest focusing the text on whether the changes were statistically significant and on the size of the change, instead of repeating all the numbers. I have made example suggestions of this in the line-by-line section. However, I would suggest the authors to consider this throughout. You did this well in lines 260-267.

Line-by-line:

- Lines 166-170: I would suggest comparing to the uncontaminated soil
- Figure 4 caption: Change to "FT-IR spectrum of pristine biochar (PB) and M-biochar (MB)."
  - In the manuscript I've seen FT-IR and FTIR, try and be consistent
- Figure 5 caption: Change to "FESEM image of (a) pristine biochar and (b) M-biochar."
- 228-239: Make this section clearer. As is, it is difficult to tell when certain treatments did better than the control or just better than the untreated, contaminated control.
  - Please clarify whether the treatments improved growth only compared to the contaminated soil, or also compared to the uncontaminated control
  - Maybe add a sentence summary at the end like this: "While all treatments improved growth compared to the contaminated control, the performance of MB even surpassed that of the uncontaminated soil. The superior performance of MB suggests synergistic effects between biochar's adsorption capacity and bacterial degradation, fostering a favorable environment for plant growth ($p < 0.01$; Table 4)."
- 230: isn't this missing a treatment? You talk about "Bacterial inoculants" later in the paragraph, but that treatment is not listed in this sentence.
- 246: no detectable metals for the uncontaminated soil. However, Figure 6 and the tables show value for the uncontaminated soils, please check/clarify. Are they below detection limit?
- 245-254: Could be improved to enhance clarity – I suggest altering the results to showcase the percent change rather than listing the actual numbers (you can just cite the Table). This way it is easier for the reader to determine the amount of change:
  - "Compared to the contaminated soil, all treatments reduced metal concentrations (Table 4). MB generally achieved the greatest reduction ($p < 0.001$), lowering Cr, Pb, Cd, and Cu by 40.0 ± Y, 33.3 ± Y, 37.5 ± Y, and 40.0 ± Y%, respectively. Bacterial inoculants decreased these concentrations by 20-27% whereas PB only achieved 16.7-20% reductions. The untreated control showed minimal, insignificant changes ($p > 0.05$) with concentrations of Cr, Pb, Cd, and Cu concentrations only reducing by 1.7-3% (Table 4)."
    - Please do not rely on my quick example calculations, as I did not include errors or standard deviations. If taking my suggestion, please redo all analyses and do not take my statistical assumptions (i.e. retest them).

        - Besides MB treatment, only Cr and Pb are compared. I did not see a reason mentioned.
- Figure 6: I would like to see some identification of statistical significance. For example an asterix could be added above the bars that are significantly different than the values of the contaminated soil. If this is done, add a description of this in the figure caption. Example: "Asterix (*) show values are significantly different ($p < 0.05$) than the contaminated soil."
- Line 265: "the control" can be confusing since you have multiple (i.e. uncontaminated soil, contaminated soil, and the without treatment). Throughout the results I would specify the control by using something like: "the without treatment control" or the "untreated control".
- Figure 7: The image quality of this figure is low. Please try to improve the resolution for final submission.
- Line 269-270: should be "Chromatograms of petroleum hydrocarbon for (a) standard reference of TPH and the following experimental conditions: (b) without treatment, (c) PB, (d) bacterial inoculation, and (e) MB."

Discussion

General:

- The Discussion contains many numbers that are already reported in the Results and tables. I suggest summarizing the main findings and citing the appropriate table or figure, instead of restating the numerical values.
    - Example (Lines 294-298): instead of listing the percentage of hydrocarbon degradation and heavy metal removal from each you could add a range for all and cite the figure.
        - "with five isolates selected for their superior bioremediation potential (60-85% petroleum hydrocarbon degradation, 60-80% heavy metal removal): P. fluorescens, R. qingshengii, B. metallica, B. cereus, and S. pactum (Figure 2)."
- I suggest adding clear subsections in the Discussion to separate the parts about hydrocarbon degradation/removal and the parts about plant growth. These are major findings that deserve more emphasis:
    - Header suggestions:
        - Between Lines 291 and 292: Synergistic effects of native bacterial consortia and biochar on soil remediation
        - Between Lines 341 and 342: Enhanced maize growth and reduced metal uptake under combined treatments
        - Between Lines 386 and 387: Implications, limitations, and future directions for sustainable soil remediation

Line-by-line:

- Lines 312-330: very results heavy
