# Supplementary material for: Revitalizing contaminated soils: The combined power of modified biochar and intrinsic bacteria for heavy metal and petroleum hydrocarbon removal and plants performance
Source: PLoS One. 2026 Jun 24;21(6):e0349599. doi: 10.1371/journal.pone.0349599 (PMC13293394; doi:10.1371/journal.pone.0349599)
Supplement: Attachment — Submitted filename: Major Revisions.pdf [file pone.0349599.s008.pdf]

Major Revision (still required before acceptance)

1. The stated novelty remains vague. Please clearly differentiate your study from recent (2022–2025) biochar-microbe remediation works and explicitly justify how the chosen iron oxide modification and local bacterial strains represent a new conceptual or mechanistic advance.

2. The greenhouse setup requires precise details, number of replicates, environmental parameters (temperature, humidity, light), randomization method, and statistical design. Without this, reproducibility and statistical validity cannot be confirmed.

3. Biochar data remain insufficient. Include a comprehensive table with quantitative properties (BET surface area, porosity, pH, elemental composition, C/N ratio, ash content) to support claims of enhanced adsorption and modification effects.

4. While contaminated soil data are presented, baseline (uncontaminated) soil properties are missing. Please provide comparative values for uncontaminated reference soil and add key parameters such as CEC and texture.

5. Mechanistic discussion relies mainly on FTIR and EDS visuals without quantitative backing. Provide spectral shift values, atomic percentages, or adsorption-desorption data to substantiate the proposed adsorption and complexation mechanisms.

6. The study identifies strains but lacks evidence of microbial dynamics after treatment. Include at least qualitative or quantitative data (e.g., CFU counts or community shifts) demonstrating bacterial survival or interaction within the biochar matrix.

7. The "plant performance" claim is based only on biomass and SPAD readings. Incorporate basic biochemical indicators (antioxidant enzymes, stress markers, or metal localization) to strengthen the biological relevance of plant improvement.

8. Although ANOVA and PCA were added, key statistical details are missing. Please report degrees of freedom, F-values, p-values, and percentage variance explained by multivariate analyses to ensure analytical transparency.

9. The conclusion still slightly overreaches. Limit claims to short-term greenhouse conditions and avoid terms like "revitalization" unless supported by field-scale or longterm validation data.

10. Revise figures for consistent labeling and higher resolution, ensure captions include full interpretation, and update the reference list with the most recent biochar-microbe studies (2023–2025) for better contextualization.

While the manuscript shows substantial improvement, key methodological and mechanistic gaps persist, particularly quantitative biochar characterization, replication details, and physiological validation of "revitalization." Addressing these before publication is essential to meet PLOS ONE's scientific standards.
