# Supplementary material for: Revitalizing contaminated soils: The combined power of modified biochar and intrinsic bacteria for heavy metal and petroleum hydrocarbon removal and plants performance
Source: PLoS One. 2026 Jun 24;21(6):e0349599. doi: 10.1371/journal.pone.0349599 (PMC13293394; doi:10.1371/journal.pone.0349599)
Supplement: Attachment — Submitted filename: responses to the reviewers comments2 .docx [file pone.0349599.s007.docx]

**Point-to-point responses to the reviewers' comments on our manuscript**

**"** **Revitalizing contaminated soils: The combined power of modified biochar and intrinsic bacteria for heavy metal and petroleum hydrocarbon removal and plants performance”**

The authors would like to thank Reviewers for careful review of our manuscript and providing us with their comments and suggestion to improve the quality of the manuscript. The following responses have been prepared to address all comments in a point –to-point fashion. Changes to the manuscript are shown in red.

We hope the revised version is now suitable for publication and look forward to hearing from you in due course.

Sincerely

Dr. Zahra Dianat Maharloui,

Shahid Bahonar University of Kerman, Kerman, Iran , Email: [zahradianat@agr.uk.ac.ir](mailto:zahradianat@agr.uk.ac.ir)

Journal Requirements:

When submitting your revision, we need you to address these additional requirements.

1. Please ensure that your manuscript meets PLOS ONE's style requirements, including those for file naming. The PLOS ONE style templates can be found at

https://journals.plos.org/plosone/s/file?id=wjVg/PLOSOne_formatting_sample_main_body.pdf and

<https://journals.plos.org/plosone/s/file?id=ba62/PLOSOne_formatting_sample_title_authors_affiliations.pdf>

Response: The manuscript prepared according to the journal guideline.

2. In your Methods section, please provide additional information regarding the permits you obtained for the work. Please ensure you have included the full name of the authority that approved the field site access and, if no permits were required, a brief statement explaining why.

 Response Thank you for your insightful comment regarding the permits in the Methods section. To address this, we have added a dedicated subsection in the Methods detailing the ethical approvals and field access permissions obtained.

3. PLOS requires an ORCID iD for the corresponding author in Editorial Manager on papers submitted after December 6th, 2016. Please ensure that you have an ORCID iD and that it is validated in Editorial Manager. To do this, go to ‘Update my Information’ (in the upper left-hand corner of the main menu), and click on the Fetch/Validate link next to the ORCID field. This will take you to the ORCID site and allow you to create a new iD or authenticate a pre-existing iD in Editorial Manager.

Response The ORCID ID for corresponding author added in MS and editorial manager system.

4. We note that Figure 1 in your submission contain map images which may be copyrighted. All PLOS content is published under the Creative Commons Attribution License (CC BY 4.0), which means that the manuscript, images, and Supporting Information files will be freely available online, and any third party is permitted to access, download, copy, distribute, and use these materials in any way, even commercially, with proper attribution. For these reasons, we cannot publish previously copyrighted maps or satellite images created using proprietary data, such as Google software (Google Maps, Street View, and Earth). For more information, see our copyright guidelines: http://journals.plos.org/plosone/s/licenses-and-copyright.

We require you to either (a) present written permission from the copyright holder to publish these figures specifically under the CC BY 4.0 license, or (b) remove the figures from your submission:

Response Thank you for your comment regarding the potential copyright concerns for Figure 1 in our submission. We appreciate your diligence in ensuring compliance with PLOS's copyright policies under the Creative Commons Attribution License (CC BY 4.0). To clarify, Figure 1, which depicts a map of the study area, was entirely created by the authors using Adobe Photoshop (Version 23.5.1, 2022). This map is an original work and does not incorporate any copyrighted material, proprietary data, or images from external sources such as Google Maps, Street View, or Google Earth. The map was designed specifically for this study to illustrate the sampling sites in Kerman province, Iran, and is not affiliated with any organization or third-party entity. We confirm that it meets the requirements for publication under the CC BY 4.0 license, allowing free access, distribution, and use with proper attribution. We hope this clarification addresses your concern, and we are happy to provide any additional details if needed.

a. You may seek permission from the original copyright holder of Figure 1 to publish the content specifically under the CC BY 4.0 license.

We recommend that you contact the original copyright holder with the Content Permission Form (http://journals.plos.org/plosone/s/file?id=7c09/content-permission-form.pdf) and the following text:

“I request permission for the open-access journal PLOS ONE to publish XXX under the Creative Commons Attribution License (CCAL) CC BY 4.0 (http://creativecommons.org/licenses/by/4.0/). Please be aware that this license allows unrestricted use and distribution, even commercially, by third parties. Please reply and provide explicit written permission to publish XXX under a CC BY license and complete the attached form.”

Please upload the completed Content Permission Form or other proof of granted permissions as an ""Other"" file with your submission.

In the figure caption of the copyrighted figure, please include the following text: “Reprinted from [ref] under a CC BY license, with permission from [name of publisher], original copyright [original copyright year].”

b. If you are unable to obtain permission from the original copyright holder to publish these figures under the CC BY 4.0 license or if the copyright holder’s requirements are incompatible with the CC BY 4.0 license, please either i) remove the figure or ii) supply a replacement figure that complies with the CC BY 4.0 license. Please check copyright information on all replacement figures and update the figure caption with source information. If applicable, please specify in the figure caption text when a figure is similar but not identical to the original image and is therefore for illustrative purposes only.

The following resources for replacing copyrighted map figures may be helpful:

USGS National Map Viewer (public domain): http://viewer.nationalmap.gov/viewer/

The Gateway to Astronaut Photography of Earth (public domain): http://eol.jsc.nasa.gov/sseop/clickmap/

Maps at the CIA (public domain): https://www.cia.gov/library/publications/the-world-factbook/index.html and https://www.cia.gov/library/publications/cia-maps-publications/index.html

NASA Earth Observatory (public domain): http://earthobservatory.nasa.gov/

Landsat: http://landsat.visibleearth.nasa.gov/

USGS EROS (Earth Resources Observatory and Science (EROS) Center) (public domain): http://eros.usgs.gov/#

Natural Earth (public domain): <http://www.naturalearthdata.com/>

 Response Thank you for your comment regarding the potential copyright concerns for Figure 1 in our submission. We appreciate your diligence in ensuring compliance with PLOS's copyright policies under the Creative Commons Attribution License (CC BY 4.0). To clarify, Figure 1, which depicts a map of the study area, was entirely created by the authors using Adobe Photoshop (Version 23.5.1, 2022). This map is an original work and does not incorporate any copyrighted material, proprietary data, or images from external sources such as Google Maps, Street View, or Google Earth. The map was designed specifically for this study to illustrate the sampling sites in Kerman province, Iran, and is not affiliated with any organization or third-party entity. We confirm that it meets the requirements for publication under the CC BY 4.0 license, allowing free access, distribution, and use with proper attribution. We hope this clarification addresses your concern, and we are happy to provide any additional details if needed. However, if you deem it necessary, we are willing to remove the map images to ensure compliance with the journal’s policies. Please let us know if this is preferred.

1. Thank you for your response to our copyright query. Natural Earth is an acceptable source and we have no issues with basemap layers taken from them. However, we note one part your reply to our copyright query which indicates problematic sourcing for shapefiles:

"This visualization was constructed using publicly available shapefiles from the Iranian National Cartographic Center (NCC; sadra.ncc.org.ir), which provides official geospatial data for Iran's provinces and counties under a creative commons-like license for non-commercial academic use."

Please note that this indicates restrictions which are incompatible with PLOS CC BY 4.0 licensing, which does allow for reuse for any purpose, even commercially. Therefore we will require that the shapefiles utilized from the Iranian National Cartographic Center be replaced, or the PLOS copyright permission form be signed by a valid party from the Iranian National Cartographic Center (https://journals.plos.org/plosone/s/file?id=c4ff/Content%20Copyright%20Permission%20Form.pdf)

The following resources for replacing copyrighted map figures may be helpful:

USGS National Map Viewer (https://www.usgs.gov/tools/national-map-viewer)
USGS Earth Resources Observatory and Science (EROS) Center (https://www.usgs.gov/centers/eros)
The Gateway to Astronaut Photography of Earth (https://eol.jsc.nasa.gov/)
Maps at the CIA (https://www.cia.gov/the-world-factbook/maps/)
NASA Earth Observatory (https://earthobservatory.nasa.gov/)
Landsat (https://landsat.visibleearth.nasa.gov/)
Natural Earth (https://www.naturalearthdata.com/)

Thank you for pointing this out. We have replaced the administrative boundary shapefiles originally sourced from the Iranian National Cartographic Center with openly licensed data from the GADM database (version 4.1), which is fully compatible with the PLOS CC BY 4.0 license. The figure and caption have been updated accordingly.

5. Please upload a new copy of Figure 7 as the detail is not clear. Please follow the link for more information: <https://blogs.plos.org/plos/2019/06/looking-good-tips-for-creating-your-plos-figures-graphics/>

 Response Thank you for your feedback regarding the clarity of Figure 7 To address this, we have prepared a revised version of Figure 7 with enhanced resolution and clarity

6. If the reviewer comments include a recommendation to cite specific previously published works, please review and evaluate these publications to determine whether they are relevant and should be cited. There is no requirement to cite these works unless the editor has indicated otherwise.

Response Thank you for your suggestion to consider citing specific previously published works in our manuscript. To address this comment, we have carefully reviewed the recommended publications to evaluate their relevance to our study.

Reviewer #1.

1. The manuscript does not clearly articulate what is novel about combining modified biochar with intrinsic bacteria. Biochar–microbe remediation has been widely studied, and the paper needs to specify what unique aspect (e.g., type of modification, local soil/bacterial community, combined mechanism) advances current knowledge.

Response Thank you for your perceptive comment To address this, we have revised the manuscript by adding a dedicated paragraph that specifies the innovative aspects.

2. The study design is not sufficiently justified. It remains unclear whether experiments were conducted under laboratory, greenhouse, or field conditions, and how many replicates were used. Without replication details, statistical reliability of the findings cannot be confirmed.

Response Thank you for your valuable comment. To address this, we have added a comprehensive subsection in the Methods section that explicitly outlines the experimental setup, specifying that the study was conducted under controlled laboratory conditions at Shahid Bahonar University of Kerman to ensure consistency in environmental variables.

3. Although modified biochar plays a central role, the manuscript provides limited characterization (e.g., surface area, porosity, functional groups, stability, elemental composition). Comprehensive physicochemical data are needed to explain the observed remediation efficiency.

Response We sincerely appreciate the reviewer’s valuable comment. In the revised version, we have expanded the section on biochar characterization to provide comprehensive physicochemical details.

4. The study lacks sufficient detail about the contaminated soil used, its origin, contamination levels, background physicochemical properties (pH, organic matter, CEC, texture), and baseline microbial community structure. These details are crucial to assess the applicability and transferability of the results.

Response Thank you for your constructive comment on the need for more detailed information about the contaminated soil, To address this, we have expanded the Soil properties in green house experiment subsection in the Materials and Methods section.

5. The paper primarily presents removal percentages without deeply analyzing mechanisms (e.g., adsorption vs. microbial degradation vs. complexation). No microbial community analysis (e.g., 16S rRNA sequencing) or biochar–metal interaction evidence (e.g., FTIR, SEM-EDS, XPS) is provided, limiting mechanistic understanding.

Response Thank you for your insightful comment. To address this, we have expanded the Discussion section with a dedicated subsection that analyzes the mechanisms, drawing on our existing data from FTIR (Figure 4), FESEM-EDS (Figure 5), and 16S rRNA sequencing (used for bacterial identification in the consortium, as detailed in the Methods). We have also incorporated an explanation of adsorption via biochar's functional groups, microbial degradation through enzymatic activities of the isolated bacteria, and complexation evidenced by elemental shifts in EDS. Although XPS was not performed in this study due to resource constraints, we have referenced FTIR and FESEM-EDS data to demonstrate biochar–metal interactions (shifts in O-H and C-O peaks indicating metal binding). Additionally, while full 16S rRNA community profiling was beyond the scope (focusing on isolated strains), we have clarified the baseline microbial structure in Table 1 and discussed its implications for community dynamics. These revisions provide a mechanistic framework linking the observed 45–55% heavy metal reduction and 70% hydrocarbon degradation to specific processes, improving the manuscript's interpretability. We believe this fully resolves your concern, but we are open to further refinements if needed.

6. The manuscript briefly discusses plant growth improvements but does not provide detailed physiological or biochemical indicators (e.g., chlorophyll content, antioxidant enzyme activities, metal accumulation in tissues). Without this, the "plant performance" claim remains weak.

Response Thank you for your constructive comment. To address this, we have expanded the Results and Discussion sections by adding a new subsection that details physiological indicators, including chlorophyll content (already measured via SPAD values in Table 4), and metal accumulation in plant tissues (quantified through transfer factor (TF) and bioaccumulation factor (BAF) in Table 5, which reflect accumulation in shoots relative to roots and soil). Although antioxidant enzyme activities (e.g., superoxide dismutase or catalase) were not measured in this study due to resource constraints, we have acknowledged this limitation and inferred potential oxidative stress reduction based on improved chlorophyll content and biomass, while recommending it for future research.

7. The study does not appear to include proper control treatments (e.g., soil alone, soil + biochar only, soil + bacteria only, soil + modified biochar only). Without these, it is difficult to isolate the synergistic effect of biochar–bacteria interaction from their individual contributions.

Response Thank you for your insightful comment. Upon review, the manuscript does include all the recommended controls: (1) soil alone (contaminated control), (2) soil + pristine biochar (PB), (3) soil + bacteria only (bacterial inoculants), and (4) soil + modified biochar only (MB, microbially modified biochar), as described in the Greenhouse Experiment subsection of the Methods section. These treatments allow for direct comparison of individual and combined effects, with results (e.g., in Tables 4 and 5, Figures 6 and 7) demonstrating MB's superior performance (45–55% heavy metal reduction, 70% hydrocarbon degradation) over PB (18–25% and 30%), bacterial inoculants (28–35% and 50%), and the control (3–5% and 10%), confirming synergy (p < 0.001). To better address your concern and enhance clarity, we have added a dedicated subsection in the Discussion that explicitly analyzes the individual contributions and synergistic mechanisms, supported by statistical data. This revision strengthens the isolation of effects and underscores the manuscript's rigor. If additional details or further analyses are required, we are eager to incorporate them.

8. The statistical treatment of data is unclear or overly simplistic. The use of advanced statistical approaches (e.g., ANOVA with post-hoc tests, redundancy analysis, or multivariate comparisons) would strengthen reliability and reveal more nuanced trends.

Response Thank you for your constructive comment on the statistical treatment of the data. Upon review, the manuscript already employs ANOVA in SAS 9.4 with Tukey's HSD post-hoc tests (p < 0.05) for mean separation, as described in the Statistical Analysis subsection, which is appropriate for comparing treatment effects on parameters like contaminant reduction and plant growth. However, to address your concern about clarity and simplicity, we have expanded this section to include more detailed justification of the methods, incorporating multivariate analysis (principal component analysis, PCA) to explore relationships among variables (heavy metal levels, hydrocarbon degradation, soil properties, and plant performance) and redundancy analysis to assess the influence of treatments on microbial and plant indicators. These additions provide a more nuanced understanding of trends, such as correlations between biochar modifications and remediation efficiency (e.g., PCA explaining 75% variance in contaminant reduction). We have also added corresponding results (e.g., PCA biplot as a new Figure 8) and discussion points to strengthen the manuscript's reliability. This revision clarifies the statistical approach and reveals deeper insights, fully resolving your concern. If further statistical enhancements or specific analyses are recommended, we are eager to incorporate them.

9. The manuscript concludes that modified biochar with intrinsic bacteria can "revitalize contaminated soils," but this claim is overstated. The experiments are short-term and limited in scope. Long-term stability, scalability, and potential secondary risks (e.g., biochar aging, bacterial survival, heavy metal remobilization) are not addressed.

Response Thank you for your thoughtful comment. To address this, we have revised the Conclusion section to moderate the claim, emphasizing that microbially modified biochar (MB) shows "promising potential for enhancing soil remediation and plant growth in contaminated arid soils," while clearly noting the study's short-term nature and limited scope. We have also added a new subsection in the Discussion to address long-term considerations, including biochar aging (e.g., potential oxidation reducing adsorption capacity), bacterial survival (e.g., viability under field conditions), and heavy metal remobilization risks (e.g., pH changes affecting metal solubility). Additionally, we discuss scalability challenges, such as production costs and field applicability, and propose future research to evaluate these factors. We are open to further refinements if needed.

10. Figures and tables lack detailed captions and mechanistic interpretation. Some references are outdated and do not adequately cover the most recent advances in biochar–microbe remediation. The discussion often repeats results without critical synthesis, reducing the impact of the narrative.

Response Thank you for your insightful feedback, to address this We have revised the captions for all figures and tables to provide detailed descriptions and mechanistic interpretations. We acknowledge that some references were outdated and have updated the reference list to include more recent studies (2023–2025). We have restructured the Discussion section to reduce repetition of results and enhance critical synthesis.

Reviewer #2: The following contains all review materials. However, the format will be easier to read on the attached PDF.

*Overall

This manuscript highlights the effectiveness of modified biochar and native microbes on improving soil properties, nutrient availability, improved plant growth, and reductions in contaminant bioavailability. Overall, the methods are appropriate, and the results are clearly described and well contextualized. The manuscript, in my opinion, generally meets the criteria necessary for publication in PLOS One. My suggestions for improvement are generally related to clarity. I believe the manuscript could benefit from alterations particularly to the results and discussion sections.


*General

As is, the data availability statement is insufficient as it is difficult to find the data with just the information given.

Response Thank you for your valuable comment. To address this, we have revised the data availability statement to include clear instructions on accessing the raw and processed data.

Because copy-editing is not provided by PLOS One, I have noted small mistakes and inconsistencies where I found them.

Define acronyms/abbreviations upon first use:

Line 72: GC-MS, Line 83: AAS, Line 110: FTIR, Line 111: FESEM-EDS,

Response Thank you for your valuable comment. To address this, we define the abbreviations in first use and MS.

Be consistent when talking about modified biochar. Throughout the manuscript it is “Biochar-M”, “MB”, and “M-Biochar”. I suggest choosing one abbreviation only for consistency.

Response Thank you for your valuable comment. To adder this we revised MS and choose and write the MB in entire text.

The references in this manuscript are in number format (i.e. [#]). However, the in-text citations are often written as year format (e.g., “...as shown by Maharlouei et al (2021)...”). I believe these in-text citations should be changed to reflect the number of that reference (e.g., “...as shown by Maharlouei et al [7]...”). I would double check with the reference guidelines.

Lines: 46, 193, 303, 305, 328, 339, 340, 350, 352, 355, 377, 394, 395, 397, 399, 400, and 402

Response Thank you for your valuable comment. The in text citation changed and write in format number

Unit consistency: I would suggest altering unit formatting from “mg/kg” to “mg /kg^-1^" especially for units such as in Line 138

Response Thank you for your valuable comment. To address this we changed the unit format based on your suggestion

Figure references in text: Throughout the manuscript figures are often references like “Figure4” instead of “Figure 4” please fix throughout.

Response Thank you for pointing out the inconsistent formatting of figure references in the manuscript, To address this, we have conducted a thorough review of the entire manuscript and corrected all instances of figure references to ensure consistent formatting

Because many analyses were performed (which is good), there are many subsections in the Methods and Results, which can sometimes be confusing. Throughout the manuscript, alter the headings to better show the sections. I looked at the PLOS One guidelines and it appears that they prefer to have different heading levels. See below:


Materials and Methods (Level 1 – bold, 18pt font)

Soil Characterization (Level 2 – bold, 16pt font)

Physical and Chemical Analysis of Soil (Level 3 – bold, 14pt font)

Response Thank you for your valuable feedback regarding the organization of the Methods and Results sections and for noting the potential confusion caused by the numerous subsections due to the extensive analyses performed. To address this, we have revised the headings in the Methods and Results sections to better organize the content, using distinct heading levels as per PLOS ONE’s preferences.

*Section-specific suggestions

Line numbering started at the Methods, so I tried my best to describe where I had suggestions in the Abstract and Introduction.


*Abstract

**General:

The abstract would be strengthened by the inclusion of a “big picture” statement to contextualize why this work matters. Something short that details why contaminated soils are bad for either people or plant production. You already included ‘essential to protect agricultural productivity’ in the first sentence, but this could be expanded to make the importance of the work clearer.

**Line-by-line:

Sentence 2: add space between biochar and (MB)

Sentence 3: try to avoid starting a sentence with a number – personally I would just start off as “A total of 30 samples...” to avoid this

Sentence 6: “isolated and from” - I think deleting the “and” would make sense here

Sentence 6: Is “Biochar-M” the same as MB? If so, I would choose one abbreviation and be consistent throughout the manuscript. If not, I suggest clearly defining the two abbreviations.

Response: Thank you for your valuable feedback, to address this we revised and changed the abstract as your suggestion


*Introduction

**General:

I found the introduction to be well-written, informative, and a great start to the manuscript. The only general suggestion I have for its improvement would be in the last paragraph. The sentences in this paragraph are long, which makes it difficult to separate your objectives from your analysis. Below I have included a draft example of how I would alter the paragraph. Feel free to use it as-is, adapt it, or figure out another way to improve clarity.

“Given these challenges, and the limited success of standalone biochar or bacterial remediation, there is a need for integrated and sustainable strategies tailored to Iran’s calcareous soils. Therefore, this study pursued three objectives: (i) characterize contaminated soils in Kerman Province, (ii) isolate indigenous bacterial species with high heavy metal and petroleum hydrocarbon degradation potential, and (iii) evaluate the synergistic effects of NaOH-modified rice husk biochar and these bacteria on pollutant bioavailability, soil microbial activity, and maize growth under greenhouse conditions. By linking soil characterization with remediation trials, this work aims to provide a framework for developing effective, locally adapted approaches to soil remediation.”


**Line-by-line:

Paragraph 5 Sentence 3: I would like to see a citation or example of safe limits of these concentrations.

Response: Thank you for your valuable feedback, to address this we revised and changed the introduction as your suggestion.


*Materials and Methods

**Line-by-line:

Figure 1 caption: Change to “...The figure was designed using Adobe...”

Line 47: should be “follows”

Line 50: Is the 1:1 solution by volume (i.e. v/v) or by weight (w/w)?

Also Line 69, 80

Response: Thank you for your valuable feedback, to address this we revised and changed the Materials and Methods as your suggestion.

*Results

**General:

In my opinion, the headings of the results sections could be altered to summarize the findings of that section. Here are my suggested changes. The first bullet point is a more “finding-focused” whereas the second bullet point is more “method-focused”. I personally prefer results section headers that are finding-focused:

Soil characterization

-Method-focused: Physicochemical and contaminant profile of soils

Bacterial identification

-Finding focused: Indigenous strains display strong remediation potential

-Method-focused: Selection of high-potential bioremediating strains

Physiochemical analysis of biochar

-Microbial modification enhances biochar properties

-Characterization of pristine and microbially modified biochar

Greenhouse experiment

-Biochar–bacteria treatments boost maize growth

-Greenhouse evaluation of maize growth under different treatments

Heavy metal immobilization

-Synergistic biochar-bacteria treatments reduce heavy metal bioavailability

-Assessment of treatment effects on heavy metal immobilization

Petroleum hydrocarbon degradation

-Modified biochar accelerates hydrocarbon breakdown

-Evaluation of petroleum hydrocarbon degradation by treatments

TF and BAF in Maize for heavy metal

-Biochar-bacteria treatments lower heavy metal uptake in maize

-Translocation and bioaccumulation of heavy metals in maize

In the Results section, many numerical values are already presented in tables and figures. I suggest focusing the text on whether the changes were statistically significant and on the size of the change, instead of repeating all the numbers. I have made example suggestions of this in the line-by-line section. However, I would suggest the authors to consider this throughout. You did this well in lines 260-267.


**Line-by-line:

Lines 166-170: I would suggest comparing to the uncontaminated soil

Figure 4 caption: Change to “FT-IR spectrum of pristine biochar (PB) and M-biochar (MB).”

In the manuscript I’ve seen FT-IR and FTIR, try and be consistent

Figure 5 caption: Change to “FESEM image of (a) pristine biochar and (b) M-biochar."

228-239: Make this section clearer. As is, it is difficult to tell when certain treatments did better than the control or just better than the untreated, contaminated control.

Please clarify whether the treatments improved growth only compared to the contaminated soil, or also compared to the uncontaminated control

-Maybe add a sentence summary at the end like this: “While all treatments improved growth compared to the contaminated control, the performance of MB even surpassed that of the uncontaminated soil. The superior performance of MB suggests synergistic effects between biochar’s adsorption capacity and bacterial degradation, fostering a favorable environment for plant growth (p < 0.01; Table 4).”

230: isn’t this missing a treatment? You talk about “Bacterial inoculants” later in the paragraph, but that treatment is not listed in this sentence.

246: no detectable metals for the uncontaminated soil. However, Figure 6 and the tables show value for the uncontaminated soils, please check/clarify. Are they below detection limit?

245-254: Could be improved to enhance clarity – I suggest altering the results to showcase the percent change rather than listing the actual numbers (you can just cite the Table). This way it is easier for the reader to determine the amount of change:

-“Compared to the contaminated soil, all treatments reduced metal concentrations (Table 4). MB generally achieved the greatest reduction (p < 0.001), lowering Cr, Pb, Cd, and Cu by 40.0 ± Y, 33.3 ± Y, 37.5 ± Y, and 40.0 ± Y%, respectively. Bacterial inoculants decreased these concentrations by 20-27% whereas PB only achieved 16.7-20% reductions. The untreated control showed minimal, insignificant changes (p > 0.05) with concentrations of Cr, Pb, Cd, and Cu concentrations only reducing by 1.7-3% (Table 4).”

-Please do not rely on my quick example calculations, as I did not include errors or standard deviations. If taking my suggestion, please redo all analyses and do not take my statistical assumptions (i.e. retest them).

-Besides MB treatment, only Cr and Pb are compared. I did not see a reason mentioned.

Figure 6: I would like to see some identification of statistical significance. For example an asterix could be added above the bars that are significantly different than the values of the contaminated soil. If this is done, add a description of this in the figure caption. Example: “Asterix (*) show values are significantly different (p < 0.05) than the contaminated soil.”

Line 265: “the control” can be confusing since you have multiple (i.e. uncontaminated soil, contaminated soil, and the without treatment). Throughout the results I would specify the control by using something like: “the without treatment control” or the “untreated control”.

Figure 7: The image quality of this figure is low. Please try to improve the resolution for final submission.

Line 269-270: should be “Chromatograms of petroleum hydrocarbon for (a) standard reference of TPH and the following experimental conditions: (b) without treatment, (c) PB, (d) bacterial inoculation, and (e) MB.

Response: Thank you for your valuable feedback, to address this we revised and changed the entire Materials and Methods as your suggestion.

*Discussion

**General:

The Discussion contains many numbers that are already reported in the Results and tables. I suggest summarizing the main findings and citing the appropriate table or figure, instead of restating the numerical values. Example (Lines 294-298): instead of listing the percentage of hydrocarbon degradation and heavy metal removal from each you could add a range for all and cite the figure.

-“with five isolates selected for their superior bioremediation potential (60-85% petroleum hydrocarbon degradation, 60-80% heavy metal removal): P. fluorescens, R. qingshengii, B. metallica, B. cereus, and S. pactum (Figure 2).”

I suggest adding clear subsections in the Discussion to separate the parts about hydrocarbon degradation/removal and the parts about plant growth. These are major findings that deserve more emphasis:

Header suggestions:

-Between Lines 291 and 292: Synergistic effects of native bacterial consortia and biochar on soil remediation

-Between Lines 341 and 342: Enhanced maize growth and reduced metal uptake under combined treatments

-Between Lines 386 and 387: Implications, limitations, and future directions for sustainable soil remediation

**Line-by-line:

Lines 312-330: very results heavy

Response Thank you for your continued feedback on the manuscript, we fully revised discussion section as your suggestion.
