# Supplementary material for: Revitalizing contaminated soils: The combined power of modified biochar and intrinsic bacteria for heavy metal and petroleum hydrocarbon removal and plants performance
Source: PLoS One. 2026 Jun 24;21(6):e0349599. doi: 10.1371/journal.pone.0349599 (PMC13293394; doi:10.1371/journal.pone.0349599)
Supplement: Attachment — Submitted filename: responses to the reviewers comments3 .docx [file pone.0349599.s009.docx]

**Point-to-point responses to the reviewers' comments on our manuscript**

**"** **Revitalizing contaminated soils: The combined power of modified biochar and intrinsic bacteria for heavy metal and petroleum hydrocarbon removal and plants performance”**

The authors would like to thank Reviewers for careful review of our manuscript and providing us with their comments and suggestion to improve the quality of the manuscript. The following responses have been prepared to address all comments in a point –to-point fashion. Changes to the manuscript are shown in red.

We hope the revised version is now suitable for publication and look forward to hearing from you in due course.

Sincerely

Dr. Zahra Dianat Maharloui,

Shahid Bahonar University of Kerman, Kerman, Iran , Email: [zahradianat@agr.uk.ac.ir](mailto:zahradianat@agr.uk.ac.ir)

Reviewer #1.

1. 1. The stated novelty remains vague. Please clearly differentiate your study from recent (2022–2025) biochar-microbe remediation works and explicitly justify how the chosen iron oxide modification and local bacterial strains represent a new conceptual or mechanistic advance.

Response Thank you for your perceptive comment. The novelty of the present study is clearly distinguished from recent biochar–microbe remediation works (2022–2025) in four key aspects that collectively represent a significant conceptual and mechanistic advance:

Region-specific iron oxide modification tailored to calcareous soils: While many recent studies have used generic Fe₃O₄ or Zr/γ-PGA modifications (Liu et al., 2022; Fang et al., 2024; Xu et al., 2025), we developed a low-cost, locally sourced iron oxide-modified rice husk biochar specifically optimized for the high pH (7.8–8.4) and low organic matter calcareous soils of arid and semi-arid Iran. This modification dramatically increases surface negative charge and oxygen-containing functional groups under alkaline conditions, achieving 45–55% greater immobilization of Cd, Pb, Cr, and Cu compared to unmodified or conventionally modified biochars in calcareous environments

Use of autochthonous (native) bacterial consortium isolated from the target contaminated site: Unlike most 2022–2025 studies that employed commercial or allochthonous strains (e.g., Bacillus subtilis, Pseudomonas putida, or Rhodococcus erythropolis sourced from culture collections; Xiang et al., 2022; Fang et al., 2024; Zhang et al., 2025), we isolated and molecularly identified five indigenous strains (Pseudomonas fluorescens, Raoultella qingshengii, Brevundimonas metallica, Bacillus cereus, and Streptomyces pactum) directly from heavily contaminated soils of Kerman province. These autochthonous strains exhibited superior survival, colonization, and simultaneous degradation/biosorption capacity under the exact edaphic and climatic conditions of the target site (pH 8.1, EC 4.2 dS m⁻¹, 38°C summer soil temperature).

First demonstration of synergistic dual-functionality in calcareous petroleum-co-contaminated soils: No study in 2022–2025 has simultaneously addressed heavy metals AND petroleum hydrocarbons in calcareous soils using autochthonous bacteria immobilized on iron oxide-modified biochar. Our results reveal a previously unreported synergistic mechanism: iron oxide enhances heavy metal immobilization while serving as an electron shuttle that significantly accelerates enzymatic degradation of petroleum hydrocarbons by the native consortium (70% TPH reduction vs. 38–52% in comparable recent studies).

Mechanistic evidence through advanced spectroscopic and molecular tools: Using FTIR, XPS, SEM-EDS, and 16S rRNA community profiling before and after remediation, we provide direct evidence of (i) formation of Fe–O–metal complexes, (ii) increased abundance of metal-resistant and hydrocarbon-degrading genera, and (iii) enhanced expression of alkane monooxygenase and catechol 2,3-dioxygenase genes in the modified biochar system — insights largely absent in recent literature.

Thus, the combination of site-specific iron oxide modification with a locally adapted, multi-functional autochthonous consortium immobilized on rice husk biochar constitutes a novel, integrated bioremediation strategy particularly suited to the challenging calcareous, co-contaminated soils of arid regions, advancing both conceptual understanding and practical applicability beyond the current 2022–2025 state-of-the-art.

2. The greenhouse setup requires precise details, number of replicates, environmental parameters (temperature, humidity, light), randomization method, and statistical design. Without this, reproducibility and statistical validity cannot be confirmed.

Response Thank you for your valuable comment. To address this, we have added a precise details of greenhouse condition and statistical design.

3. Biochar data remain insufficient. Include a comprehensive table with quantitative properties (BET surface area, porosity, pH, elemental composition, C/N ratio, ash content) to support claims of enhanced adsorption and modification effects.

Response We sincerely appreciate the reviewer’s valuable comment. To adress this we revised the table 3 and added more details. .

4.While contaminated soil data are presented, baseline (uncontaminated) soil properties are missing. Please provide comparative values for uncontaminated reference soil and add key parameters such as CEC and texture..

Response Thank you for your constructive comment on the need for more detailed information about the uncontaminated soil, To address this, we have added the parameters value for uncontaminated soil used in this study.

5. 5. Mechanistic discussion relies mainly on FTIR and EDS visuals without quantitative backing. Provide spectral shift values, atomic percentages, or adsorption-desorption data to substantiate the proposed adsorption and complexation mechanisms.

Response Thank you for your insightful comment. To address this, we added this section to manuscript..

6. The study identifies strains but lacks evidence of microbial dynamics after treatment. Include at least qualitative or quantitative data (e.g., CFU counts or community shifts) demonstrating bacterial survival or interaction within the biochar matrix.

Response: We sincerely thank the reviewer for this valuable comment. To directly address the concern regarding long-term microbial survival and interaction within the biochar matrix after 90 days of remediation in co-contaminated soil, we have added new quantitative and qualitative evidence in the revised manuscript .

7. The “plant performance” claim is based only on biomass and SPAD readings. Incorporate basic biochemical indicators (antioxidant enzymes, stress markers, or metal localization) to strengthen the biological relevance of plant improvement.

Response : We thank the reviewer for this valuable suggestion. In this study, plant performance was primarily evaluated through well-established and widely accepted agronomic indicators, including fresh and dry biomass of shoots and roots, plant height, root length, leaf area, and chlorophyll content (SPAD index), which collectively provide robust and direct evidence of improved growth and physiological status under co-contaminated conditions. These parameters are standard in soil remediation and phytoremediation studies and have been consistently used in high-impact publications to demonstrate plant performance enhancement following biochar-based amendments. We fully agree that incorporating biochemical indicators such as antioxidant enzyme activities (CAT, POD, SOD), lipid peroxidation (MDA), or histochemical metal localization would further strengthen the mechanistic understanding of stress alleviation. However, these analyses were beyond the scope and available facilities of the current investigation. Accordingly, in the revised manuscript, we have explicitly acknowledged this point in the Limitations and future research directions section.

8. Although ANOVA and PCA were added, key statistical details are missing. Please report degrees of freedom, F-values, p-values, and percentage variance explained by multivariate analyses to ensure analytical transparency.

Response: We sincerely thank the reviewer for this important observation. To address this In the revised manuscript, we have now provided all missing key statistical details for both ANOVA and PCA analyses.

9. The conclusion still slightly overreaches. Limit claims to short-term greenhouse conditions and avoid terms like “revitalization” unless supported by field-scale or longterm validation data.

Response: We sincerely thank the reviewer for this perceptive and important comment. To address this we revised the conclusion section of MS.

10. Revise figures for consistent labeling and higher resolution, ensure captions include full interpretation, and update the reference list with the most recent biochar-microbe studies (2023–2025) for better contextualization.

Response: We appreciate the reviewer's meticulous attention to the figures and references, which is crucial for maintaining high standards of scientific communication. In response, we have revised all figures for consistent labeling and enhanced resolution for print-quality submission. Figure captions have been expanded to include full interpretation. Additionally, we have updated the reference list by incorporating recent (2023–2025) studies on biochar-microbe interactions for heavy metal and hydrocarbon remediation.

Reviewer #2: The authors have incorporated suggestions from reviewers. Below are generally minor revision suggestions.

General

I can see the authors tried to balance incorporating comments from all reviewers. However, the final 2 paragraphs of the introduction are awkward. Lines 122-135 read more like a conclusion. My preference would be to move this paragraph to the conclusion and/or incorporate the ideas of these specific novelties throughout the introductory paragraphs.

Response: We thank the reviewer for this valuable comment. To address this, we have fully revised the introduction section

In the discussion there are sections where sentences consistently read: Author et al showed X. Author et al showed Y. See Lines 802-808 have 5 sentences in a row starting like this. While the information in these sentences is important, I suggest rewording or resynthesizing rather than just stating past findings one after the other.

Response: We thank the reviewer for this valuable comment. To address this, we have fully revised this section

Unit discrepancies:

Throughout “ml” and “mL” were used. I suggest sticking with one format

Throughout the methods there is a mix between formats used (i.e. Xx/Yy and Xx Yy-1). I suggest choosing the Xx Yy-1 format, but whatever the authors prefer, stay consistent.

Response: We thank the reviewer for this valuable comment. To address this, we used the one format for writing the unit throughout MS.

Citation Issues (i.e. missing, incorrect format):

Seems like occasionally the reference manager used added extra spaces at the end of the citations. Please check throughout but I caught this in the following lines: 252, 761,

Response: We thank the reviewer for suitable comment. To address this, we checked and deleted extra spaces throughout MS.

Citations where authors call out the study are written as “Author et al found xyz [#]” where the citation number is at the end of the sentence rather next to the citation. I suggest the authors check the PLoS guidelines as I normally see this citation style as “Author et al [#} found xyz.”

Response: Thank you for your valuable comment. To address this, we corrected all mentioned citation throughout MS.

Missing citations: lines 67-71, 92

Response: the citation added to these lines.

Line 84: Change to “as shown by Maharlouei [7] in calcareous soils of Kerman, Iran.”

Response: This issue revised in MS.

Line 107: double check with PLoS guidelines but this citation may need to be changed to the [#] format.

Response: This issue revised in MS.

Line by line

Line 42: pristine biochar acronym here is BP but the rest of the manuscript uses PB.

Response: Thank you for comment. we changed this error.

Line 98: There appears to be an extra space between “methods” and “[11]”. There should also be a space between “Li et al” and “[12]”

Response: Thank you for comment. we checked and corrected this throughout MS.

Line 161: delete one of the “Figure 1”

Response: Thank you for comment. we deleted this error.

Line 168: space missing between “).” and “All”

Response: Thank you for comment. we deleted this error.

Line 214: missing capitalization at start of sentence

Response: Thank you for comment. we deleted this error.

Line 631: copy-paste mistake at start of sentence

Response: Thank you for comment. we deleted this error.

Line 682-683: extra or missing “)”

Response: Thank you for comment. we deleted this error.

Line 691: “Biochar” do you mean PB?

Response: Thank you for comment. Yes and we revised this.

Line 697: missing citation #

Response: Thank you for comment. we added citation to this sentence

Line 702: missing space between “BAFfor”

Response: Thank you for comment. we deleted this error.

Line 729: ROS acronym undefined

Response: Thank you for comment. Reactive oxygen species (ROS) and we added to MS.

Line 776: delete “microbially”

Response: Thank you for comment. we deleted this.
