# Supplementary material for: Revitalizing contaminated soils: The combined power of modified biochar and intrinsic bacteria for heavy metal and petroleum hydrocarbon removal and plants performance
Source: PLoS One. 2026 Jun 24;21(6):e0349599. doi: 10.1371/journal.pone.0349599 (PMC13293394; doi:10.1371/journal.pone.0349599)
Supplement: Attachment — Submitted filename: responses to the reviewers comments4 .docx [file pone.0349599.s010.docx]

**Point-to-point responses to the reviewers' comments on our manuscript**

**"** **Revitalizing contaminated soils: The combined power of modified biochar and intrinsic bacteria for heavy metal and petroleum hydrocarbon removal and plants performance”**

The authors would like to thank Reviewers for careful review of our manuscript and providing us with their comments and suggestion to improve the quality of the manuscript. The following responses have been prepared to address all comments in a point –to-point fashion. Changes to the manuscript are shown in red.

We hope the revised version is now suitable for publication and look forward to hearing from you in due course.

Sincerely

Dr. Zahra Dianat Maharloui,

Shahid Bahonar University of Kerman, Kerman, Iran , Email: [zahradianat@agr.uk.ac.ir](mailto:zahradianat@agr.uk.ac.ir)

**Reviewer comments:**

Reviewer #1:

The manuscript has improved substantially and now presents a coherent and well-supported study. Before final acceptance, please address the following minor but necessary points:

(i) include a brief supplementary example or summary table illustrating a representative calculation (e.g., contaminant reduction or index derivation) to further enhance transparency;

Response: We thank the reviewer for this valuable and constructive suggestion. To enhance transparency and facilitate reproducibility, we have added a concise summary table illustrating a representative calculation of contaminant reduction and index derivation (TF and BAF), Plant growth & physiological indices, petroleum hydrocarbon degradation efficiency and heavy metal reduction efficiency in soil under different treatments. The tables have been included in the Supplementary Information section and is explicitly referenced in the results section.

(ii) (ii) perform a final consistency check of all figures and tables to ensure uniform labeling, units, and formatting throughout; and

Response: We appreciate the reviewer’s careful attention to consistency and presentation quality. A comprehensive final consistency check has been performed for all figures and tables. Labeling, units, abbreviations, numerical precision, and formatting have been standardized throughout the manuscript.

(iii) (iii) carefully proofread the manuscript for minor language, typographical, and formatting inconsistencies. In particular, revise the text to reduce overly generic or repetitive phrasing (e.g., frequent use of terms such as robust, underscores, overall), and standardize punctuation and hyphenation, avoiding excessive or stylistically inconsistent use of em/en dashes, so that the manuscript reads as carefully authored, original scientific prose.

Response: We thank the reviewer for this insightful and constructive comment. The manuscript has been carefully and thoroughly proofread to address minor language, typographical, and formatting inconsistencies.

Reviewer #2: There were inconsistencies between the manuscript and the tracked-changes manuscript. I apologize if my comments do not reflect the final work.

Response: Thank you for noting this issue. The inconsistencies resulted from the upload of an earlier manuscript version, while revised sections were shown in the tracked-changes file. These sections have now been fully incorporated, and the current manuscript represents the final corrected version. We apologize for any confusion.

Overall figures were blurry, ensure on final submission they are high resolution

Line 160: Figure 1 is repeated

Response: We thank the reviewer for noting these issues. All figures have been carefully checked and replaced with high-resolution versions (minimum 300 dpi) to ensure clarity in the final submission. In addition, the repeated appearance of Figure 1 at line 160 was an unintentional duplication and has now been corrected. The figure numbering and in-text references have been reviewed and are fully consistent in the revised manuscript.

Figure 9 needs to be reformatted and have a consistent lettering system. Each panel needs to be its own. For example C) currently shows multiple panels. Each of those images should have its own label (i.e., d), e), f), etc). Also this figure incorporates a table. I would look at PLOS's standards to make sure that is okay. You likely will have to separate them.

Response: Figure 9 has been reformatted to ensure a consistent lettering system. Each panel is now presented as an individual image with a unique label (a–g). In addition, the EDS elemental analysis table has been separated from the figure and presented as an independent table in accordance with PLOS formatting guidelines.

Figure 10: there is a white box near the legend, this figure should be remade. Also why are the x-axis units incorrect? There is no consistency. Something has to be wrong here.

Response: Thank you for your valuable comment. To address this, we corrected and redesigned the figure 10.
