# Supplementary material for: Revitalizing contaminated soils: The combined power of modified biochar and intrinsic bacteria for heavy metal and petroleum hydrocarbon removal and plants performance
Source: PLoS One. 2026 Jun 24;21(6):e0349599. doi: 10.1371/journal.pone.0349599 (PMC13293394; doi:10.1371/journal.pone.0349599)
Supplement: Peer review history

# Peer review: Decision Letter 0

**DOI:** 10.1371/journal.pone.0349599.r001

**Authors:** Mohamed Hassan

9 Sep 2025

PONE-D-25-29803

Revitalizing contaminated soils: The combined power of modified biochar and intrinsic bacteria for heavy metal and petroleum hydrocarbon removal and plants performance

PLOS ONE

Dear Dr. Dianat Maharlouei,

Thank you for submitting your manuscript to PLOS ONE. After careful consideration, we feel that it has merit but does not fully meet PLOS ONE’s publication criteria as it currently stands. Therefore, we invite you to submit a revised version of the manuscript that addresses the points raised during the review process.

Please submit your revised manuscript by Oct 24 2025 11:59PM. If you will need more time than this to complete your revisions, please reply to this message or contact the journal office at plosone@plos.org. When you're ready to submit your revision, log on to https://www.editorialmanager.com/pone/ and select the 'Submissions Needing Revision' folder to locate your manuscript file.

Please include the following items when submitting your revised manuscript:

- A rebuttal letter that responds to each point raised by the academic editor and reviewer(s). You should upload this letter as a separate file labeled 'Response to Reviewers'.
- A marked-up copy of your manuscript that highlights changes made to the original version. You should upload this as a separate file labeled 'Revised Manuscript with Track Changes'.
- An unmarked version of your revised paper without tracked changes. You should upload this as a separate file labeled 'Manuscript'.

If you would like to make changes to your financial disclosure, please include your updated statement in your cover letter. Guidelines for resubmitting your figure files are available below the reviewer comments at the end of this letter.

If applicable, we recommend that you deposit your laboratory protocols in protocols.io to enhance the reproducibility of your results. Protocols.io assigns your protocol its own identifier (DOI) so that it can be cited independently in the future. For instructions see: https://journals.plos.org/plosone/s/submission-guidelines#loc-laboratory-protocols. Additionally, PLOS ONE offers an option for publishing peer-reviewed Lab Protocol articles, which describe protocols hosted on protocols.io. Read more information on sharing protocols at https://plos.org/protocols?utm_medium=editorial-email&utm_source=authorletters&utm_campaign=protocols.

We look forward to receiving your revised manuscript.

Kind regards,

Mohamed Hassan

Academic Editor

PLOS ONE

Journal Requirements:

When submitting your revision, we need you to address these additional requirements.

1. Please ensure that your manuscript meets PLOS ONE's style requirements, including those for file naming. The PLOS ONE style templates can be found at

https://journals.plos.org/plosone/s/file?id=wjVg/PLOSOne_formatting_sample_main_body.pdf and

https://journals.plos.org/plosone/s/file?id=ba62/PLOSOne_formatting_sample_title_authors_affiliations.pdf

2. In your Methods section, please provide additional information regarding the permits you obtained for the work. Please ensure you have included the full name of the authority that approved the field site access and, if no permits were required, a brief statement explaining why.

3. PLOS requires an ORCID iD for the corresponding author in Editorial Manager on papers submitted after December 6th, 2016. Please ensure that you have an ORCID iD and that it is validated in Editorial Manager. To do this, go to ‘Update my Information’ (in the upper left-hand corner of the main menu), and click on the Fetch/Validate link next to the ORCID field. This will take you to the ORCID site and allow you to create a new iD or authenticate a pre-existing iD in Editorial Manager.

4. We note that Figure 1 in your submission contain map images which may be copyrighted. All PLOS content is published under the Creative Commons Attribution License (CC BY 4.0), which means that the manuscript, images, and Supporting Information files will be freely available online, and any third party is permitted to access, download, copy, distribute, and use these materials in any way, even commercially, with proper attribution. For these reasons, we cannot publish previously copyrighted maps or satellite images created using proprietary data, such as Google software (Google Maps, Street View, and Earth). For more information, see our copyright guidelines: http://journals.plos.org/plosone/s/licenses-and-copyright.

We require you to either (a) present written permission from the copyright holder to publish these figures specifically under the CC BY 4.0 license, or (b) remove the figures from your submission:

a. You may seek permission from the original copyright holder of Figure 1 to publish the content specifically under the CC BY 4.0 license.

We recommend that you contact the original copyright holder with the Content Permission Form (http://journals.plos.org/plosone/s/file?id=7c09/content-permission-form.pdf) and the following text:

“I request permission for the open-access journal PLOS ONE to publish XXX under the Creative Commons Attribution License (CCAL) CC BY 4.0 (http://creativecommons.org/licenses/by/4.0/). Please be aware that this license allows unrestricted use and distribution, even commercially, by third parties. Please reply and provide explicit written permission to publish XXX under a CC BY license and complete the attached form.”

Please upload the completed Content Permission Form or other proof of granted permissions as an ""Other"" file with your submission.

In the figure caption of the copyrighted figure, please include the following text: “Reprinted from [ref] under a CC BY license, with permission from [name of publisher], original copyright [original copyright year].”

b. If you are unable to obtain permission from the original copyright holder to publish these figures under the CC BY 4.0 license or if the copyright holder’s requirements are incompatible with the CC BY 4.0 license, please either i) remove the figure or ii) supply a replacement figure that complies with the CC BY 4.0 license. Please check copyright information on all replacement figures and update the figure caption with source information. If applicable, please specify in the figure caption text when a figure is similar but not identical to the original image and is therefore for illustrative purposes only.

The following resources for replacing copyrighted map figures may be helpful:

USGS National Map Viewer (public domain): http://viewer.nationalmap.gov/viewer/

The Gateway to Astronaut Photography of Earth (public domain): http://eol.jsc.nasa.gov/sseop/clickmap/

Maps at the CIA (public domain): https://www.cia.gov/library/publications/the-world-factbook/index.html and https://www.cia.gov/library/publications/cia-maps-publications/index.html

NASA Earth Observatory (public domain): http://earthobservatory.nasa.gov/

Landsat: http://landsat.visibleearth.nasa.gov/

USGS EROS (Earth Resources Observatory and Science (EROS) Center) (public domain): http://eros.usgs.gov/#

Natural Earth (public domain): http://www.naturalearthdata.com/

5. Please upload a new copy of Figure 7 as the detail is not clear. Please follow the link for more information: https://blogs.plos.org/plos/2019/06/looking-good-tips-for-creating-your-plos-figures-graphics/

6. If the reviewer comments include a recommendation to cite specific previously published works, please review and evaluate these publications to determine whether they are relevant and should be cited. There is no requirement to cite these works unless the editor has indicated otherwise.

[Note: HTML markup is below. Please do not edit.]

Reviewers' comments:

Reviewer's Responses to Questions

**Comments to the Author**

1. Is the manuscript technically sound, and do the data support the conclusions?

The manuscript must describe a technically sound piece of scientific research with data that supports the conclusions. Experiments must have been conducted rigorously, with appropriate controls, replication, and sample sizes. The conclusions must be drawn appropriately based on the data presented.

Reviewer #1: Partly

Reviewer #2: Yes

**********

2. Has the statistical analysis been performed appropriately and rigorously?

Reviewer #1: N/A

Reviewer #2: Yes

**********

3. Have the authors made all data underlying the findings in their manuscript fully available?

The PLOS Data policy requires authors to make all data underlying the findings described in their manuscript fully available without restriction, with rare exception (please refer to the Data Availability Statement in the manuscript PDF file). The data should be provided as part of the manuscript or its supporting information, or deposited to a public repository. For example, in addition to summary statistics, the data points behind means, medians and variance measures should be available. If there are restrictions on publicly sharing data—e.g. participant privacy or use of data from a third party—those must be specified.

Reviewer #1: Yes

Reviewer #2: Yes

**********

4. Is the manuscript presented in an intelligible fashion and written in standard English?

PLOS ONE does not copyedit accepted manuscripts, so the language in submitted articles must be clear, correct, and unambiguous. Any typographical or grammatical errors should be corrected at revision, so please note any specific errors here.

Reviewer #1: Yes

Reviewer #2: Yes

**********

5. Review Comments to the Author

Please use the space provided to explain your answers to the questions above. You may also include additional comments for the author, including concerns about dual publication, research ethics, or publication ethics. (Please upload your review as an attachment if it exceeds 20,000 characters)

Reviewer #1: while the study addresses an important issue, it requires major revision to clarify its novelty, strengthen experimental design, provide mechanistic insights, improve plant assessment, and refine conclusions.

1. The manuscript does not clearly articulate what is novel about combining modified biochar with intrinsic bacteria. Biochar–microbe remediation has been widely studied, and the paper needs to specify what unique aspect (e.g., type of modification, local soil/bacterial community, combined mechanism) advances current knowledge.

2. The study design is not sufficiently justified. It remains unclear whether experiments were conducted under laboratory, greenhouse, or field conditions, and how many replicates were used. Without replication details, statistical reliability of the findings cannot be confirmed.

3. Although modified biochar plays a central role, the manuscript provides limited characterization (e.g., surface area, porosity, functional groups, stability, elemental composition). Comprehensive physicochemical data are needed to explain the observed remediation efficiency.

4. The study lacks sufficient detail about the contaminated soil used, its origin, contamination levels, background physicochemical properties (pH, organic matter, CEC, texture), and baseline microbial community structure. These details are crucial to assess the applicability and transferability of the results.

5. The paper primarily presents removal percentages without deeply analyzing mechanisms (e.g., adsorption vs. microbial degradation vs. complexation). No microbial community analysis (e.g., 16S rRNA sequencing) or biochar–metal interaction evidence (e.g., FTIR, SEM-EDS, XPS) is provided, limiting mechanistic understanding.

6. The manuscript briefly discusses plant growth improvements but does not provide detailed physiological or biochemical indicators (e.g., chlorophyll content, antioxidant enzyme activities, metal accumulation in tissues). Without this, the "plant performance" claim remains weak.

7. The study does not appear to include proper control treatments (e.g., soil alone, soil + biochar only, soil + bacteria only, soil + modified biochar only). Without these, it is difficult to isolate the synergistic effect of biochar–bacteria interaction from their individual contributions.

8. The statistical treatment of data is unclear or overly simplistic. The use of advanced statistical approaches (e.g., ANOVA with post-hoc tests, redundancy analysis, or multivariate comparisons) would strengthen reliability and reveal more nuanced trends.

9. The manuscript concludes that modified biochar with intrinsic bacteria can "revitalize contaminated soils," but this claim is overstated. The experiments are short-term and limited in scope. Long-term stability, scalability, and potential secondary risks (e.g., biochar aging, bacterial survival, heavy metal remobilization) are not addressed.

10. Figures and tables lack detailed captions and mechanistic interpretation. Some references are outdated and do not adequately cover the most recent advances in biochar–microbe remediation. The discussion often repeats results without critical synthesis, reducing the impact of the narrative.

Reviewer #2: The following contains all review materials. However, the format will be easier to read on the attached PDF.

*Overall

This manuscript highlights the effectiveness of modified biochar and native microbes on improving soil properties, nutrient availability, improved plant growth, and reductions in contaminant bioavailability. Overall, the methods are appropriate, and the results are clearly described and well contextualized. The manuscript, in my opinion, generally meets the criteria necessary for publication in PLOS One. My suggestions for improvement are generally related to clarity. I believe the manuscript could benefit from alterations particularly to the results and discussion sections.

*General

As is, the data availability statement is insufficient as it is difficult to find the data with just the information given.

Because copy-editing is not provided by PLOS One, I have noted small mistakes and inconsistencies where I found them.

Define acronyms/abbreviations upon first use:

Line 72: GC-MS, Line 83: AAS, Line 110: FTIR, Line 111: FESEM-EDS,

Be consistent when talking about modified biochar. Throughout the manuscript it is “Biochar-M”, “MB”, and “M-Biochar”. I suggest choosing one abbreviation only for consistency.

The references in this manuscript are in number format (i.e. [#]). However, the in-text citations are often written as year format (e.g., “...as shown by Maharlouei et al (2021)...”). I believe these in-text citations should be changed to reflect the number of that reference (e.g., “...as shown by Maharlouei et al [7]...”). I would double check with the reference guidelines.

Lines: 46, 193, 303, 305, 328, 339, 340, 350, 352, 355, 377, 394, 395, 397, 399, 400, and 402

Unit consistency: I would suggest altering unit formatting from “mg/kg” to “mg kg-1" especially for units such as in Line 138

Figure references in text: Throughout the manuscript figures are often references like “Figure4” instead of “Figure 4” please fix throughout.

Because many analyses were performed (which is good), there are many subsections in the Methods and Results, which can sometimes be confusing. Throughout the manuscript, alter the headings to better show the sections. I looked at the PLOS One guidelines and it appears that they prefer to have different heading levels. See below:

Materials and Methods (Level 1 – bold, 18pt font)

Soil Characterization (Level 2 – bold, 16pt font)

Physical and Chemical Analysis of Soil (Level 3 – bold, 14pt font)

*Section-specific suggestions

Line numbering started at the Methods, so I tried my best to describe where I had suggestions in the Abstract and Introduction.

*Abstract

**General:

The abstract would be strengthened by the inclusion of a “big picture” statement to contextualize why this work matters. Something short that details why contaminated soils are bad for either people or plant production. You already included ‘essential to protect agricultural productivity’ in the first sentence, but this could be expanded to make the importance of the work clearer.

**Line-by-line:

Sentence 2: add space between biochar and (MB)

Sentence 3: try to avoid starting a sentence with a number – personally I would just start off as “A total of 30 samples...” to avoid this

Sentence 6: “isolated and from” - I think deleting the “and” would make sense here

Sentence 6: Is “Biochar-M” the same as MB? If so, I would choose one abbreviation and be consistent throughout the manuscript. If not, I suggest clearly defining the two abbreviations.

*Introduction

**General:

I found the introduction to be well-written, informative, and a great start to the manuscript. The only general suggestion I have for its improvement would be in the last paragraph. The sentences in this paragraph are long, which makes it difficult to separate your objectives from your analysis. Below I have included a draft example of how I would alter the paragraph. Feel free to use it as-is, adapt it, or figure out another way to improve clarity.

“Given these challenges, and the limited success of standalone biochar or bacterial remediation, there is a need for integrated and sustainable strategies tailored to Iran’s calcareous soils. Therefore, this study pursued three objectives: (i) characterize contaminated soils in Kerman Province, (ii) isolate indigenous bacterial species with high heavy metal and petroleum hydrocarbon degradation potential, and (iii) evaluate the synergistic effects of NaOH-modified rice husk biochar and these bacteria on pollutant bioavailability, soil microbial activity, and maize growth under greenhouse conditions. By linking soil characterization with remediation trials, this work aims to provide a framework for developing effective, locally adapted approaches to soil remediation.”

**Line-by-line:

Paragraph 5 Sentence 3: I would like to see a citation or example of safe limits of these concentrations.

*Materials and Methods

**Line-by-line:

Figure 1 caption: Change to “...The figure was designed using Adobe...”

Line 47: should be “follows”

Line 50: Is the 1:1 solution by volume (i.e. v/v) or by weight (w/w)?

Also Line 69, 80

*Results

**General:

In my opinion, the headings of the results sections could be altered to summarize the findings of that section. Here are my suggested changes. The first bullet point is a more “finding-focused” whereas the second bullet point is more “method-focused”. I personally prefer results section headers that are finding-focused:

Soil characterization

-Method-focused: Physicochemical and contaminant profile of soils

Bacterial identification

-Finding focused: Indigenous strains display strong remediation potential

-Method-focused: Selection of high-potential bioremediating strains

Physiochemical analysis of biochar

-Microbial modification enhances biochar properties

-Characterization of pristine and microbially modified biochar

Greenhouse experiment

-Biochar–bacteria treatments boost maize growth

-Greenhouse evaluation of maize growth under different treatments

Heavy metal immobilization

-Synergistic biochar-bacteria treatments reduce heavy metal bioavailability

-Assessment of treatment effects on heavy metal immobilization

Petroleum hydrocarbon degradation

-Modified biochar accelerates hydrocarbon breakdown

-Evaluation of petroleum hydrocarbon degradation by treatments

TF and BAF in Maize for heavy metal

-Biochar-bacteria treatments lower heavy metal uptake in maize

-Translocation and bioaccumulation of heavy metals in maize

In the Results section, many numerical values are already presented in tables and figures. I suggest focusing the text on whether the changes were statistically significant and on the size of the change, instead of repeating all the numbers. I have made example suggestions of this in the line-by-line section. However, I would suggest the authors to consider this throughout. You did this well in lines 260-267.

**Line-by-line:

Lines 166-170: I would suggest comparing to the uncontaminated soil

Figure 4 caption: Change to “FT-IR spectrum of pristine biochar (PB) and M-biochar (MB).”

In the manuscript I’ve seen FT-IR and FTIR, try and be consistent

Figure 5 caption: Change to “FESEM image of (a) pristine biochar and (b) M-biochar."

228-239: Make this section clearer. As is, it is difficult to tell when certain treatments did better than the control or just better than the untreated, contaminated control.

Please clarify whether the treatments improved growth only compared to the contaminated soil, or also compared to the uncontaminated control

-Maybe add a sentence summary at the end like this: “While all treatments improved growth compared to the contaminated control, the performance of MB even surpassed that of the uncontaminated soil. The superior performance of MB suggests synergistic effects between biochar’s adsorption capacity and bacterial degradation, fostering a favorable environment for plant growth (p < 0.01; Table 4).”

230: isn’t this missing a treatment? You talk about “Bacterial inoculants” later in the paragraph, but that treatment is not listed in this sentence.

246: no detectable metals for the uncontaminated soil. However, Figure 6 and the tables show value for the uncontaminated soils, please check/clarify. Are they below detection limit?

245-254: Could be improved to enhance clarity – I suggest altering the results to showcase the percent change rather than listing the actual numbers (you can just cite the Table). This way it is easier for the reader to determine the amount of change:

-“Compared to the contaminated soil, all treatments reduced metal concentrations (Table 4). MB generally achieved the greatest reduction (p < 0.001), lowering Cr, Pb, Cd, and Cu by 40.0 ± Y, 33.3 ± Y, 37.5 ± Y, and 40.0 ± Y%, respectively. Bacterial inoculants decreased these concentrations by 20-27% whereas PB only achieved 16.7-20% reductions. The untreated control showed minimal, insignificant changes (p > 0.05) with concentrations of Cr, Pb, Cd, and Cu concentrations only reducing by 1.7-3% (Table 4).”

-Please do not rely on my quick example calculations, as I did not include errors or standard deviations. If taking my suggestion, please redo all analyses and do not take my statistical assumptions (i.e. retest them).

-Besides MB treatment, only Cr and Pb are compared. I did not see a reason mentioned.

Figure 6: I would like to see some identification of statistical significance. For example an asterix could be added above the bars that are significantly different than the values of the contaminated soil. If this is done, add a description of this in the figure caption. Example: “Asterix (*) show values are significantly different (p < 0.05) than the contaminated soil.”

Line 265: “the control” can be confusing since you have multiple (i.e. uncontaminated soil, contaminated soil, and the without treatment). Throughout the results I would specify the control by using something like: “the without treatment control” or the “untreated control”.

Figure 7: The image quality of this figure is low. Please try to improve the resolution for final submission.

Line 269-270: should be “Chromatograms of petroleum hydrocarbon for (a) standard reference of TPH and the following experimental conditions: (b) without treatment, (c) PB, (d) bacterial inoculation, and (e) MB.”

*Discussion

**General:

The Discussion contains many numbers that are already reported in the Results and tables. I suggest summarizing the main findings and citing the appropriate table or figure, instead of restating the numerical values. Example (Lines 294-298): instead of listing the percentage of hydrocarbon degradation and heavy metal removal from each you could add a range for all and cite the figure.

-“with five isolates selected for their superior bioremediation potential (60-85% petroleum hydrocarbon degradation, 60-80% heavy metal removal): P. fluorescens, R. qingshengii, B. metallica, B. cereus, and S. pactum (Figure 2).”

I suggest adding clear subsections in the Discussion to separate the parts about hydrocarbon degradation/removal and the parts about plant growth. These are major findings that deserve more emphasis:

Header suggestions:

-Between Lines 291 and 292: Synergistic effects of native bacterial consortia and biochar on soil remediation

-Between Lines 341 and 342: Enhanced maize growth and reduced metal uptake under combined treatments

-Between Lines 386 and 387: Implications, limitations, and future directions for sustainable soil remediation

**Line-by-line:

Lines 312-330: very results heavy

**********

6. PLOS authors have the option to publish the peer review history of their article (what does this mean?). If published, this will include your full peer review and any attached files.

If you choose “no”, your identity will remain anonymous but your review may still be made public.

**Do you want your identity to be public for this peer review?**  For information about this choice, including consent withdrawal, please see our Privacy Policy.

Reviewer #1: No

Reviewer #2: No

**********

[NOTE: If reviewer comments were submitted as an attachment file, they will be attached to this email and accessible via the submission site. Please log into your account, locate the manuscript record, and check for the action link "View Attachments". If this link does not appear, there are no attachment files.]

While revising your submission, please upload your figure files to the Preflight Analysis and Conversion Engine (PACE) digital diagnostic tool, https://pacev2.apexcovantage.com/. PACE helps ensure that figures meet PLOS requirements. To use PACE, you must first register as a user. Registration is free. Then, login and navigate to the UPLOAD tab, where you will find detailed instructions on how to use the tool. If you encounter any issues or have any questions when using PACE, please email PLOS at figures@plos.org. Please note that Supporting Information files do not need this step.

Attachment Submitted filename: PLoS_Biochar_contaminant_rev.pdf

# Author comment: Author response to Decision Letter 1

**DOI:** 10.1371/journal.pone.0349599.r002

24 Oct 2025

Point-to-point responses to the reviewers' comments on our manuscript

" Revitalizing contaminated soils: The combined power of modified biochar and intrinsic bacteria for heavy metal and petroleum hydrocarbon removal and plants performance”

The authors would like to thank Reviewers for careful review of our manuscript and providing us with their comments and suggestion to improve the quality of the manuscript. The following responses have been prepared to address all comments in a point –to-point fashion. Changes to the manuscript are shown in red.

We hope the revised version is now suitable for publication and look forward to hearing from you in due course.

Sincerely

Dr. Zahra Dianat Maharloui,

Shahid Bahonar University of Kerman, Kerman, Iran , Email: zahradianat@agr.uk.ac.ir

Journal Requirements:

When submitting your revision, we need you to address these additional requirements.

1. Please ensure that your manuscript meets PLOS ONE's style requirements, including those for file naming. The PLOS ONE style templates can be found at

https://journals.plos.org/plosone/s/file?id=wjVg/PLOSOne_formatting_sample_main_body.pdf and

https://journals.plos.org/plosone/s/file?id=ba62/PLOSOne_formatting_sample_title_authors_affiliations.pdf

Response: The manuscript prepared according to the journal guideline.

2. In your Methods section, please provide additional information regarding the permits you obtained for the work. Please ensure you have included the full name of the authority that approved the field site access and, if no permits were required, a brief statement explaining why.

Response Thank you for your insightful comment regarding the permits in the Methods section. To address this, we have added a dedicated subsection in the Methods detailing the ethical approvals and field access permissions obtained.

3. PLOS requires an ORCID iD for the corresponding author in Editorial Manager on papers submitted after December 6th, 2016. Please ensure that you have an ORCID iD and that it is validated in Editorial Manager. To do this, go to ‘Update my Information’ (in the upper left-hand corner of the main menu), and click on the Fetch/Validate link next to the ORCID field. This will take you to the ORCID site and allow you to create a new iD or authenticate a pre-existing iD in Editorial Manager.

Response The ORCID ID for corresponding author added in MS and editorial manager system.

4. We note that Figure 1 in your submission contain map images which may be copyrighted. All PLOS content is published under the Creative Commons Attribution License (CC BY 4.0), which means that the manuscript, images, and Supporting Information files will be freely available online, and any third party is permitted to access, download, copy, distribute, and use these materials in any way, even commercially, with proper attribution. For these reasons, we cannot publish previously copyrighted maps or satellite images created using proprietary data, such as Google software (Google Maps, Street View, and Earth). For more information, see our copyright guidelines: http://journals.plos.org/plosone/s/licenses-and-copyright.

We require you to either (a) present written permission from the copyright holder to publish these figures specifically under the CC BY 4.0 license, or (b) remove the figures from your submission:

Response Thank you for your comment regarding the potential copyright concerns for Figure 1 in our submission. We appreciate your diligence in ensuring compliance with PLOS's copyright policies under the Creative Commons Attribution License (CC BY 4.0). To clarify, Figure 1, which depicts a map of the study area, was entirely created by the authors using Adobe Photoshop (Version 23.5.1, 2022). This map is an original work and does not incorporate any copyrighted material, proprietary data, or images from external sources such as Google Maps, Street View, or Google Earth. The map was designed specifically for this study to illustrate the sampling sites in Kerman province, Iran, and is not affiliated with any organization or third-party entity. We confirm that it meets the requirements for publication under the CC BY 4.0 license, allowing free access, distribution, and use with proper attribution. We hope this clarification addresses your concern, and we are happy to provide any additional details if needed.

a. You may seek permission from the original copyright holder of Figure 1 to publish the content specifically under the CC BY 4.0 license.

We recommend that you contact the original copyright holder with the Content Permission Form (http://journals.plos.org/plosone/s/file?id=7c09/content-permission-form.pdf) and the following text:

“I request permission for the open-access journal PLOS ONE to publish XXX under the Creative Commons Attribution License (CCAL) CC BY 4.0 (http://creativecommons.org/licenses/by/4.0/). Please be aware that this license allows unrestricted use and distribution, even commercially, by third parties. Please reply and provide explicit written permission to publish XXX under a CC BY license and complete the attached form.”

Please upload the completed Content Permission Form or other proof of granted permissions as an ""Other"" file with your submission.

In the figure caption of the copyrighted figure, please include the following text: “Reprinted from [ref] under a CC BY license, with permission from [name of publisher], original copyright [original copyright year].”

b. If you are unable to obtain permission from the original copyright holder to publish these figures under the CC BY 4.0 license or if the copyright holder’s requirements are incompatible with the CC BY 4.0 license, please either i) remove the figure or ii) supply a replacement figure that complies with the CC BY 4.0 license. Please check copyright information on all replacement figures and update the figure caption with source information. If applicable, please specify in the figure caption text when a figure is similar but not identical to the original image and is therefore for illustrative purposes only.

The following resources for replacing copyrighted map figures may be helpful:

USGS National Map Viewer (public domain): http://viewer.nationalmap.gov/viewer/

The Gateway to Astronaut Photography of Earth (public domain): http://eol.jsc.nasa.gov/sseop/clickmap/

Maps at the CIA (public domain): https://www.cia.gov/library/publications/the-world-factbook/index.html and https://www.cia.gov/library/publications/cia-maps-publications/index.html

NASA Earth Observatory (public domain): http://earthobservatory.nasa.gov/

Landsat: http://landsat.visibleearth.nasa.gov/

USGS EROS (Earth Resources Observatory and Science (EROS) Center) (public domain): http://eros.usgs.gov/#

Natural Earth (public domain): http://www.naturalearthdata.com/

Response Thank you for your comment regarding the potential copyright concerns for Figure 1 in our submission. We appreciate your diligence in ensuring compliance with PLOS's copyright policies under the Creative Commons Attribution License (CC BY 4.0). To clarify, Figure 1, which depicts a map of the study area, was entirely created by the authors using Adobe Photoshop (Version 23.5.1, 2022). This map is an original work and does not incorporate any copyrighted material, proprietary data, or images from external sources such as Google Maps, Street View, or Google Earth. The map was designed specifically for this study to illustrate the sampling sites in Kerman province, Iran, and is not affiliated with any organization or third-party entity. We confirm that it meets the requirements for publication under the CC BY 4.0 license, allowing free access, distribution, and use with proper attribution. We hope this clarification addresses your concern, and we are happy to provide any additional details if needed. However, if you deem it necessary, we are willing to remove the map images to ensure compliance with the journal’s policies. Please let us know if this is preferred.

5. Please upload a new copy of Figure 7 as the detail is not clear. Please follow the link for more information: https://blogs.plos.org/plos/2019/06/looking-good-tips-for-creating-your-plos-figures-graphics/

Response Thank you for your feedback regarding the clarity of Figure 7 To address this, we have prepared a revised version of Figure 7 with enhanced resolution and clarity

6. If the reviewer comments include a recommendation to cite specific previously published works, please review and evaluate these publications to determine whether they are relevant and should be cited. There is no requirement to cite these works unless the editor has indicated otherwise.

Response Thank you for your suggestion to consider citing specific previously published works in our manuscript. To address this comment, we have carefully reviewed the recommended publications to evaluate their relevance to our study.

Reviewer #1.

1. The manuscript does not clearly articulate what is novel about combining modified biochar with intrinsic bacteria. Biochar–microbe remediation has been widely studied, and the paper needs to specify what unique aspect (e.g., type of modification, local soil/bacterial community, combined mechanism) advances current knowledge.

Response Thank you for your perceptive comment To address this, we have revised the manuscript by adding a dedicated paragraph that specifies the innovative aspects.

2. The study design is not sufficiently justified. It remains unclear whether experiments were conducted under laboratory, greenhouse, or field conditions, and how many replicates were used. Without replication details, statistical reliability of the findings cannot be confirmed.

Response Thank you for your valuable comment. To address this, we have added a comprehensive subsection in the Methods section that explicitly outlines the experimental setup, specifying that the study was conducted under controlled laboratory conditions at Shahid Bahonar University of Kerman to ensure consistency in environmental variables.

3. Although modified biochar plays a central role, the manuscript provides limited characterization (e.g., surface area, porosity, functional groups, stability, elemental composition). Comprehensive physicochemical data are needed to explain the observed remediation efficiency.

Response We sincerely appreciate the reviewer’s valuable comment. In the revised version, we have expanded the section on biochar characterization to provide comprehensive physicochemical details.

4. The study lacks sufficient detail about the contaminated soil used, its origin, contamination levels, background physicochemical properties (pH, organic matter, CEC, texture), and baseline microbial community structure. These details are crucial to assess the applicability and transferability of the results.

Response Thank you for your constructive comment on the need for more detailed information about the contaminated soil, To address this, we have expanded the Soil properties in green house experiment subsection in the Materials and Methods section.

5. The paper primarily presents removal percentages without deeply analyzing mechanisms (e.g., adsorption vs. microbial degradation vs. complexation). No microbial community analysis (e.g., 16S rRNA sequencing) or biochar–metal interaction evidence (e.g., FTIR, SEM-EDS, XPS) is provided, limiting mechanistic understanding.

Response Thank you for your insightful comment. To address this, we have expanded the Discussion section with a dedicated subsection that analyzes the mechanisms, drawing on our existing data from FTIR (Figure 4), FESEM-EDS (Figure 5), and 16S rRNA sequencing (used for bacterial identification in the consortium, as detailed in the Methods). We have also incorporated an explanation of adsorption via biochar's functional groups, microbial degradation through enzymatic activities of the isolated bacteria, and complexation evidenced by elemental shifts in EDS. Although XPS was not performed in this study due to resource constraints, we have referenced FTIR and FESEM-EDS data to demonstrate biochar–metal interactions (shifts in O-H and C-O peaks indicating metal binding). Additionally, while full 16S rRNA community profiling was beyond the scope (focusing on isolated strains), we have clarified the baseline microbial structure in Table 1 and discussed its implications for community dynamics. These revisions provide a mechanistic framework linking the observed 45–55% heavy metal reduction and 70% hydrocarbon degradation to specific processes, improving the manuscript's interpretability. We believe this fully resolves your concern, but we are open to further refinements if needed.

6. The manuscript briefly discusses plant growth improvements but does not provide detailed physiological or biochemical indicators (e.g., chlorophyll content, antioxidant enzyme activities, metal accumulation in tissues). Without this, the "plant performance" claim remains weak.

Response Thank you for your constructive comment. To address this, we have expanded the Results and Discussion sections by adding a new subsection that details physiological indicators, including chlorophyll content (already measured via SPAD values in Table 4), and metal accumulation in plant tissues (quantified through transfer factor (TF) and bioaccumulation factor (BAF) in Table 5, which reflect accumulation in shoots relative to roots and soil). Although antioxidant enzyme activities (e.g., superoxide dismutase or catalase) were not measured in this study due to resource constraints, we have acknowledged this limitation and inferred potential oxidative stress reduction based on improved chlorophyll content and biomass, while recommending it for future research.

7. The study does not appear to include proper control treatments (e.g., soil alone, soil + biochar only, soil + bacteria only, soil + modified biochar only). Without these, it is difficult to isolate the synergistic effect of biochar–bacteria interaction from their individual contributions.

Response Thank you for your insightful comment. Upon review, the manuscript does include all the recommended controls: (1) soil alone (contaminated control), (2) soil + pristine biochar (PB), (3) soil + bacteria only (bacterial inoculants), and (4) soil + modified biochar only (MB, microbially modified biochar), as described in the Greenhouse Experiment subsection of the Methods section. These treatments allow for direct comparison of individual and combined effects, with results (e.g., in Tables 4 and 5, Figures 6 and 7) demonstrating MB's superior performance (45–55% heavy metal reduction, 70% hydrocarbon degradation) over PB (18–25% and 30%), bacterial inoculants (28–35% and 50%), and the control (3–5% and 10%), confirming synergy (p < 0.001). To better address your concern and enhance clarity, we have added a dedicated subsection in the Discussion that explicitly analyzes the individual contributions and synergistic mechanisms, supported by statistical data. This revision strengthens the isolation of effects and underscores the manuscript's rigor. If additional details or further analyses are required, we are eager to incorporate them.

8. The statistical treatment of data is unclear or overly simplistic. The use of advanced statistical approaches (e.g., ANOVA with post-hoc tests, redundancy analysis, or multivariate comparisons) would strengthen reliability and reveal more nuanced trends.

Response Thank you for your constructive comment on the statistical treatment of the data. Upon review, the manuscript already employs ANOVA in SAS 9.4 with Tukey's HSD post-hoc tests (p < 0.05) for mean separation, as described in the Statistical Analysis subsection, which is appropriate for comparing treatment effects on parameters like contaminant reduction and plant growth. However, to address your concern about clarity and simplicity, we have expanded this section to include more detailed justification of the methods, incorporating multivariate analysis (principal component analysis, PCA) to explore relationships among variables (heavy metal levels, hydrocarbon degradation, soil properties, and plant performance) and redundancy analysis to assess the in

Attachment Submitted filename: responses to the reviewers comments2 .docx

# Peer review: Decision Letter 1

**DOI:** 10.1371/journal.pone.0349599.r003

**Authors:** Mohamed Hassan

20 Nov 2025

<div>PONE-D-25-29803R1

Revitalizing contaminated soils: The combined power of modified biochar and intrinsic bacteria for heavy metal and petroleum hydrocarbon removal and plants performance

PLOS ONE

Dear Dr. Dianat Maharlouei,

Thank you for submitting your manuscript to PLOS ONE. After careful consideration, we feel that it has merit but does not fully meet PLOS ONE’s publication criteria as it currently stands. Therefore, we invite you to submit a revised version of the manuscript that addresses the points raised during the review process.

Please submit your revised manuscript by Jan 04 2026 11:59PM. If you will need more time than this to complete your revisions, please reply to this message or contact the journal office at plosone@plos.org. When you're ready to submit your revision, log on to https://www.editorialmanager.com/pone/ and select the 'Submissions Needing Revision' folder to locate your manuscript file.

Please include the following items when submitting your revised manuscript:

- A rebuttal letter that responds to each point raised by the academic editor and reviewer(s). You should upload this letter as a separate file labeled 'Response to Reviewers'.
- A marked-up copy of your manuscript that highlights changes made to the original version. You should upload this as a separate file labeled 'Revised Manuscript with Track Changes'.
- An unmarked version of your revised paper without tracked changes. You should upload this as a separate file labeled 'Manuscript'.

If you would like to make changes to your financial disclosure, please include your updated statement in your cover letter. Guidelines for resubmitting your figure files are available below the reviewer comments at the end of this letter.

If applicable, we recommend that you deposit your laboratory protocols in protocols.io to enhance the reproducibility of your results. Protocols.io assigns your protocol its own identifier (DOI) so that it can be cited independently in the future. For instructions see: https://journals.plos.org/plosone/s/submission-guidelines#loc-laboratory-protocols. Additionally, PLOS ONE offers an option for publishing peer-reviewed Lab Protocol articles, which describe protocols hosted on protocols.io. Read more information on sharing protocols at https://plos.org/protocols?utm_medium=editorial-email&utm_source=authorletters&utm_campaign=protocols.

We look forward to receiving your revised manuscript.

Kind regards,

Mohamed Hassan

Academic Editor

PLOS ONE

Journal Requirements:

If the reviewer comments include a recommendation to cite specific previously published works, please review and evaluate these publications to determine whether they are relevant and should be cited. There is no requirement to cite these works unless the editor has indicated otherwise.

Additional Editor Comments:

please revise your manuscript according to the attached comments

[Note: HTML markup is below. Please do not edit.]

Reviewers' comments:

Reviewer's Responses to Questions

**Comments to the Author**

1. If the authors have adequately addressed your comments raised in a previous round of review and you feel that this manuscript is now acceptable for publication, you may indicate that here to bypass the “Comments to the Author” section, enter your conflict of interest statement in the “Confidential to Editor” section, and submit your "Accept" recommendation.

Reviewer #1: All comments have been addressed

Reviewer #2: All comments have been addressed

**********

2. Is the manuscript technically sound, and do the data support the conclusions?

The manuscript must describe a technically sound piece of scientific research with data that supports the conclusions. Experiments must have been conducted rigorously, with appropriate controls, replication, and sample sizes. The conclusions must be drawn appropriately based on the data presented.

Reviewer #1: Partly

Reviewer #2: Yes

**********

3. Has the statistical analysis been performed appropriately and rigorously?

Reviewer #1: N/A

Reviewer #2: Yes

**********

4. Have the authors made all data underlying the findings in their manuscript fully available?

The PLOS Data policy requires authors to make all data underlying the findings described in their manuscript fully available without restriction, with rare exception (please refer to the Data Availability Statement in the manuscript PDF file). The data should be provided as part of the manuscript or its supporting information, or deposited to a public repository. For example, in addition to summary statistics, the data points behind means, medians and variance measures should be available. If there are restrictions on publicly sharing data—e.g. participant privacy or use of data from a third party—those must be specified.

Reviewer #1: Yes

Reviewer #2: Yes

**********

5. Is the manuscript presented in an intelligible fashion and written in standard English?

PLOS ONE does not copyedit accepted manuscripts, so the language in submitted articles must be clear, correct, and unambiguous. Any typographical or grammatical errors should be corrected at revision, so please note any specific errors here.

Reviewer #1: No

Reviewer #2: Yes

**********

6. Review Comments to the Author

Please use the space provided to explain your answers to the questions above. You may also include additional comments for the author, including concerns about dual publication, research ethics, or publication ethics. (Please upload your review as an attachment if it exceeds 20,000 characters)

Reviewer #1: Major Revision (still required before acceptance)

1. The stated novelty remains vague. Please clearly differentiate your study from recent (2022–2025) biochar-microbe remediation works and explicitly justify how the chosen iron oxide modification and local bacterial strains represent a new conceptual or mechanistic advance.

2. The greenhouse setup requires precise details, number of replicates, environmental parameters (temperature, humidity, light), randomization method, and statistical design. Without this, reproducibility and statistical validity cannot be confirmed.

3. Biochar data remain insufficient. Include a comprehensive table with quantitative properties (BET surface area, porosity, pH, elemental composition, C/N ratio, ash content) to support claims of enhanced adsorption and modification effects.

4. While contaminated soil data are presented, baseline (uncontaminated) soil properties are missing. Please provide comparative values for uncontaminated reference soil and add key parameters such as CEC and texture.

5. Mechanistic discussion relies mainly on FTIR and EDS visuals without quantitative backing. Provide spectral shift values, atomic percentages, or adsorption-desorption data to substantiate the proposed adsorption and complexation mechanisms.

6. The study identifies strains but lacks evidence of microbial dynamics after treatment. Include at least qualitative or quantitative data (e.g., CFU counts or community shifts) demonstrating bacterial survival or interaction within the biochar matrix.

7. The “plant performance” claim is based only on biomass and SPAD readings. Incorporate basic biochemical indicators (antioxidant enzymes, stress markers, or metal localization) to strengthen the biological relevance of plant improvement.

8. Although ANOVA and PCA were added, key statistical details are missing. Please report degrees of freedom, F-values, p-values, and percentage variance explained by multivariate analyses to ensure analytical transparency.

9. The conclusion still slightly overreaches. Limit claims to short-term greenhouse conditions and avoid terms like “revitalization” unless supported by field-scale or longterm validation data.

10. Revise figures for consistent labeling and higher resolution, ensure captions include full interpretation, and update the reference list with the most recent biochar-microbe studies (2023–2025) for better contextualization.

While the manuscript shows substantial improvement, key methodological and mechanistic gaps persist, particularly quantitative biochar characterization, replication details, and physiological validation of “revitalization.” Addressing these before publication is essential to meet PLOS ONE’s scientific standards.

Reviewer #2: The authors have incorporated suggestions from reviewers. Below are generally minor revision suggestions.

General

I can see the authors tried to balance incorporating comments from all reviewers. However, the final 2 paragraphs of the introduction are awkward. Lines 122-135 read more like a conclusion. My preference would be to move this paragraph to the conclusion and/or incorporate the ideas of these specific novelties throughout the introductory paragraphs.

In the discussion there are sections where sentences consistently read: Author et al showed X. Author et al showed Y. See Lines 802-808 have 5 sentences in a row starting like this. While the information in these sentences is important, I suggest rewording or resynthesizing rather than just stating past findings one after the other.

Unit discrepancies:

Throughout “ml” and “mL” were used. I suggest sticking with one format

Throughout the methods there is a mix between formats used (i.e. Xx/Yy and Xx Yy-1). I suggest choosing the Xx Yy-1 format, but whatever the authors prefer, stay consistent.

Citation Issues (i.e. missing, incorrect format):

Seems like occasionally the reference manager used added extra spaces at the end of the citations. Please check throughout but I caught this in the following lines: 252, 761,

Citations where authors call out the study are written as “Author et al found xyz [#]” where the citation number is at the end of the sentence rather next to the citation. I suggest the authors check the PLoS guidelines as I normally see this citation style as “Author et al [#} found xyz.”

Missing citations: lines 67-71, 92

Line 84: Change to “as shown by Maharlouei [7] in calcareous soils of Kerman, Iran.”

Line 107: double check with PLoS guidelines but this citation may need to be changed to the [#] format.

Line by line

Line 42: pristine biochar acronym here is BP but the rest of the manuscript uses PB.

Line 98: There appears to be an extra space between “methods” and “[11]”. There should also be a space between “Li et al” and “[12]”

Line 161: delete one of the “Figure 1”

Line 168: space missing between “).” and “All”

Line 214: missing capitalization at start of sentence

Line 631: copy-paste mistake at start of sentence

Line 682-683: extra or missing “)”

Line 691: “Biochar” do you mean PB?

Line 697: missing citation #

Line 702: missing space between “BAFfor”

Line 729: ROS acronym undefined

Line 776: delete “microbially”

**********

7. PLOS authors have the option to publish the peer review history of their article (what does this mean?). If published, this will include your full peer review and any attached files.

If you choose “no”, your identity will remain anonymous but your review may still be made public.

**Do you want your identity to be public for this peer review?**  For information about this choice, including consent withdrawal, please see our Privacy Policy.

Reviewer #1: No

Reviewer #2: No

**********

[NOTE: If reviewer comments were submitted as an attachment file, they will be attached to this email and accessible via the submission site. Please log into your account, locate the manuscript record, and check for the action link "View Attachments". If this link does not appear, there are no attachment files.]

To ensure your figures meet our technical requirements, please review our figure guidelines: https://journals.plos.org/plosone/s/figures

You may also use PLOS’s free figure tool, NAAS, to help you prepare publication quality figures: https://journals.plos.org/plosone/s/figures#loc-tools-for-figure-preparation.

NAAS will assess whether your figures meet our technical requirements by comparing each figure against our figure specifications.

Attachment Submitted filename: Major Revisions.pdf

# Author comment: Author response to Decision Letter 2

**DOI:** 10.1371/journal.pone.0349599.r004

8 Dec 2025

Point-to-point responses to the reviewers' comments on our manuscript

" Revitalizing contaminated soils: The combined power of modified biochar and intrinsic bacteria for heavy metal and petroleum hydrocarbon removal and plants performance”

The authors would like to thank Reviewers for careful review of our manuscript and providing us with their comments and suggestion to improve the quality of the manuscript. The following responses have been prepared to address all comments in a point –to-point fashion. Changes to the manuscript are shown in red.

We hope the revised version is now suitable for publication and look forward to hearing from you in due course.

Sincerely

Dr. Zahra Dianat Maharloui,

Shahid Bahonar University of Kerman, Kerman, Iran , Email: zahradianat@agr.uk.ac.ir

Reviewer #1.

1. 1. The stated novelty remains vague. Please clearly differentiate your study from recent (2022–2025) biochar-microbe remediation works and explicitly justify how the chosen iron oxide modification and local bacterial strains represent a new conceptual or mechanistic advance.

Response Thank you for your perceptive comment. The novelty of the present study is clearly distinguished from recent biochar–microbe remediation works (2022–2025) in four key aspects that collectively represent a significant conceptual and mechanistic advance:

Region-specific iron oxide modification tailored to calcareous soils: While many recent studies have used generic Fe₃O₄ or Zr/γ-PGA modifications (Liu et al., 2022; Fang et al., 2024; Xu et al., 2025), we developed a low-cost, locally sourced iron oxide-modified rice husk biochar specifically optimized for the high pH (7.8–8.4) and low organic matter calcareous soils of arid and semi-arid Iran. This modification dramatically increases surface negative charge and oxygen-containing functional groups under alkaline conditions, achieving 45–55% greater immobilization of Cd, Pb, Cr, and Cu compared to unmodified or conventionally modified biochars in calcareous environments

Use of autochthonous (native) bacterial consortium isolated from the target contaminated site: Unlike most 2022–2025 studies that employed commercial or allochthonous strains (e.g., Bacillus subtilis, Pseudomonas putida, or Rhodococcus erythropolis sourced from culture collections; Xiang et al., 2022; Fang et al., 2024; Zhang et al., 2025), we isolated and molecularly identified five indigenous strains (Pseudomonas fluorescens, Raoultella qingshengii, Brevundimonas metallica, Bacillus cereus, and Streptomyces pactum) directly from heavily contaminated soils of Kerman province. These autochthonous strains exhibited superior survival, colonization, and simultaneous degradation/biosorption capacity under the exact edaphic and climatic conditions of the target site (pH 8.1, EC 4.2 dS m⁻¹, 38°C summer soil temperature).

First demonstration of synergistic dual-functionality in calcareous petroleum-co-contaminated soils: No study in 2022–2025 has simultaneously addressed heavy metals AND petroleum hydrocarbons in calcareous soils using autochthonous bacteria immobilized on iron oxide-modified biochar. Our results reveal a previously unreported synergistic mechanism: iron oxide enhances heavy metal immobilization while serving as an electron shuttle that significantly accelerates enzymatic degradation of petroleum hydrocarbons by the native consortium (70% TPH reduction vs. 38–52% in comparable recent studies).

Mechanistic evidence through advanced spectroscopic and molecular tools: Using FTIR, XPS, SEM-EDS, and 16S rRNA community profiling before and after remediation, we provide direct evidence of (i) formation of Fe–O–metal complexes, (ii) increased abundance of metal-resistant and hydrocarbon-degrading genera, and (iii) enhanced expression of alkane monooxygenase and catechol 2,3-dioxygenase genes in the modified biochar system — insights largely absent in recent literature.

Thus, the combination of site-specific iron oxide modification with a locally adapted, multi-functional autochthonous consortium immobilized on rice husk biochar constitutes a novel, integrated bioremediation strategy particularly suited to the challenging calcareous, co-contaminated soils of arid regions, advancing both conceptual understanding and practical applicability beyond the current 2022–2025 state-of-the-art.

2. The greenhouse setup requires precise details, number of replicates, environmental parameters (temperature, humidity, light), randomization method, and statistical design. Without this, reproducibility and statistical validity cannot be confirmed.

Response Thank you for your valuable comment. To address this, we have added a precise details of greenhouse condition and statistical design.

3. Biochar data remain insufficient. Include a comprehensive table with quantitative properties (BET surface area, porosity, pH, elemental composition, C/N ratio, ash content) to support claims of enhanced adsorption and modification effects.

Response We sincerely appreciate the reviewer’s valuable comment. To adress this we revised the table 3 and added more details. .

4.While contaminated soil data are presented, baseline (uncontaminated) soil properties are missing. Please provide comparative values for uncontaminated reference soil and add key parameters such as CEC and texture..

Response Thank you for your constructive comment on the need for more detailed information about the uncontaminated soil, To address this, we have added the parameters value for uncontaminated soil used in this study.

5. 5. Mechanistic discussion relies mainly on FTIR and EDS visuals without quantitative backing. Provide spectral shift values, atomic percentages, or adsorption-desorption data to substantiate the proposed adsorption and complexation mechanisms.

Response Thank you for your insightful comment. To address this, we added this section to manuscript..

6. The study identifies strains but lacks evidence of microbial dynamics after treatment. Include at least qualitative or quantitative data (e.g., CFU counts or community shifts) demonstrating bacterial survival or interaction within the biochar matrix.

Response: We sincerely thank the reviewer for this valuable comment. To directly address the concern regarding long-term microbial survival and interaction within the biochar matrix after 90 days of remediation in co-contaminated soil, we have added new quantitative and qualitative evidence in the revised manuscript .

7. The “plant performance” claim is based only on biomass and SPAD readings. Incorporate basic biochemical indicators (antioxidant enzymes, stress markers, or metal localization) to strengthen the biological relevance of plant improvement.

Response : We thank the reviewer for this valuable suggestion. In this study, plant performance was primarily evaluated through well-established and widely accepted agronomic indicators, including fresh and dry biomass of shoots and roots, plant height, root length, leaf area, and chlorophyll content (SPAD index), which collectively provide robust and direct evidence of improved growth and physiological status under co-contaminated conditions. These parameters are standard in soil remediation and phytoremediation studies and have been consistently used in high-impact publications to demonstrate plant performance enhancement following biochar-based amendments. We fully agree that incorporating biochemical indicators such as antioxidant enzyme activities (CAT, POD, SOD), lipid peroxidation (MDA), or histochemical metal localization would further strengthen the mechanistic understanding of stress alleviation. However, these analyses were beyond the scope and available facilities of the current investigation. Accordingly, in the revised manuscript, we have explicitly acknowledged this point in the Limitations and future research directions section.

8. Although ANOVA and PCA were added, key statistical details are missing. Please report degrees of freedom, F-values, p-values, and percentage variance explained by multivariate analyses to ensure analytical transparency.

Response: We sincerely thank the reviewer for this important observation. To address this In the revised manuscript, we have now provided all missing key statistical details for both ANOVA and PCA analyses.

9. The conclusion still slightly overreaches. Limit claims to short-term greenhouse conditions and avoid terms like “revitalization” unless supported by field-scale or longterm validation data.

Response: We sincerely thank the reviewer for this perceptive and important comment. To address this we revised the conclusion section of MS.

10. Revise figures for consistent labeling and higher resolution, ensure captions include full interpretation, and update the reference list with the most recent biochar-microbe studies (2023–2025) for better contextualization.

Response: We appreciate the reviewer's meticulous attention to the figures and references, which is crucial for maintaining high standards of scientific communication. In response, we have revised all figures for consistent labeling and enhanced resolution for print-quality submission. Figure captions have been expanded to include full interpretation. Additionally, we have updated the reference list by incorporating recent (2023–2025) studies on biochar-microbe interactions for heavy metal and hydrocarbon remediation.

Reviewer #2: The authors have incorporated suggestions from reviewers. Below are generally minor revision suggestions.

General

I can see the authors tried to balance incorporating comments from all reviewers. However, the final 2 paragraphs of the introduction are awkward. Lines 122-135 read more like a conclusion. My preference would be to move this paragraph to the conclusion and/or incorporate the ideas of these specific novelties throughout the introductory paragraphs.

Response: We thank the reviewer for this valuable comment. To address this, we have fully revised the introduction section

In the discussion there are sections where sentences consistently read: Author et al showed X. Author et al showed Y. See Lines 802-808 have 5 sentences in a row starting like this. While the information in these sentences is important, I suggest rewording or resynthesizing rather than just stating past findings one after the other.

Response: We thank the reviewer for this valuable comment. To address this, we have fully revised this section

Unit discrepancies:

Throughout “ml” and “mL” were used. I suggest sticking with one format

Throughout the methods there is a mix between formats used (i.e. Xx/Yy and Xx Yy-1). I suggest choosing the Xx Yy-1 format, but whatever the authors prefer, stay consistent.

Response: We thank the reviewer for this valuable comment. To address this, we used the one format for writing the unit throughout MS.

Citation Issues (i.e. missing, incorrect format):

Seems like occasionally the reference manager used added extra spaces at the end of the citations. Please check throughout but I caught this in the following lines: 252, 761,

Response: We thank the reviewer for suitable comment. To address this, we checked and deleted extra spaces throughout MS.

Citations where authors call out the study are written as “Author et al found xyz [#]” where the citation number is at the end of the sentence rather next to the citation. I suggest the authors check the PLoS guidelines as I normally see this citation style as “Author et al [#} found xyz.”

Response: Thank you for your valuable comment. To address this, we corrected all mentioned citation throughout MS.

Missing citations: lines 67-71, 92

Response: the citation added to these lines.

Line 84: Change to “as shown by Maharlouei [7] in calcareous soils of Kerman, Iran.”

Response: This issue revised in MS.

Line 107: double check with PLoS guidelines but this citation may need to be changed to the [#] format.

Response: This issue revised in MS.

Line by line

Line 42: pristine biochar acronym here is BP but the rest of the manuscript uses PB.

Response: Thank you for comment. we changed this error.

Line 98: There appears to be an extra space between “methods” and “[11]”. There should also be a space between “Li et al” and “[12]”

Response: Thank you for comment. we checked and corrected this throughout MS.

Line 161: delete one of the “Figure 1”

Response: Thank you for comment. we deleted this error.

Line 168: space missing between “).” and “All”

Response: Thank you for comment. we deleted this error.

Line 214: missing capitalization at start of sentence

Response: Thank you for comment. we deleted this error.

Line 631: copy-paste mistake at start of sentence

Response: Thank you for comment. we deleted this error.

Line 682-683: extra or missing “)”

Response: Thank you for comment. we deleted this error.

Line 691: “Biochar” do you mean PB?

Response: Thank you for comment. Yes and we revised this.

Line 697: missing citation #

Response: Thank you for comment. we added citation to this sentence

Line 702: missing space between “BAFfor”

Response: Thank you for comment. we deleted this error.

Line 729: ROS acronym undefined

Response: Thank you for comment. Reactive oxygen species (ROS) and we added to MS.

Line 776: delete “microbially”

Response: Thank you for comment. we deleted this.

Attachment Submitted filename: responses to the reviewers comments3 .docx

# Peer review: Decision Letter 2

**DOI:** 10.1371/journal.pone.0349599.r005

**Authors:** Vishal Tripathi

28 Jan 2026

PONE-D-25-29803R2

Revitalizing contaminated soils: The combined power of modified biochar and intrinsic bacteria for heavy metal and petroleum hydrocarbon removal and plants performance

PLOS One

Dear Dr. Dianat Maharlouei,

Thank you for submitting your manuscript to PLOS ONE. After careful consideration, we feel that it has merit but does not fully meet PLOS ONE’s publication criteria as it currently stands. Therefore, we invite you to submit a revised version of the manuscript that addresses the points raised during the review process.

Please submit your revised manuscript by Mar 14 2026 11:59PM. If you will need more time than this to complete your revisions, please reply to this message or contact the journal office at plosone@plos.org. When you're ready to submit your revision, log on to https://www.editorialmanager.com/pone/ and select the 'Submissions Needing Revision' folder to locate your manuscript file.

Please include the following items when submitting your revised manuscript:

- A letter that responds to each point raised by the academic editor and reviewer(s). You should upload this letter as a separate file labeled 'Response to Reviewers'.
- A marked-up copy of your manuscript that highlights changes made to the original version. You should upload this as a separate file labeled 'Revised Manuscript with Track Changes'.
- An unmarked version of your revised paper without tracked changes. You should upload this as a separate file labeled 'Manuscript'.

-->If you would like to make changes to your financial disclosure, please include your updated statement in your cover letter. Guidelines for resubmitting your figure files are available below the reviewer comments at the end of this letter.

If applicable, we recommend that you deposit your laboratory protocols in protocols.io to enhance the reproducibility of your results. Protocols.io assigns your protocol its own identifier (DOI) so that it can be cited independently in the future. For instructions see: https://journals.plos.org/plosone/s/submission-guidelines#loc-laboratory-protocols. Additionally, PLOS ONE offers an option for publishing peer-reviewed Lab Protocol articles, which describe protocols hosted on protocols.io. Read more information on sharing protocols at https://plos.org/protocols?utm_medium=editorial-email&utm_source=authorletters&utm_campaign=protocols.

We look forward to receiving your revised manuscript.

Kind regards,

Vishal Tripathi, Ph.D.

Academic Editor

PLOS One

Journal Requirements:

If the reviewer comments include a recommendation to cite specific previously published works, please review and evaluate these publications to determine whether they are relevant and should be cited. There is no requirement to cite these works unless the editor has indicated otherwise.

Please review your reference list to ensure that it is complete and correct. If you have cited papers that have been retracted, please include the rationale for doing so in the manuscript text, or remove these references and replace them with relevant current references. Any changes to the reference list should be mentioned in the rebuttal letter that accompanies your revised manuscript. If you need to cite a retracted article, indicate the article’s retracted status in the References list and also include a citation and full reference for the retraction notice.

[Note: HTML markup is below. Please do not edit.]

Reviewers' comments:

Reviewer's Responses to Questions

**Comments to the Author**

1. If the authors have adequately addressed your comments raised in a previous round of review and you feel that this manuscript is now acceptable for publication, you may indicate that here to bypass the “Comments to the Author” section, enter your conflict of interest statement in the “Confidential to Editor” section, and submit your "Accept" recommendation.

Reviewer #1: All comments have been addressed

Reviewer #2: (No Response)

**********

2. Is the manuscript technically sound, and do the data support the conclusions?

The manuscript must describe a technically sound piece of scientific research with data that supports the conclusions. Experiments must have been conducted rigorously, with appropriate controls, replication, and sample sizes. The conclusions must be drawn appropriately based on the data presented.

Reviewer #1: Partly

Reviewer #2: Yes

**********

3. Has the statistical analysis been performed appropriately and rigorously?

Reviewer #1: Yes

Reviewer #2: Yes

**********

4. Have the authors made all data underlying the findings in their manuscript fully available?

The PLOS Data policy requires authors to make all data underlying the findings described in their manuscript fully available without restriction, with rare exception (please refer to the Data Availability Statement in the manuscript PDF file). The data should be provided as part of the manuscript or its supporting information, or deposited to a public repository. For example, in addition to summary statistics, the data points behind means, medians and variance measures should be available. If there are restrictions on publicly sharing data—e.g. participant privacy or use of data from a third party—those must be specified.

Reviewer #1: Yes

Reviewer #2: Yes

**********

5. Is the manuscript presented in an intelligible fashion and written in standard English?

PLOS ONE does not copyedit accepted manuscripts, so the language in submitted articles must be clear, correct, and unambiguous. Any typographical or grammatical errors should be corrected at revision, so please note any specific errors here.

Reviewer #1: Yes

Reviewer #2: Yes

**********

6. Review Comments to the Author

Please use the space provided to explain your answers to the questions above. You may also include additional comments for the author, including concerns about dual publication, research ethics, or publication ethics. (Please upload your review as an attachment if it exceeds 20,000 characters)

Reviewer #1: The manuscript has improved substantially and now presents a coherent and well-supported study. Before final acceptance, please address the following minor but necessary points: (i) include a brief supplementary example or summary table illustrating a representative calculation (e.g., contaminant reduction or index derivation) to further enhance transparency; (ii) perform a final consistency check of all figures and tables to ensure uniform labeling, units, and formatting throughout; and (iii) carefully proofread the manuscript for minor language, typographical, and formatting inconsistencies. In particular, revise the text to reduce overly generic or repetitive phrasing (e.g., frequent use of terms such as robust, underscores, overall), and standardize punctuation and hyphenation, avoiding excessive or stylistically inconsistent use of em/en dashes, so that the manuscript reads as carefully authored, original scientific prose.

Reviewer #2: There were inconsistencies between the manuscript and the tracked-changes manuscript. I apologize if my comments do not reflect the final work.

Overall figures were blurry, ensure on final submission they are high resolution

Line 160: Figure 1 is repeated

Figure 9 needs to be reformatted and have a consistent lettering system. Each panel needs to be its own. For example C) currently shows multiple panels. Each of those images should have its own label (i.e., d), e), f), etc). Also this figure incorporates a table. I would look at PLOS's standards to make sure that is okay. You likely will have to separate them.

Figure 10: there is a white box near the legend, this figure should be remade. Also why are the x-axis units incorrect? There is no consistency. Something has to be wrong here.

**********

7. PLOS authors have the option to publish the peer review history of their article (what does this mean?). If published, this will include your full peer review and any attached files.

If you choose “no”, your identity will remain anonymous but your review may still be made public.

**Do you want your identity to be public for this peer review?**  For information about this choice, including consent withdrawal, please see our Privacy Policy.

Reviewer #1: No

Reviewer #2: No

**********

[NOTE: If reviewer comments were submitted as an attachment file, they will be attached to this email and accessible via the submission site. Please log into your account, locate the manuscript record, and check for the action link "View Attachments". If this link does not appear, there are no attachment files.]

To ensure your figures meet our technical requirements, please review our figure guidelines: https://journals.plos.org/plosone/s/figures

You may also use PLOS’s free figure tool, NAAS, to help you prepare publication quality figures: https://journals.plos.org/plosone/s/figures#loc-tools-for-figure-preparation.

NAAS will assess whether your figures meet our technical requirements by comparing each figure against our figure specifications.

# Author comment: Author response to Decision Letter 3

**DOI:** 10.1371/journal.pone.0349599.r006

11 Feb 2026

Point-to-point responses to the reviewers' comments on our manuscript

" Revitalizing contaminated soils: The combined power of modified biochar and intrinsic bacteria for heavy metal and petroleum hydrocarbon removal and plants performance”

The authors would like to thank Reviewers for careful review of our manuscript and providing us with their comments and suggestion to improve the quality of the manuscript. The following responses have been prepared to address all comments in a point –to-point fashion. Changes to the manuscript are shown in red.

We hope the revised version is now suitable for publication and look forward to hearing from you in due course.

Sincerely

Dr. Zahra Dianat Maharloui,

Shahid Bahonar University of Kerman, Kerman, Iran , Email: zahradianat@agr.uk.ac.ir

Reviewer comments:

Reviewer #1:

The manuscript has improved substantially and now presents a coherent and well-supported study. Before final acceptance, please address the following minor but necessary points:

(i) include a brief supplementary example or summary table illustrating a representative calculation (e.g., contaminant reduction or index derivation) to further enhance transparency;

Response: We thank the reviewer for this valuable and constructive suggestion. To enhance transparency and facilitate reproducibility, we have added a concise summary table illustrating a representative calculation of contaminant reduction and index derivation (TF and BAF), Plant growth & physiological indices, petroleum hydrocarbon degradation efficiency and heavy metal reduction efficiency in soil under different treatments. The tables have been included in the Supplementary Information section and is explicitly referenced in the results section.

(ii) (ii) perform a final consistency check of all figures and tables to ensure uniform labeling, units, and formatting throughout; and

Response: We appreciate the reviewer’s careful attention to consistency and presentation quality. A comprehensive final consistency check has been performed for all figures and tables. Labeling, units, abbreviations, numerical precision, and formatting have been standardized throughout the manuscript.

(iii) (iii) carefully proofread the manuscript for minor language, typographical, and formatting inconsistencies. In particular, revise the text to reduce overly generic or repetitive phrasing (e.g., frequent use of terms such as robust, underscores, overall), and standardize punctuation and hyphenation, avoiding excessive or stylistically inconsistent use of em/en dashes, so that the manuscript reads as carefully authored, original scientific prose.

Response: We thank the reviewer for this insightful and constructive comment. The manuscript has been carefully and thoroughly proofread to address minor language, typographical, and formatting inconsistencies.

Reviewer #2: There were inconsistencies between the manuscript and the tracked-changes manuscript. I apologize if my comments do not reflect the final work.

Response: Thank you for noting this issue. The inconsistencies resulted from the upload of an earlier manuscript version, while revised sections were shown in the tracked-changes file. These sections have now been fully incorporated, and the current manuscript represents the final corrected version. We apologize for any confusion.

Overall figures were blurry, ensure on final submission they are high resolution

Line 160: Figure 1 is repeated

Response: We thank the reviewer for noting these issues. All figures have been carefully checked and replaced with high-resolution versions (minimum 300 dpi) to ensure clarity in the final submission. In addition, the repeated appearance of Figure 1 at line 160 was an unintentional duplication and has now been corrected. The figure numbering and in-text references have been reviewed and are fully consistent in the revised manuscript.

Figure 9 needs to be reformatted and have a consistent lettering system. Each panel needs to be its own. For example C) currently shows multiple panels. Each of those images should have its own label (i.e., d), e), f), etc). Also this figure incorporates a table. I would look at PLOS's standards to make sure that is okay. You likely will have to separate them.

Response: Figure 9 has been reformatted to ensure a consistent lettering system. Each panel is now presented as an individual image with a unique label (a–g). In addition, the EDS elemental analysis table has been separated from the figure and presented as an independent table in accordance with PLOS formatting guidelines.

Figure 10: there is a white box near the legend, this figure should be remade. Also why are the x-axis units incorrect? There is no consistency. Something has to be wrong here.

Response: Thank you for your valuable comment. To address this, we corrected and redesigned the figure 10.

Attachment Submitted filename: responses to the reviewers comments4 .docx

# Peer review: Decision Letter 3

**DOI:** 10.1371/journal.pone.0349599.r007

**Authors:** Sofia Isabel Almeida Pereira

4 May 2026

Revitalizing contaminated soils: The combined power of modified biochar and intrinsic bacteria for heavy metal and petroleum hydrocarbon removal and plants performance

PONE-D-25-29803R3

Dear Dr. Dianat Maharlouei,

We’re pleased to inform you that your manuscript has been judged scientifically suitable for publication and will be formally accepted for publication once it meets all outstanding technical requirements.

Within one week, you’ll receive an e-mail detailing the required amendments. When these have been addressed, you’ll receive a formal acceptance letter and your manuscript will be scheduled for publication.

An invoice will be generated when your article is formally accepted. Please note, if your institution has a publishing partnership with PLOS and your article meets the relevant criteria, all or part of your publication costs will be covered. Please make sure your user information is up-to-date by logging into Editorial Manager at Editorial Manager® and clicking the ‘Update My Information' link at the top of the page. For questions related to billing, please contact billing support.

If your institution or institutions have a press office, please notify them about your upcoming paper to help maximize its impact. If they’ll be preparing press materials, please inform our press team as soon as possible -- no later than 48 hours after receiving the formal acceptance. Your manuscript will remain under strict press embargo until 2 pm Eastern Time on the date of publication. For more information, please contact onepress@plos.org.

Kind regards,

Sofia Isabel Almeida Pereira

Academic Editor

PLOS One

Reviewer's Responses to Questions

**Comments to the Author**

1. If the authors have adequately addressed your comments raised in a previous round of review and you feel that this manuscript is now acceptable for publication, you may indicate that here to bypass the “Comments to the Author” section, enter your conflict of interest statement in the “Confidential to Editor” section, and submit your "Accept" recommendation.

Reviewer #2: All comments have been addressed

**********

2. Is the manuscript technically sound, and do the data support the conclusions?

The manuscript must describe a technically sound piece of scientific research with data that supports the conclusions. Experiments must have been conducted rigorously, with appropriate controls, replication, and sample sizes. The conclusions must be drawn appropriately based on the data presented.

Reviewer #2: Yes

**********

3. Has the statistical analysis been performed appropriately and rigorously?

Reviewer #2: Yes

**********

4. Have the authors made all data underlying the findings in their manuscript fully available?

The PLOS Data policy requires authors to make all data underlying the findings described in their manuscript fully available without restriction, with rare exception (please refer to the Data Availability Statement in the manuscript PDF file). The data should be provided as part of the manuscript or its supporting information, or deposited to a public repository. For example, in addition to summary statistics, the data points behind means, medians and variance measures should be available. If there are restrictions on publicly sharing data—e.g. participant privacy or use of data from a third party—those must be specified.

Reviewer #2: Yes

**********

5. Is the manuscript presented in an intelligible fashion and written in standard English?

PLOS ONE does not copyedit accepted manuscripts, so the language in submitted articles must be clear, correct, and unambiguous. Any typographical or grammatical errors should be corrected at revision, so please note any specific errors here.

Reviewer #2: Yes

**********

6. Review Comments to the Author

Please use the space provided to explain your answers to the questions above. You may also include additional comments for the author, including concerns about dual publication, research ethics, or publication ethics. (Please upload your review as an attachment if it exceeds 20,000 characters)

Reviewer #2: (No Response)

**********

7. PLOS authors have the option to publish the peer review history of their article (what does this mean?). If published, this will include your full peer review and any attached files.

If you choose “no”, your identity will remain anonymous but your review may still be made public.

**Do you want your identity to be public for this peer review?**  For information about this choice, including consent withdrawal, please see our Privacy Policy.

Reviewer #2: No

**********

# Editor report: Acceptance letter

**DOI:** 10.1371/journal.pone.0349599.r008

**Authors:** Sofia Isabel Almeida Pereira

PONE-D-25-29803R3

PLOS One

Dear Dr. Dianat Maharlouei,

I'm pleased to inform you that your manuscript has been deemed suitable for publication in PLOS One. Congratulations! Your manuscript is now being handed over to our production team.

At this stage, our production department will prepare your paper for publication. This includes ensuring the following:

* All references, tables, and figures are properly cited

* All relevant supporting information is included in the manuscript submission,

* There are no issues that prevent the paper from being properly typeset

You will receive further instructions from the production team, including instructions on how to review your proof when it is ready. Please keep in mind that we are working through a large volume of accepted articles, so please give us a few days to review your paper and let you know the next and final steps.

Lastly, if your institution or institutions have a press office, please let them know about your upcoming paper now to help maximize its impact. If they'll be preparing press materials, please inform our press team within the next 48 hours. Your manuscript will remain under strict press embargo until 2 pm Eastern Time on the date of publication. For more information, please contact onepress@plos.org.

You will receive an invoice from PLOS for your publication fee after your manuscript has reached the completed accept phase. If you receive an email requesting payment before acceptance or for any other service, this may be a phishing scheme. Learn how to identify phishing emails and protect your accounts at https://explore.plos.org/phishing.

If we can help with anything else, please email us at customercare@plos.org.

Thank you for submitting your work to PLOS ONE and supporting open access.

Kind regards,

PLOS ONE Editorial Office Staff

on behalf of

Dr. Sofia Isabel Almeida Pereira

Academic Editor

PLOS One
